# Probing coenzyme A homeostasis with semisynthetic biosensors

**DOI:** 10.1038/s41589-022-01172-7

**Published:** 2022-10-31

**Authors:** Lin Xue, Paul Schnacke, Michelle S. Frei, Birgit Koch, Julien Hiblot, Richard Wombacher, Sebastian Fabritz, Kai Johnsson

**Affiliations:** 1 Department of Chemical Biology, Max Planck Institute for Medical Research, Heidelberg, Germany; 2 MOE Key Laboratory for Cellular Dynamics, Hefei National Center for Physical Sciences at Microscale, University of Science and Technology of China, Hefei, China; 3 Institute of Chemical Sciences and Engineering, École Polytechnique Fédérale de Lausanne, Lausanne, Switzerland

**Keywords:** Metabolic pathways, Protein design, Chemical tools, Biosynthesis

## Abstract

Coenzyme A (CoA) is one of the central cofactors of metabolism, yet a method for measuring its concentration in living cells is missing. Here we introduce the first biosensor for measuring CoA levels in different organelles of mammalian cells. The semisynthetic biosensor is generated through the specific labeling of an engineered GFP–HaloTag fusion protein with a fluorescent ligand. Its readout is based on CoA-dependent changes in Förster resonance energy transfer efficiency between GFP and the fluorescent ligand. Using this biosensor, we probe the role of numerous proteins involved in CoA biosynthesis and transport in mammalian cells. On the basis of these studies, we propose a cellular map of CoA biosynthesis that suggests how pools of cytosolic and mitochondrial CoA are maintained.

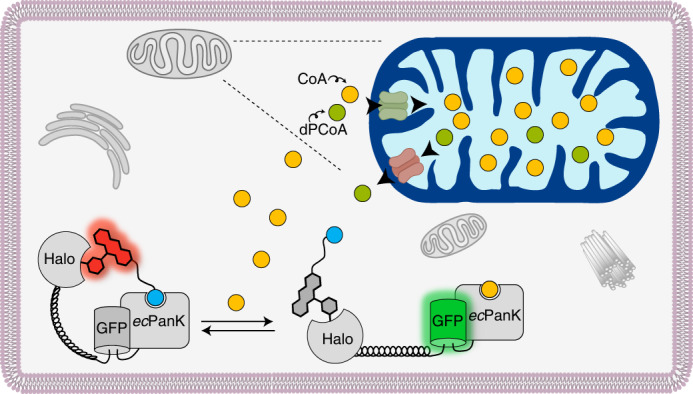

## Abstract (web summary)

Coenzyme A (CoA) is a ubiquitous and essential cofactor. A biosensor for visualizing cytosolic and mitochondrial CoA in living cells was developed to address central questions concerning CoA homeostasis.

## Main

Coenzyme A (CoA) is a ubiquitous cofactor required in a vast number of enzymatic reactions and various cellular processes, including the citric acid cycle, sterol biosynthesis, amino acid metabolism, ketone body production, and fatty acid metabolism^1–3^. CoA concentrations regulate cellular metabolism, either as substrates or as allosteric modulators^1^. Post-translational modifications of histones and other proteins with acyl-CoAs control the fate of a plethora of proteins^4^. Consequently, dysregulation of CoA levels leads to various pathologies^5^.

**Fig. 1:**
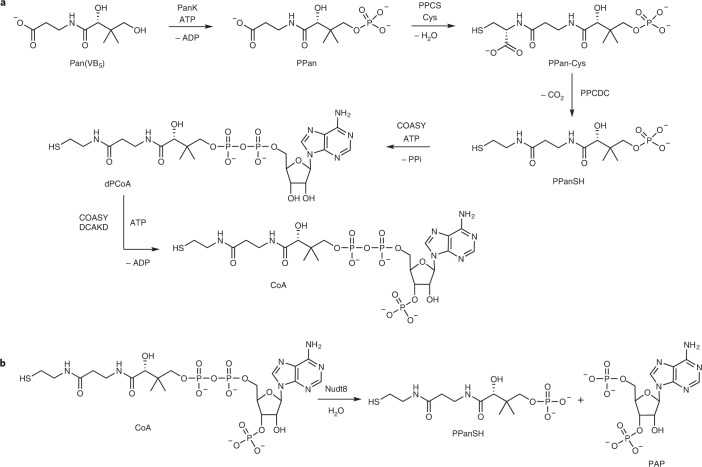
Synthesis and degradation of CoA. **a**, Biosynthetic pathway of CoA. **b**, Hydrolysis of CoA by Nudt8.

In eukaryotes, CoA is synthesized from cysteine, ATP, and the essential nutrient pantothenate, also known as vitamin B_5_^6^ (Fig. 1a). Pantothenate is phosphorylated by pantothenate kinase (PanK) to 4′-phosphopantothenat (PPan) in a first step and subsequently converted to 4′-phosphopantetheine (PPanSH) by phosphopantothenoylcysteine synthetase (PPCS) and phosphopantothenoylcysteine decarboxylase (PPCDC)^2,3^. The bifunctional CoA synthase (COASY) converts PPanSH to dephosphocoenzyme A (dPCoA) by condensing PPanSH and AMP, followed by phosphorylation of dPCoA to form CoA. In addition, mammals and other eukaryotes possess a monofunctional dephosphocoenzyme A kinase (DCAKD) that also catalyzes the final phosphorylation step of CoA biosynthesis^7^. The eukaryotic PanKs are considered to be the rate-limiting enzymes in CoA biosynthesis and their activities are allosterically regulated by acyl-CoA and CoA^8,9^. There are four active eukaryotic PanKs: PanK1α and PanK1β, which are encoded by two spliced forms of the *PANK1* gene, as well as PanK2 and PanK3. All four PanKs are homodimers possessing a highly conserved active site but have different regulatory properties and cellular localization^2,10^. PanK1β and PanK3 are localized to the cytosol, whereas PanK2 is localized to the intermembrane space (IMS) of mitochondria and PanK1α to the nucleus. Reports on the subcellular localization of COASY are conflicting, describing localization to the cytosol, to the outer mitochondrial membrane, or to the mitochondrial matrix, whereas the other enzymes of CoA biosynthesis are all reported to be localized to the cytosol^11–14^. Additionally, two mitochondrial CoA transporters, SLC25A16 and SLC25A42^15–17^, have been proposed to shuttle CoA or its biosynthetic precursor dPCoA between cytosol and mitochondria. The mitochondrial CoA pool is also believed to be regulated by Nudix hydrolase 8 (Nudt8), which degrades CoA into PPanSH and adenosine 3′,5′-diphosphate (PAP)^18^ (Fig. 1b).

CoA concentrations vary among subcellular compartments, including cytosol, mitochondria, and peroxisome^2,3^. The mitochondrial CoA pool has been reported to be the largest one, encompassing 80–95% of total CoA in liver, heart, and muscle cells^3^. However, how the different subcellular CoA pools are regulated remains unclear^3^. To answer this question, a method for the quantification of subcellular CoA levels is indispensable.

Current methods for CoA measurements are based on chromatography, mass spectrometry or enzymatic assays^19^. They require lysis of cells or isolated mitochondria and thus only provide limited information on subcellular concentrations.

Here we introduce the first biosensor for measuring free CoA concentration [CoA] in living cells. Using this sensor, we investigate the role of various enzymes and transporters relevant to CoA homeostasis and provide for the first time absolute values of free cytosolic and mitochondrial [CoA] in different cell lines.

## Results

To create a biosensor for CoA, we relied on the so-called Snifit concept^20,21^. Snifits are fusion proteins comprising a binding protein for the analyte, a self-labeling protein (SLP) and a fluorescent protein. The SLP is selectively labeled with a fluorescent ligand that intramolecularly binds to the binding protein, resulting in a ‘closed’ conformation of the sensor. The analyte can displace the intramolecular ligand in a concentration-dependent manner from the binding protein, forcing an ‘open’ conformation. In the closed conformation, the smaller distance between the fluorescent ligand and the fluorescent protein results in a higher Förster resonance energy transfer (FRET) efficiency than that in the open conformation. The FRET efficiency thus reports on the concentration of the analyte.

### Design of Snifits for CoA on the basis of bacterial PanK

**Fig. 2:**
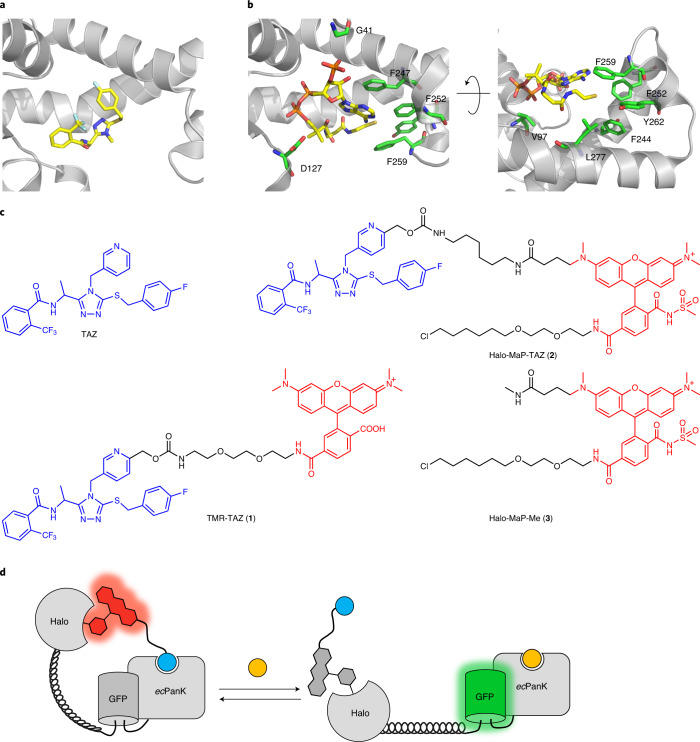
Design of CoA-Snifit. **a**, Active site of *mt*PanK with bound triazole ligand (Protein Data Bank ID: 4BFU). **b**, Active site of *ec*PanK with bound CoA (Protein Data Bank ID: 1ESM); only key residues of *ec*PanK are depicted as green sticks for clarity. **c**, Chemical structures of TAZ inhibitor and probes used in this study. **d**, Design principle of CoA-Snifit. CoA (depicted as yellow ball) and the tethered TAZ derivative (TAZ depicted as blue ball) compete for binding to *ec*PanK, shifting the sensor either to a closed or an open conformation, respectively. FRET efficiency in the closed conformation is higher than that in the open conformation.

**Extended Data Fig. 1:**
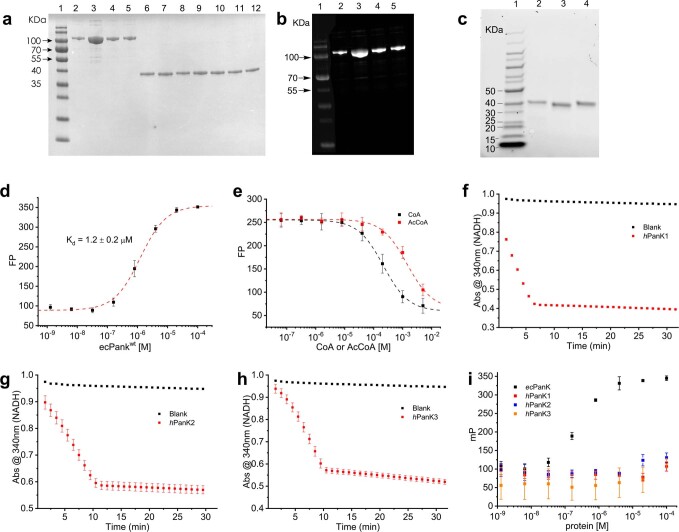
Purity analysis and characterization of ecPanKs and hPanKs. **a**. Representative Coomassie staining after protein SDS-PAGE (n = 3 independent experiments). Lane 1: protein marker, lane 2: CoA-Snifit^V97T^, lane 3: CoA-Snifit^G41^, lane 4: CoA-Snifit^G41S^, lane 5: CoA-Snifit^G41N^, lane 6: *ec*PanK^WT^, lane 7: *ec*PanK^F252Y^, lane 8: *ec*PanK^L277F^, lane 9: *ec*PanK^L277Y^, lane 10: *ec*PanK^L277W^, lane 11: *ec*PanK^F252Y,L277W^, lane 12: *ec*PanK^D127A,F252Y,L277W^. **b**. Representative in-gel fluorescence scan after SDS-PAGE (n = 3 independent experiments) of Halo-SiR labeled sensor proteins, Lane 1: protein marker, lane 2: CoA-Snifit^V97T^, lane 3: CoA-Snifit^G41^, lane 4: CoA-Snifit^G41S^, lane 5: CoA-Snifit^G41N^. **c**. Representative Coomassie staining (n = 3, independent experiments) of recombinant *h*PanKs expressed in *E. coli*. Lane 1: protein marker, lane 2: *h*PanK1, lane 3: *h*PanK2, lane 4: *h*PanK3. **d**. Fluorescence polarization responses (FP, mean ± s.d., n = 3 independent replicates) of 50 nM TMR-TAZ as a function of *ec*PanK^WT^ concentration in PBS. **e**. Fluorescence polarization responses (mean ± s.d., n = 3 independent replicates) of 50 nM TMR-TAZ and 20 μM *ec*PanK^WT^ in the presences of different CoA or AcCoA concentrations. The data (mean ± s.d., n = 3 independent replicates) are fitted to a single-site binding isotherm (dashed line). **f**-**h**. Activity assay for *h*PanK1 (**f**), *h*PanK2 (**g**), and *h*PanK3 (**h**) in activity buffer. The absorption (mean ± s.d., n = 3 independent replicates) are recorded at 340 nm at room temperature over 30 min. The titration results showed that all three recombinant *h*PanK isoforms were active. **i**. Fluorescence polarization responses (mean ± s.d., n = 3 independent replicates) of 50 nM TMR-TAZ as a function of *ec*PanK^WT^, *h*PanK1, *h*PanK2, and *h*PanK3 concentration in PBS. TMR-TAZ only binds *ec*PanK^WT^ but not the three mammalian PanKs. Note that only the catalytic core domains of *h*PanK1 and *h*PanK2 were expressed according to the previous report^60^. Source data

We focused on PanKs as binding proteins for the generation of a CoA-Snifit. Their activity is regulated by feedback inhibition of acyl-CoA and CoA^22,23^. Specific inhibitors for bacterial PanKs have been developed that do not inhibit mammalian PanKs. We therefore chose a bacterial PanK from *Escherichia coli* (*ec*Pank) as the CoA-binding protein for the generation of a Snifit for CoA. One family of inhibitors for bacterial PanKs are triazoles such as the triazole TAZ^24,25^, which competes with pantothenate, CoA, and ATP for binding (Fig. 2a–c). On the basis of an available crystal structure of PanK from *Mycobacterium tuberculosis* (*mt*PanK) with a triazole inhibitor bound to its active site^24^ (Fig. 2a), we designed and synthesized a derivative of the inhibitor linked to the fluorophore tetramethylrhodamine (TMR-TAZ (**1**); Fig. 2c). TMR-TAZ binds to *ec*Pank with a *K*_d_ of 1.2 ± 0.2 μM, as determined by fluorescence polarization (Extended Data Fig. 1a,b,d). Furthermore, CoA competes with TMR-TAZ for binding to *ec*PanK (Extended Data Fig. 1e). We also verified that none of the human PanKs (*h*PanKs) showed any binding to TMR-TAZ (Extended Data Fig. 1c,f–i).

**Extended Data Fig. 2:**
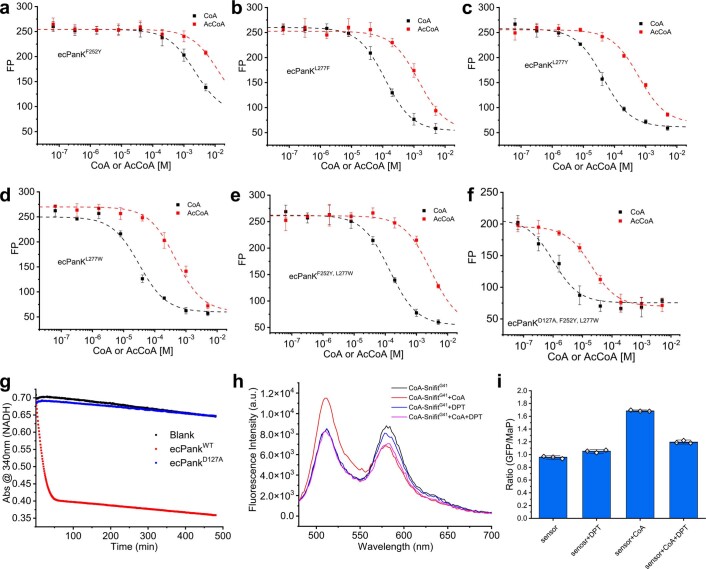
In vitro characterization of ecPanK variants and CoA-Snifits. **a-f**. Fluorescence polarization responses of 50 nM TMR-TAZ and 20 μM *ec*PanK^F252Y^ (**a**), 20 μM *ec*PanK^L277F^ (**b**), 20 μM *ec*PanK^L277Y^ (**c**), 20 μM *ec*PanK^L277W^ (**d**), 20 μM *ec*PanK^F252Y,L277W^ (**e**), and 100 μM *ec*PanK^D127A,F252Y,L277W^ (**f**) as a function of CoA or AcCoA concentration. The data (mean ± s.d., n = 3 independent replicates) are fitted to a single-site binding isotherm (dashed line). **g**. Enzymatic assay of *ec*PanK proteins. Activity of *ec*PanK^WT^ and *ec*PanK^D127A^ is measured in activity buffer. The absorption was recorded at 340 nm at room temperature over 500 min. The dead mutant *ec*PanK^D127A^ showed neglectable activity compared with the wild type *ec*PanK^WT^. **h**. Emission spectra of CoA-Snifit^G41^ upon the titration of 20 μM CoA in the absence or presence of 500 μM di-2-pyridyl thiocarbonate (DPT) in PBS. **i**.The ratio (GFP/MaP) responses (mean ± s.d., n = 3 independent replicates) of CoA-Snifit^G41^ upon the titration of 20 μM CoA in the absence or presence of 500 μM DPT. Source data

To generate a CoA-Snifit, we then fused *ec*Pank to HaloTag for the attachment of a fluorescent TAZ ligand and to a superfolder GFP (sfGFP) as FRET donor (Fig. 2d). To maximize the dynamic range of the sensor, we attempted to decrease the distance between the FRET pair in the closed state of the sensor by circular permutation of *ec*PanK (Methods). sfGFP was fused to circular permutated *ec*PanK and HaloTag was connected to sfGFP by a (EAAAK)_5_ linker, ensuring a large distance between the fluorophores in the open state (Fig. 2d). We additionally introduced mutations into *ec*PanK to increase its selectivity for CoA over acetyl-CoA (AcCoA) (Methods; Extended Data Fig. 2a–f and Supplementary Table 1). Furthermore, the catalytic activity of *ec*PanK was abolished by implementing a D127A mutation in the active site (Extended Data Fig. 2g). The resulting fusion protein HaloTag–(EAAAK)_5_–sfGFP–*ec*PanK, which lacks the fluorescent ligand, in the following text is abbreviated as apo-CoA-Snifit^G41^.

**Fig. 3:**
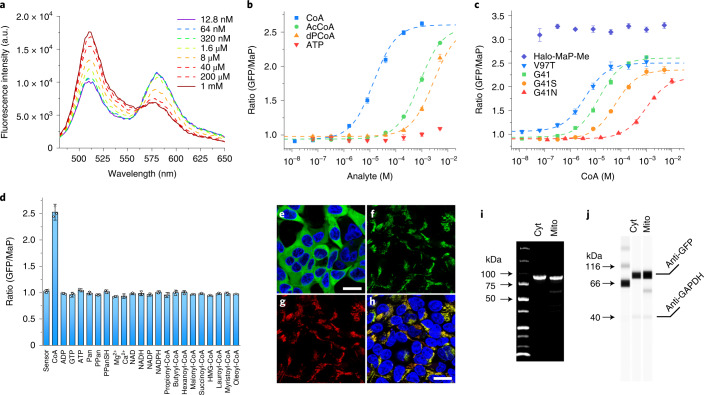
Characterization of CoA-Snifit. **a**, Fluorescence emission spectra of CoA-Snifit^G41^ in response to different CoA concentrations (12.8 nM to 1 mM) in PBS. **b**, FRET ratio (*F*_510_/*F*_580 nm_) of CoA-Snifit^G41^ (mean ± s.d., *n* = 3 independent replicates) in response to different concentrations of analyte. CoA (blue), AcCoA (green), dPCoA (orange), and ATP (red). a.u., arbitrary units. **c**, FRET ratio (*F*_510_/*F*_580 nm_) of different CoA-Snifits (mean ± s.d., *n* = 3 independent replicates) in response to CoA concentrations. **d**, The FRET ratio (*F*_510_/*F*_580 nm_) of CoA-Snifit^G41^ (mean ± s.d., *n* = 3 independent replicates) in the presence of 1 mM CoA, ATP, Pan, PPan, PPanSH, Mg^2+^, Ca^2+^, 100 μM ADP, GTP, NAD, NADH, NADP, NADPH, 20 μM propionyl-CoA, butyryl-CoA, hexanoyl-CoA, malonyl-CoA, succinyl-CoA, 3-hydroxy-3-methylglutaryl (HMG-CoA), 1 μM lauroyl-CoA, myristoyl-CoA, and oleoyl-CoA in PBS. **e**, Representative fluorescence images (*n* = 3 independent samples) of HEK293 cells stably expressing cytosolic CoA-Snifit^V97T^ (green) co-stained with Hoechst 33342 (blue). Scale bar, 20 μm. **f**–**h**, Representative fluorescence images (*n* = 3 independent samples) of HEK293 cells stably expressing mitochondrial CoA-Snifit^G41S^ (green) co-stained with Hoechst 33342 (blue) and MitoTracker Red CMXRos (red). **f**, Emission of GFP. **g**, Emission of MitoTracker Red CMXRos. **h**, Merged image of emission of Hoechst 33342, GFP, and MitoTracker Red CMXRos. Scale bar, 20 μm. **i**, Representative in-gel fluorescence (*n* = 2 independent samples) of the cell lysate (*n* = 3 independent experiments) from HEK293 cells expressing sensor protein of cytosolic CoA-Snifit^V97T^ and mitochondrial CoA-Snifit^G41S^ labeled with Halo-SiR, respectively. **j**, Representative western blot analysis (*n* = 2 independent experiments) of the cell lysate from unlabeled HEK293 cells expressing CoA-Snifit^V97T^ and CoA-Snifit^G41S^. The housekeeping gene, GAPDH, was used as the loading control. Source data

We synthesized Halo-MaP-TAZ (**2**) (Fig. 2c), which contains a chloroalkane for attachment to HaloTag and a fluorogenic TMR derivative that is based on the so-called MaP dyes^26^. MaP dyes become fluorescent only upon binding to HaloTag and thus decrease the background fluorescence from unbound dye. Furthermore, MaP dyes possess very high cell permeability. Labeling of apo-CoA-Snifit^G41^ with Halo-MaP-TAZ resulted in semisynthetic, holo-CoA-Snifit^G41^, in which the FRET efficiency was dependent on the concentration of CoA (Fig. 3a,b). The maximum change in emission ratio (Δ*R* = *R*_max_/*R*_min_) of this sensor was 2.8-fold with a concentration of half-maximal ratio change (*c*_50_) value for CoA of 13.9 ± 1.9 μM (Supplementary Table 2). By contrast, apo-CoA-Snifit^G41^ labeled with a compound lacking the triazole moiety (Halo-MaP-Me (**3**); Fig. 2c), showed no FRET ratio change upon addition of CoA (Fig. 3c). Removal of CoA restored the FRET ratio of the closed sensor, confirming that CoA-Snifit^G41^ binds CoA in a reversible manner (Extended Data Fig. 2h,i).

### Engineering and characterization of CoA-Snifit

The cellular [CoA] is reported to fall within micromolar to millimolar range^3^. To cover this entire range, we attempted to generate CoA-Snifits with different sensitivities. Residue G41 of *ec*PanK is close to the binding site of the 3′-phosphate group of CoA but has no obvious interactions with TAZ (Fig. 2a,b). The mutations G41S (yielding CoA-Snifit^G41S^) and G41N (yielding CoA-Snifit^G41N^), shifted the *c*_50_ to 58.9 ± 5.6 and 957 ± 132 μM, respectively (Fig. 3c and Supplementary Table 2). The mutation V97T (yielding CoA-Snifit^V97T^), which we expected to weaken the affinity of *ec*PanK for TAZ but not for CoA (Fig. 2a,b), lowered the *c*_50_ for CoA to 2.4 ± 0.2 μM (Fig. 3c). None of the mutations had a strong impact on the dynamic range (Fig. 3c and Supplementary Table 2). The different CoA-Snifits thus enable us to cover CoA concentrations from micromolar to millimolar.

**Extended Data Fig. 3:**
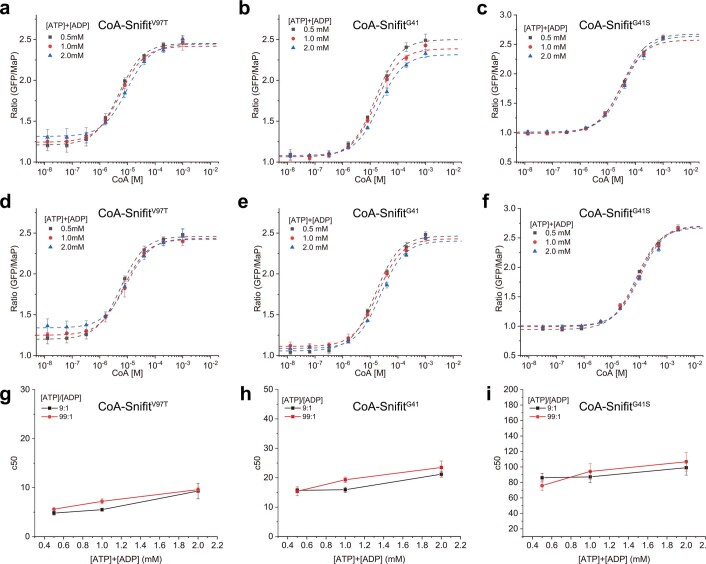
Effect of ATP and ADP on the responses of CoA-Snifits. **a-c**. the ratio (GFP/MaP) responses (mean ± s.d., n = 3 independent replicates) as a function of different CoA concentrations in the presence of different total ATP and ADP concentrations with [ATP]/[ADP] = 9:1. **d-f**. the ratio (GFP/MaP) responses (mean ± s.d., n = 3 independent replicates) as a function of different CoA concentrations in the presence of different total ATP and ADP concentrations with ratio of [ATP]/[ADP] = 99:1. **g-i**. c_50_ (mean ± s.d., n = 3 independent replicates) as a function of different total ATP and ADP concentrations. Source data

Next, we evaluated the specificity of the CoA-Snifits against other metabolites. ATP is a substrate of *ec*PanK and ADP is one of its products. The free cellular ATP concentration is around 1 mM and much higher than that of ADP^27–29^. Neither ATP nor ADP at physiologically relevant concentrations could efficiently compete with tethered TAZ ligand for binding to CoA-Snifit (Fig. 3b,d). While the sum of physiologically relevant adenosine nucleotides (that is ATP, ADP, and AMP) in cells is generally kept constant, the ratio of [ATP]/[ADP] can change remarkably^30,31^. ATP levels have been reported to change up to 50% after interruption of glycolysis through incubation with 2-deoxy-d-glucose (2-DG). To investigate how changes in [ATP] + [ADP] and [ATP]/[ADP] affect the sensor readout, we performed CoA titrations of our CoA-Snifits at concentrations of [ATP] + [ADP] ranging from 0.5 mM, 1 mM, and 2 mM, with [ATP]/[ADP] ratios of either 9 or 99. In these experiments, fourfold changes in [ATP] + [ADP] led to an at most 25% change in *R*_min_ and *R*_max_ values and to a change up to 50% change in *c*_50_ values for CoA (Extended Data Fig. 3 and Supplementary Table 3). Furthermore, tenfold changes in the [ATP]/[ADP] resulted in a maximum change of 25% in *c*_50_ values for CoA (Supplementary Table 3). Assuming that CoA-Snifits are used under conditions where any changes in basal ATP and ADP levels would be less than those observed when, for example, disrupting glycolysis with 2-DG, the observed effects of ATP/ADP on CoA-Snifits should not interfere with CoA measurements.

**Extended Data Fig. 4:**
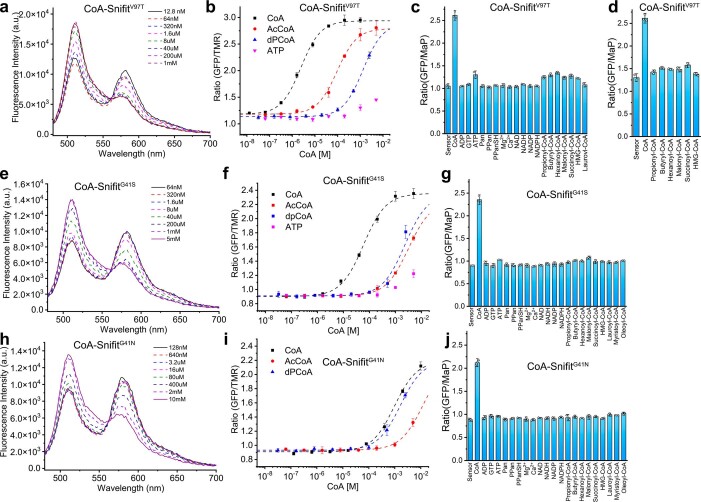
Specificity test of CoA-Snifits. **a**. Emission spectra of CoA-Snifit^V97T^ upon the titration with increasing concentrations of CoA in PBS. **b**. The ratio (GFP/MaP) responses (mean ± s.d., n = 3 independent replicates) of 100 nM sensor as a function of different CoA (black), AcCoA (red), dPCoA (blue), and ATP (magenta) concentrations in PBS. **c**. The ratio (GFP/MAP) responses (mean ± s.d., n = 3 independent replicates) of 100 nM sensor in the presence of 1 mM CoA, 100 μM ADP, 100 μM GTP, 1 mM ATP, Pan, PPan, PPanSH, Mg^2+^, Ca^2+^, 100 μM NAD, NADH, NADP, NADPH, 10 μM propionyl-CoA, butyryl-CoA, hexanoyl-CoA, malonyl-CoA, succinoyl-CoA, HMG-CoA, and 1 μM lauroyl-CoA, myristoyl-CoA, oleoyl-CoA in PBS. **d**. The ratio (GFP/MAP) responses (mean ± s.d., n = 3 independent replicates) of 100 nM sensor in PBS containing 1 mM ATP with 1 mM CoA, 10 μM propionyl-CoA, butyryl-CoA, hexanoyl-CoA, malonyl-CoA, succinoyl-CoA, or HMG-CoA. **e**. Emission spectra of CoA-Snifit^G41S^ upon the titration with increasing concentrations of CoA in PBS. **f**. The ratio (GFP/MaP) responses (mean ± s.d., n = 3 independent replicates) of 100 nM sensor as a function of different CoA (black), AcCoA (red), dPCoA (blue), and ATP (magenta) concentrations in PBS. **g**. The ratio (GFP/MAP) responses (mean ± s.d., n = 3 independent replicates) of 100 nM sensor in the presence of 5 mM CoA, 100 μM ADP, 100 μM GTP, 1 mM ATP, Pan, PPan, PPanSH, Mg^2+^, Ca^2+^, 100 μM NAD, NADH, NADP, NADPH, 100 μM propionyl-CoA, butyryl-CoA, hexanoyl-CoA, malonyl-CoA, succinoyl-CoA, HMG-CoA, and 10 μM lauroyl-CoA, myristoyl-CoA, oleoyl-CoA in PBS. **h**. Emission spectra of CoA-Snifit^G41N^ upon the titration with increasing concentrations of CoA in PBS. **i**. The ratio (GFP/MaP) responses (mean ± s.d., n = 3 independent replicates) of 100 nM sensor as a function of different CoA (black), AcCoA (red), and dPCoA (blue) concentrations in PBS. **j**. The ratio (GFP/MAP) responses (mean ± s.d., n = 3 independent replicates) of 100 nM sensor in the presence of 10 mM CoA, 100 μM ADP,100 μM GTP, 1 mM ATP, Pan, PPan, PPanSH, Mg^2+^, Ca^2+^, 100 μM NAD, NADH, NADP, NADPH, 100 μM propionyl-CoA, butyryl-CoA, hexanoyl-CoA, malonyl-CoA, succinyl-CoA, HMG-CoA, and 10 μM lauroyl-CoA, myristoyl-CoA, oleoyl-CoA in PBS. Source data

The *c*_50_ values for AcCoA, the most abundant CoA derivative^32^, of our CoA-Snifits were at least 11.7-fold higher than the corresponding values for CoA (Fig. 3b and Supplementary Table 2). Ratios of the total cellular concentrations [CoA]/[AcCoA] have been reported to be around 1 or higher^33^, suggesting that changes in [AcCoA] should not interfere with CoA-Snifit-based measurements of cellular [CoA]. The concentration of the cellular acyl-CoA pool has been reported to be below that of AcCoA^4,34^. Long-chain fatty acyl-CoA are tightly bound to the acyl-CoA-binding protein (ACBP) and their free concentrations are usually kept at very low levels (<200 nM)^35^. For CoA-Snifit^G41^, CoA-Snifit^G41S^ and CoA-Snifit^G41N^ acyl-CoA derivatives at micromolar concentrations did not result in detectable ratio changes (Fig. 3b and Extended Data Fig. 4g,j). For CoA-Snifit^V97T^, a 20% increase in the emission ratio in the presence of 10 μM short-chain acyl-CoA thioesters was detected (Extended Data Fig. 4a–d). However, this effect was reduced in the presence of 1 mM ATP, which is a typical concentration for ATP in cells.

The c_50_ values of the CoA-Snifits for dPCoA were at least 39.7-fold higher than those for CoA (Fig. 3b and Extended Data Fig. 4b,f), except for the low-affinity sensor CoA-Snifit^G41N^, which showed only a 1.7-fold selectivity (Extended Data Fig. 4i and Supplementary Table 2). The decreased selectivity of CoA-Snifit^G41N^ can be rationalized by considering that the mutation G41N results in unfavorable interactions with the 3′-phosphate group of CoA. However, as the total cellular concentrations of dPCoA are reported to be 30 times lower than those of CoA^36^, we believe that dPCoA should not affect the cellular application of CoA-Snifits.

Pantothenate, PPan, PPanSH, GTP, ADP, other adenosine-based metabolites, Ca^2+^, and Mg^2+^ at physiologically relevant concentrations did not affect the response of the sensors (Fig. 3d and Extended Data Fig. 4c,g,j).

**Extended Data Fig. 5:**
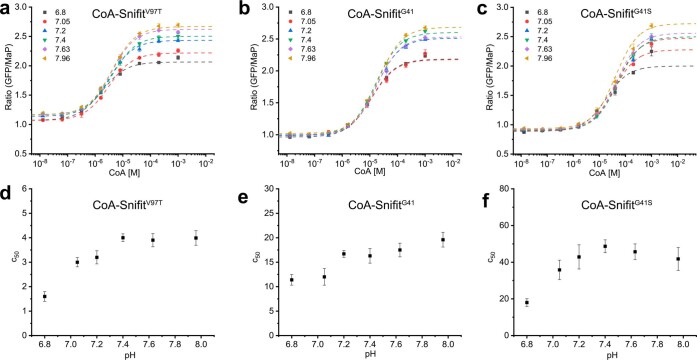
Effect of pH on the responses of CoA-Snifits. **a-c**. the ratio (GFP/MaP) responses (mean ± s.d., n = 3 independent replicates) of 100 nM CoA-Snifit^V97T^ (**a**), CoA-Snifit^G41^ (**b**), and CoA-Snifit^G41S^ (**c**) as a function of CoA concentrations at the indicated pH. **d-f**. The c_50_ (mean ± s.d., n = 3 independent replicates) of CoA-Snifit^V97T^ (**d**), CoA-Snifit^G41^ (**e**), CoA-Snifit^G41S^ (**f**) as a function of pH. Source data

To evaluate the pH sensitivity of the CoA-Snifits, we investigated the CoA-response of the sensors at different physiologically meaningful pH values. Between pH 7.2 and 8.0 the *R*_max_ and *R*_min_ values of the sensors as well as the calculated *c*_50_ values showed no major pH sensitivity (Extended Data Fig. 5). We also tested the response kinetic of the CoA-Snifits by recording the FRET ratio over time after adding saturating concentrations of CoA to CoA-Snifits. The opening of the sensors was complete within 1–2 min (Supplementary Fig. 1), suggesting that they are suitable for recording changes in [CoA] within minutes.

**Extended Data Fig. 6:**
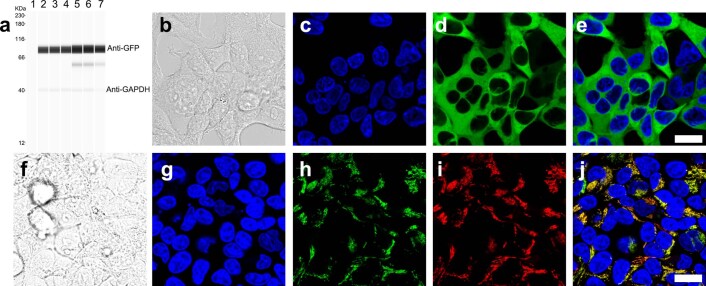
Expression and subcellular localization of the sensor proteins. **a**. Representative western blot analysis (n = 2 independent experiments) of the lysates of HEK293 cells stably expressing apo-CoA-Snifits. Small impurity bands (< 15%) were observed, possibly due to the degradation of the sensor protein. Lane 1: biotinylated ladder, lane 2: cyto-CoA-Snifit^G41^, lane 3: cyto-CoA-Snifit^G41N^, lane 4: cyto-CoA-Snifit^V97T^, lane 5: mito-CoA-Snifit^G41N^, lane 6: mito-CoA-Snifit^G41^, lane 7: mito-CoA-Snifit^G41S^. **b-j**. Testing the subcellular localization of unlabeled sensor protein of cytosolic CoA-Snifit^V97T^ (**b-e**) and mitochondrial CoA-Snifit^G41S^ **(f**-**j)** in living HEK293 cells. The data are shown as representative images of n = 3 independent samples. **b** and **f**. bright field. **c** and **g**. emission of Hoechst 33342. **d** and **h**. emission of GFP. **i**. emission of MitoTracker Red CMXRos. **e**. Merged image of **c** and **d**, Scale bar: 20 μm. **j**. Merged image of **g**, **h**, and **i**, Scale bar: 20 μm. Source data

**Extended Data Fig. 7:**
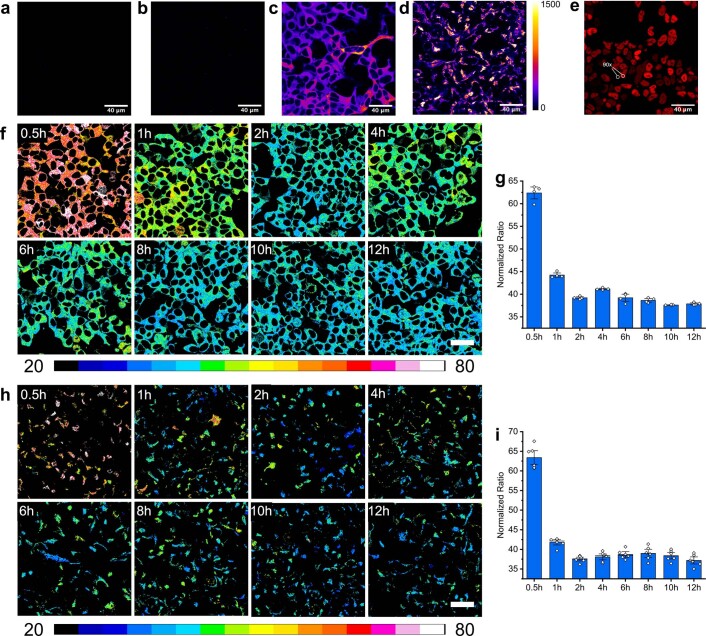
Labeling specificity of Halo-MaP-TAZ in living cells. **a-e**. Evaluating the labeling background of the ligand. The cells were labeled with 1 μM Halo-MaP-TAZ for 12 h and the emission was collected with λ_ex_ = 535 nm and λ_em_ = 560 − 620 nm. **a**. HEK293 cells without labeling. Scale bar: 40 μm. **b**. HEK293 cells incubated with 1 μM Halo-MaP-TAZ for 12 h. Scale bar: 40 μm. **c**. HEK293 cells stably expressing cytosolic sensor protein of CoA-Snifit^V97T^ labeled with 1 μM Halo-MaP-TAZ for 12 h. Scale bar: 40 μm. **d**. HEK293 cells stably expressing mitochondrial sensor protein of CoA-Snifit^G41S^ labeled with Halo-MaP-TAZ for 12 h. Scale bar: 40 μm. **e**. HEK293 cells expressing a nuclear Halo-SNAP-NLS fusion protein were labeled with 1 μM Halo-MaP-TAZ for 12 h and the emission was collected with λ_ex_ = 535 nm, λ_em_ = 560 − 620 nm. Scale bar: 40 μm. The average fluorescence intensity ratio between the nuclear and cytosolic signal (F_nuc_/F_cyt_) is 90, indicating very low background labeling of the probe. The data were shown as representative images of n = 3 independent samples. **f**. Representative ratio images of the cytosolic sensor protein of CoA-Snifit^V97T^ labeled with Halo-MaP-TAZ for 0.5, 1, 2, 4, 6, 8, 10, 12 h. Scale bar: 40 μm, ratio bar: 20 − 80. **g**. The normalized ratio values were calculated from **f** and shown as mean ± s.d., n = 4 field of views (FOVs) over 4 independent samples with > 50 cells per FOV. The normalized FRET ratio values were calculated for each FOV. **h**. Representative ratio images of the mitochondrial sensor protein of CoA-Snifit^G41S^ labeled with Halo-MaP-TAZ for 0.5, 1, 2, 4, 6, 8, 10, 12 h. Scale bar: 40 μm, ratio bar: 20 − 80. **i**. The normalized ratio values were calculated from **h** and shown as mean ± s.d., n = 6 field of views (FOVs) over 6 independent samples with > 50 cells per FOV. Source data

For applications in cells, CoA-Snifits were stably expressed in the cytosol and mitochondrial matrix of HEK293 cells (Fig. 3e–j and Extended Data Fig. 6). Expression of the sensors did not affect total cellular CoA levels, as determined by liquid chromatography–tandem mass spectrometry measurements (Supplementary Fig. 2). Incubating cells for 12 h with 1 μM Halo-MaP-TAZ resulted in specific labeling (Extended Data Fig. 7a–e). The ratio of fluorescence intensities of MaP dye and GFP remained constant after labeling for 6 h (Extended Data Fig. 7f–i), and the labeling efficiency of the probe (1 μM) after labeling for 12 h was estimated to be larger than 95% (Supplementary Figs. 3–5). After labeling with the probe, the ratiometric signal readout was stable for at least 2 h, which is a prerequisite for live measurements of CoA over time (Supplementary Fig. 6).

Overall, the high specificity and relatively low pH sensitivity of the CoA-Snifits as well as their efficient labeling in cells make them well suited for probing cytosolic and mitochondrial CoA levels in living cells.

### CoA biosynthesis and metabolism in HEK293 cells

We investigated how overexpression or knockdown of proteins involved in CoA biosynthesis and degradation affect free [CoA] in the cytosol and mitochondria of HEK293 cells. For these experiments, we chose CoA-Snifit^V97T^ and CoA-Snifit^G41S^ as their *c*_50_ values for CoA best matched cytosolic and mitochondrial [CoA], respectively (Supplementary Fig. 7). To minimize the influence of environmental factors such as pH we normalized the net FRET values against the net FRET of the fully open sensor under each tested condition (Methods), which was obtained by labeling the corresponding apo-CoA-Snifit with Halo-MaP-Me.

**Fig. 4:**
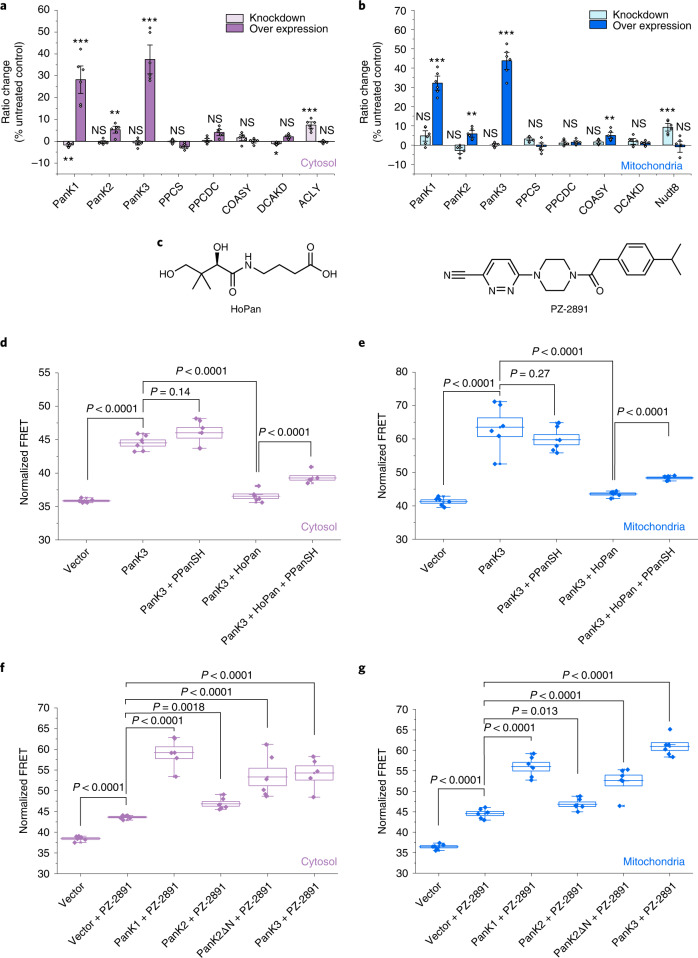
Regulation of CoA in HEK293 cells. **a**,**b**, Fluorescence ratio changes of cytosolic CoA-Snifit^V97T^ (**a**) and mitochondrial CoA-Snifit^G41S^ (**b**) under the gene knockdown and overexpression conditions. Cells transfected with non-targeting esiRNA for firefly luciferase (siFLUC) were used as the negative control for gene knockdown. Cells, transfected with empty vector, were used as the negative control for gene overexpression. Ratio changes are percentage of normalized FRET values to control, (FRET − FRET_control_)/FRET_control_. They are presented as mean ± s.e.m., *n* = 6 FOVs over 4 independent samples with >50 cells per FOV. **c**, Chemical structures of HoPan and PanK activator PZ-2891. **d**,**e**, Regulation of PanKs by HoPan and effect on cytosolic (**d**) and mitochondrial (**e**) normalized FRET ratio. Cells transfected with empty vector were used as the negative control. The data are presented as mean ± s.e.m., *n* = 6 FOVs over 4 independent samples with >50 cells per FOV. **f**,**g**, Activation of PanKs by PZ-2891 and effect on cytosolic (**f**) and mitochondrial (**g**) normalized FRET ratio. Cells, transfected with empty vector and treated with 0.05% (v/v) DMSO, were used as the negative control. The box plots represent the s.e.m. at the lower and upper box limits and the mean as the middle bar. *n* = 5 or 6 FOVs over 4 independent samples with >50 cells per FOV. The whiskers extend to ±1.5× the interquartile range beyond the limits of the boxes, respectively. The precise *n* and *P* values are listed in the Supplementary Data. *P ≤ 0.05, **P ≤ 0.01, ***P ≤ 0.001. NS, not significant (P > 0.05). Two-tailed unpaired *t*-test. Source data

**Extended Data Fig. 8:**
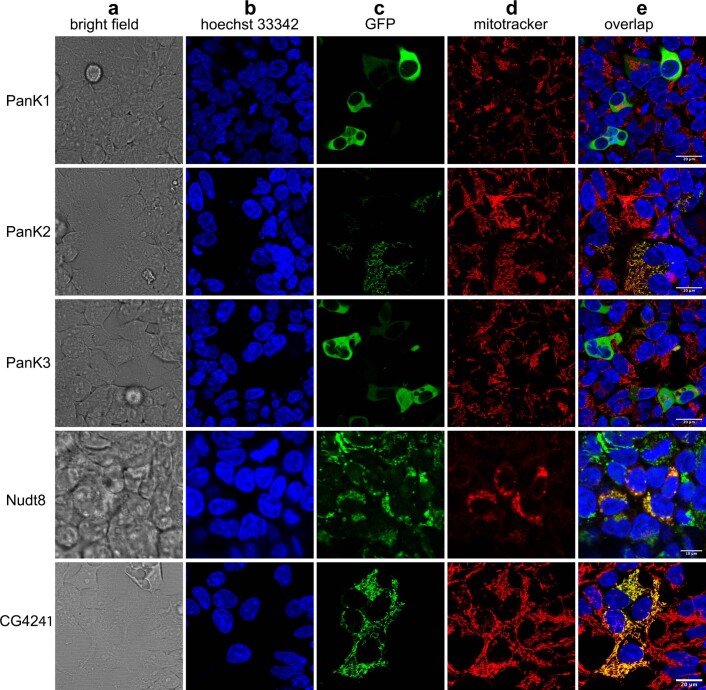
Subcellular localization of overexpressed proteins. Overexpression of GFP fusion proteins in HEK293 cells which were co-stained with 1 μg/mL Hoechst 33342 and 100 nM MitoTracker Red CMXRos. The data are shown as representative images of n = 3 independent samples. **a**. Bright field. **b**. Emission of Hoechst 33342. **c**. Emission of GFP. **d**. Emission of MitoTracker Red CMXRos. **e**. Merged image of **b**, **c**, and **d**. Scale bar: 20 μm for PanK1, PanK2, PanK3, and CG4241. Scale bar: 10 μm for Nudt8. Source data

**Extended Data Fig. 9:**
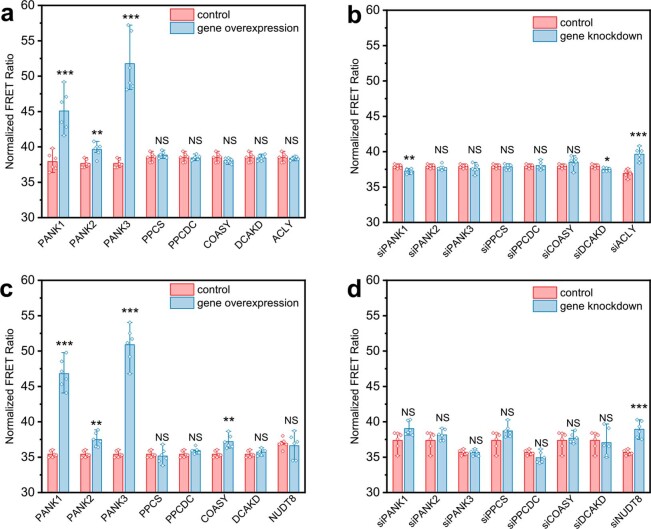
Influence of gene overexpression or knockdown of proteins involved in CoA biosynthesis on cytosolic (a and b) and mitochondrial (c and d) FRET ratio. Cells, transfected with non-targeting esiRNA for firefly luciferase (siFLUC), were used as the negative control for gene knockdown. Cells, transfected with empty vector, were used as the negative control for gene overexpression. The data are presented as mean ± s.e.m., n = 6 field of views (FOVs) over 4 independent samples with > 50 cells per FOV. **P ≤ 0.01, ***P ≤ 0.001, NS p > 0.05, two-tailed unpaired t-test. *p* values for **a** (left to right): *p* < 0.0001 (PANK1), *p* = 0.0014 (PANK2), *p* < 0.0001 (PANK3), *p* = 0.32 (PPCS), *p* = 0.81 (PPCDC), *p* = 0.16 (COASY), *p* = 0.81 (DCAKD), *p* = 0.69 (ACLY). *p* values for **b** (left to right): *p* < 0.004 (siPANK1), *p* = 0.40 (siPANK2), *p* = 0.33 (siPANK3), *p* = 0.77 (siPPCS), *p* = 0.63 (siPPCDC), *p* = 0.12 (siCOASY), *p* = 0.014 (siDCAKD), *p* < 0.0001 (siACLY). *p* values for **c** (left to right): *p* < 0.0001 (PANK1), *p* = 0.0011 (PANK2), *p* < 0.0001 (PANK3), *p* = 0.06 (PPCS), *p* = 0.074 (PPCDC), *p* = 0.0018 (COASY), *p* = 0.29 (DCAKD), *p* = 0.68 (NUDT8). *p* values for **d** (left to right): *p* > 0.05 (siPANK1), *p* = 0.22 (siPANK2), *p* = 0.98 (siPANK3), *p* = 0.06 (siPPCS), *p* = 0.07 (siPPCDC), *p* = 0.62 (siCOASY), *p* = 0.74 (siDCAKD), *p* < 0.0001 (siNUDT8). Source data

Overexpression of human PanK1 (a truncated variant of PanK1*α*) and PanK3 led to strong, 20–40% increases in normalized FRET ratios both in the cytosol and mitochondria, suggesting an increase in free [CoA] in both compartments (Fig. 4a,b and Extended Data Figs. 8 and 9). Overexpression of PanK2 increased free [CoA] to a much lesser extent (about 5% increase in normalized FRET ratio in mitochondria and in cytosol). Knockdown of PanK1 led to an only very small (1.7 ± 0.4%, *P* = 0.004) decrease in normalized FRET ratio in the cytosol and to no significant change in normalized FRET ratio in mitochondria. Knockdown of PanK2 or PanK3 did not lead to significant changes in either compartment. PPCS and PPCDC catalyze the two subsequent reactions in the biosynthetic pathway and both of them are localized to the cytosol^2^. Neither their knockdown nor overexpression led to significant changes in free [CoA] in mitochondria or cytosol, suggesting that the levels of these enzymes are not critical for maintaining CoA homeostasis. COASY catalyzes the last two steps of CoA biosynthesis. Its upregulation increased the FRET ratio of mito-CoA-Snifit^G41S^ by 5.0 ± 1.1% (*P* = 0.0018) but did not significantly affect free [CoA] in the cytosol. This observation in our opinion supports the mitochondrial matrix localization of COASY^14^. The monofunctional DCKAD also catalyzes the last step in the pathway^7^. A change in its expression level, however, did not lead to significant changes in normalized FRET ratio in either cytosol or mitochondria (Fig. 4a,b). PanK was previously proposed to be the rate-controlling enzyme of CoA biosynthesis according to indirect evidence^8,9^. Indeed, using CoA-Snifit, we observed a much stronger impact of PanK overexpression on free cytosolic and mitochondrial [CoA] compared to that of all other members of the biosynthetic pathway, providing direct evidence that cellular [CoA] is particularly sensitive to PanK regulation.

The mitochondrial CoA pool is proposed to be regulated by Nudt8, which hydrolyzes CoA to PPanSH and PAP^18^ (Fig. 1b). In agreement with that hypothesis, depletion of Nudt8 showed a 9.3 ± 1.3% (*P* < 0.0001) increase in normalized FRET ratio in the mitochondria, indicating an increase of mitochondrial free [CoA] (Fig. 4b, Extended Data Fig. 9 and Supplementary Figs. 8 and 9).

Hopantenate (HoPan) is an analog of pantothenate and is used to chemically inhibit CoA biosynthesis^37^ (Fig. 4c). HoPan competes with pantothenate for phosphorylation by PanKs and phosphorylated HoPan inhibits PPCS^37,38^. Incubation of cells overexpressing PanK3 in the presence of HoPan in pantothenate-depleted medium results in a significant drop in free [CoA] in both cytosol and mitochondria (Fig. 4d,e and Supplementary Figs 10 and 11). This drop could be partially compensated by co-incubation with PPanSH, the substrate of the last step of CoA biosynthesis. This observation is consistent with the previous finding that extracellular PPanSH can feed into cellular CoA biosynthesis and can rescue CoA-deprived phenotypes^39^.

### Regulation of PanK in HEK293 cells

PanKs are feedback regulated and thus act as metabolic sensors. Specifically, high concentrations of acyl-CoA lead to increased formation of a catalytically inactive PanK dimer^8,9^. Among the four active human PanK isoforms, biochemical data suggest that PanK2 is most sensitive to feedback inhibition by AcCoA and acyl-CoAs^40^. Also, acyl-carnitine was found to potently activate PanK2 by competitively antagonizing acyl-CoA inhibition^41^. Pantazine 2891 (PZ-2891; Fig. 4c) is an allosteric PanK activator that inhibits the feedback regulation of PanKs by acyl-CoA and keeps the enzyme in an active conformation^36^. Incubation of HEK293 cells with PZ-2891 significantly increased free [CoA] in both cytosol and mitochondria presumably because of the disruption of PanK feedback regulation (Fig. 4f,g). As a control to test for possible interference of our sensor readout with potential changes in ATP concentrations, we examined whether PZ-2891 also affects cytosolic and mitochondrial ATP levels in HEK293 cells using the fluorescent sensor ATeam (Methods; Supplementary Fig. 12). However, PZ-2891 did not induce any obvious ATP changes in either compartment.

Administration of PZ-2891 to cells overexpressing PanK1 and PanK3 further increases the normalized FRET ratio by 20–40%, whereas the overexpression of PanK2 affected free [CoA] to a much smaller extent (< 7.5%, Fig. 4f,g). As PanK2 is localized in the mitochondrial IMS^10^, we speculated that PZ-2891 has to compete with high levels of acyl-CoAs and acyl-carnitine in the IMS and therefore activates PanK2 only partially^42,43^. To test this hypothesis, we removed its N-terminal localization sequence and the resulting truncated PanK2 (PanK2ΔN) showed cytosolic localization^10^. Indeed, expression of PanK2ΔN in cells incubated with PZ-2891 resulted in significant increases in both cytosolic and mitochondrial free [CoA] (Supplementary Fig. 13), supporting our hypothesis that localization of PanK2 to the mitochondrial IMS hinders activation by PZ-2891.

We also investigated whether CoA levels are affected by the activity of ATP citrate lyase (ACLY), which is the primary enzyme responsible for the synthesis of cytosolic AcCoA from CoA^44^. ACLY could affect free [CoA] in two ways: first by the consumption of CoA and secondly by the production of AcCoA, which then inhibits PanK. Indeed, knockdown of ACLY increased the normalized FRET ratio by 7.4 ± 1.0% (*P* < 0.0001) in the cytosol (Fig. 4a and Supplementary Fig. 9), suggesting an increased free [CoA].

### Transport of CoA between cytosolic and mitochondrial pools

**Fig. 5:**
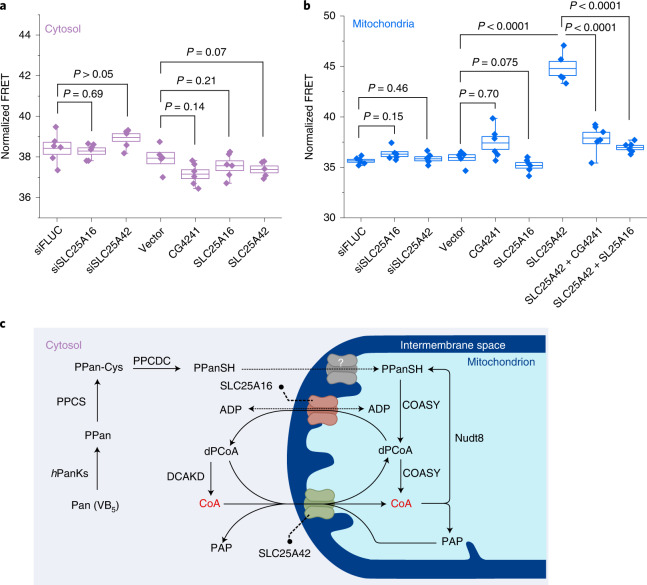
Role of transporters in CoA homeostasis. **a**,**b**, Effect of overexpression or knockdown of selected transporters on cytosolic (**a**) and mitochondrial (**b**) normalized FRET ratio. Cells transfected with non-targeting esiRNA for firefly luciferase (siFLUC) were used as the negative control for gene knockdown. Cells transfected with empty vector were used as the negative control for gene overexpression. The box plots represent the s.e.m. at the lower and upper box limits and the mean as the middle bar. *n* = 5 or 6 FOVs over 4 independent samples with >50 cells per FOV. The whiskers extend to ±1.5× the interquartile range beyond the limits of the boxes, respectively. The precise *n* and *P* values are listed in the Supplementary Data. ***P ≤ 0.001. Two-tailed unpaired *t*-test. **c**, Proposed biosyntheti**c** map of CoA biosynthesis in human cells. Source data

An important question is how CoA, dPCoA and PPanSH are transported in and out of the mitochondrial matrix. In humans, two related solute carrier proteins, SLC25A42 and SLC25A16, also known as Graves disease carrier protein, have been identified as putative CoA and dPCoA transporters^15,16^. Reconstituted SLC25A42 has been shown to transport CoA and dPCoA, but it also showed affinity for ADP and PAP^15^. The substrate specificity of SLC25A16 is unknown at present. An ortholog of SLC25A16 and SLC25A42 is CG4241 from *Drosophila melanogaster*, which was shown to function as a transporter of dPCoA but not of CoA^45^. Overexpression of CG4241 (Extended Data Fig. 8) thus offers the opportunity to specifically increase transport of dPCoA across the mitochondrial membrane of HEK293 cells. To further study the role of SLC25A16, SLC25A42, and CG4241, we measured cytosolic and mitochondrial free [CoA] after performing overexpression of the three transporters or knockdown of SLC25A16 and SLC25A42 in HEK293 cells. Neither overexpression of SLC25A16 and CG4241 nor depletion of SLC25A16 or SLC25A42 induced significant changes in mitochondrial or cytosolic free [CoA] (Fig. 5a,b). By contrast, overexpression of SLC25A42 resulted in an obvious increase (24.6 ± 1.9%, *P* < 0.0001) in mitochondrial free [CoA] but not in cytosolic free [CoA]. One possible explanation for this increase could be the transport of CoA, and possibly dPCoA, into mitochondria through SLC25A42^15^. The increase in mitochondrial free [CoA] was restored to basal levels when SLC25A42-overexpressing cells were co-transfected with SLC25A16 or CG4241 (Fig. 5b). The observation that overexpression of SLC25A16 and CG4241 resulted in similar effects on mitochondrial [CoA] suggests that they have similar substrate specificities, that is SLC25A16 is also specific for dPCoA. The observed decrease in mitochondrial free [CoA] could be rationalized by assuming transport of dPCoA by SLC25A16 and CG4241 out of mitochondria^45^.

### Quantifying free subcellular CoA levels by FLIM

**Extended Data Fig. 10:**
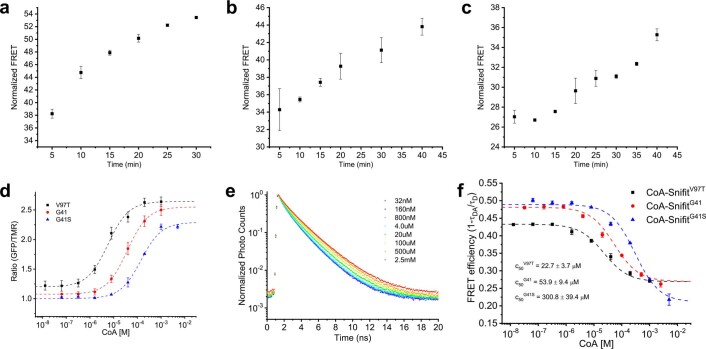
FLIM measurement of CoA in cells. **a-c**. Normalized FRET ratios of cytosolic CoA-Snifits in hemolysin permeabilized HEK293 cells. The cells were treated with 1 μg/mL hemolysin in DPBS for 40 min and then incubated with 20 μM CoA for CoA-Snifits^V97T^ (**a**), 40 μM CoA for CoA-Snifits^G41^ (**b**), and 100 μM CoA for CoA-Snifits^G41S^ (**c**) in DPBS (1 mM Mg^2+^, 1 mM ATP, and 0.2 mg/mL BSA) for 30–45 min. The results showed that the normalized ratio signal gradually increased but did not reach an equilibrium within 30 min, making it difficult to perform calibration titration curves in those permeabilized cells. The data were shown as mean ± s.d., n = 3 field of views (FOVs) over 3 independent samples with > 50 cells per FOV. The normalized FRET ratio values were calculated for each FOV. **d**. The HEK293 cells stably expressing cytosolic sensor proteins of CoA-Snifits were labeled with Halo-MaP-TAZ for 12 h and were lysed by two flash freeze–thaw cycles in liquid nitrogen. The cell lysate was further cleared by centrifugation at 20,000 g and 4 ^o^C for 20 min. The concentration of the sensor was evaluated by measuring the absorbance at 560 nm ((MaP)_560nm_ = 85000 M^−1^cm^−1^). The lysate was diluted in PBS (supplemented with 50 mM HEPES and 0.2 mg/mL BSA) reaching a sensor concentration of 100 nM. The fluorescent titration experiments were performed according to the method described in the main text. Fluorescence intensity ratio (F_510_/F_580_ nm) responses (mean ± s.d., n = 3 independent samples) of the cell lysate of the HEK293 cells containing CoA-Snifit^V97T^ (black), CoA-Snifit^G41^ (red), and CoA-Snifit^G41S^ (blue) as a function of CoA concentration. **e-f**. FLIM measurements of CoA-Snifits upon titration with different concentrations of CoA. **e**. Normalized photon counts of CoA-Snifit^G41^ upon the titration with CoA (32 nM to 2.5 mM) in DPBS (supplemented with 25 mM HEPES, 1 mM Mg^2+^, 1 mM ATP, pH 7.4) as a representative example for the CoA-Snifits (n = 3 independent replicates). **f**. FRET efficiency calculated by fluorescence lifetime responses of CoA-Snifits as a function of CoA concentration (mean ± s.d., n = 3 independent replicates). Source data

A ratiometric sensor such as CoA-Snifit offers the possibility of determining absolute concentrations of free [CoA]. For quantification, fluorescence lifetime imaging microscopy (FLIM) is considered more reliable than ratiometric imaging as it does not depend on the concentration of probe, sample thickness, photo-bleaching and/or excitation intensity^46^. FRET shortens the average fluorescence lifetime of the donor. Hence, the lifetime of the donor of the CoA-Snifit reports on [CoA]. Absolute quantifications of free [CoA] using CoA-Snifits requires a suitable in cellulo calibration curve. However, we were unsuccessful in maintaining defined cytosolic [CoA] in permeabilized cells (Extended Data Fig. 10a–c). We then attempted to measure titration curves of the labeled cytosolic CoA-Snifit in cell lysate of HEK293 cells. The measured *c*_50_ values were increased 1.5–2.7-fold compared with the values obtained for recombinant CoA-Snifits in buffer, whereas the *R*_min_ and *R*_max_ values of the sensors obtained in lysate or buffer were almost identical (<10%; Extended Data Fig. 10d and Supplementary Table 4). The most obvious explanation for the observed differences in *c*_50_ values measured under these different conditions is partial conversion of CoA by CoA-metabolizing enzymes in the lysate. We therefore used calibration curves measured with recombinant CoA-Snifits in buffer (Extended Data Fig. 10e,f) as an approximation to calculate absolute concentrations from FLIM measurements in the cytosol and mitochondria. Specifically, we used CoA-Snifit^G41^ or CoA-Snifit^G41S^ to quantify free [CoA] in the cytosol and mitochondrial matrix of HEK293, U-2 OS, HeLa and HepG2 cells, respectively (Supplementary Table 5). Cytosolic free [CoA] was rather similar in all four cell lines with values ranging from 66 to 85 μM. These values for cytosolic free [CoA] are in the range of those reported for cytosolic CoA levels in heart and liver tissues (50–90 μM)^3^. We found mitochondrial free [CoA] in the four cell lines to be at least threefold higher than cytosolic free [CoA] (around 258–816 μM). The mitochondrial free [CoA] in these cell lines is at least fourfold lower than the reported value of around 3 mM for free mitochondrial CoA of heart and liver tissues^3^. It should also be noted that reported free CoA concentrations refer to both unbound and protein-bound CoA, whereas our sensors measure the unbound free CoA fraction in the cells.

## Discussion

In this work, we introduce, to our knowledge, the first biosensor for the measurement of free cellular [CoA], one of the central cofactors in metabolism. The biosensor is a member of a growing class of biosensors based on the specific labeling of proteins with synthetic fluorescent probes^20^. The sensing mechanism of the CoA-Snifit is based on the competition of a tethered fluorescent ligand with CoA to a binding protein. Tuning the relative affinities of the binding protein for CoA and the tethered ligand allows to rationally shift the sensitivity of the sensor by over two orders of magnitude. This tuning is important for the use of CoA-Snifits in different cellular compartments and also confirms the Snifit design principle. CoA-Snifits possess high selectivity for CoA and are relatively insensitive towards changes in pH. The ratiometric readout allows to compare relative CoA concentrations under different conditions and in different subcellular compartments. Measurement of absolute CoA concentrations through FLIM relied on calibration curves measured in vitro. As our CoA-Snifits are relatively environmentally insensitive, we believe that this is a reasonable assumption.

A future application of CoA-Snifits is in screening for synthetic compounds or genes that affect CoA homeostasis. For example, the failure of the PPan derivative fosmetpantotenate as a replacement therapy for pantothenate kinase-associated neurodegeneration (PKAN) creates the need for alternative molecules to raise intracellular CoA levels in such diseases^47,48^. Screening for transporters involved in CoA homeostasis would be another important application. Proof-of-principle experiments already showed that CoA-Snifits can be analyzed through flow cytometry (Supplementary Fig. 14), which would facilitate such screens.

CoA-Snifits allowed us to gain insights into how cells control CoA homeostasis. Our results confirm previous findings that regulating the activity of PanKs is a key factor in controlling free [CoA] in the cytosol and mitochondria^9^. Our results also suggest that regulating the activity of PanK2 depends on its localization to the mitochondrial IMS, where it is exposed to relatively high levels of acyl-carnitine and acyl-CoAs^41,49^.

There are conflicting reports in the literature concerning the localization of COASY^11–14^. We observed that overexpression of COASY resulted in an increase in free mitochondrial [CoA] but does not affect free [CoA] in the cytosol, supporting that COASY is localized in the mitochondrial matrix^14^.

A key question concerning CoA homeostasis is how CoA and its biosynthetic precursors are transported between the cytosol and mitochondrial matrix^2,3^. Our results confirm that the two transporters SLC25A16 and SLC25A42 are indeed involved in CoA homeostasis. SLC25A42 appears to be involved in the transport of CoA^15^, and possibly also of dPCoA, into mitochondria, as overexpression of SLC25A42 raises mitochondrial free [CoA] but not cytosolic free [CoA]. The similar behavior of SLC25A16 and CG4241, a transporter from *D. melanogaster* specific for dPCoA, furthermore indicates that SLC25A16 is involved in the transport of dPCoA from mitochondria to the cytosol^45^. This hypothesis is supported by the previous findings that (i) SLC25A16 and CG4241 both complement the deletion of the yeast gene LEU5^16^, which was proposed to be a yeast dPCoA transporter^45^ and (ii) the isolated yeast mitochondria do not show any transport activity for CoA^16^.

From our findings and literature data, a cellular map of CoA biosynthesis in mammalian emerges (Fig. 5c), in which the existence of transporters with different specificities for CoA and dPCoA together with the mitochondrial localization of COASY and the cytosolic localization of DCAKD provides the cell with a mechanism to maintain differences in free [CoA] in the cytosol and mitochondria. However, many important open questions concerning CoA homeostasis remain. One important open question is whether specific transporters for PPanSH exist or whether either SLC25A16 or SLC25A42 also transports PPanSH. We have shown that supplying cells with PPanSH under conditions of impaired phosphorylation of pantothenate increases free [CoA] in the cytosol and mitochondria. It has been reported that the total concentration of PPanSH is very low (~4% of total CoA) in cells and is insensitive to PZ-2891 treatments^36^, suggesting rapid transport of PPanSH into mitochondria and efficient conversion to CoA. While it has been suggested that PPanSH passively diffuses into cells^39^, we consider it is more likely that transporters are involved in its cellular uptake and transport into mitochondria. Our CoA-Snifits should enable the search for such transporters.

Finally, we describe the first attempt of a direct determination of free [CoA] in the cytosol and mitochondria of living cells. The measured values for free cytosolic [CoA] are very similar for all analyzed cell lines and are also in agreement with literature values obtained for total cytosolic CoA levels in other cell types^3^. By contrast, our values for free mitochondrial [CoA] differ up to threefold among the different cell lines and are a least fourfold lower than CoA levels determined for mitochondria isolated from liver or heart tissue, which also took into account protein-bound CoA^22–25^. A possible explanation for the much larger variation seen for mitochondrial [CoA] among the different cell lines and tissues is that they depend to different degrees on CoA-dependent oxidative phosphorylation for ATP production^50^. Our sensors provide a tool to investigate such central questions on cellular metabolism further. Finally, expanding the use of CoA-Snifits to other compartments, such as the peroxisome, in which CoA plays a key role in lipid metabolism, would also be of high interest.

In summary, we describe a biosensor for measuring free cytosolic and mitochondrial [CoA] in living cells and apply the sensor to answer central questions concerning CoA homeostasis. We expect that CoA-Snifits will become an important tool for studying the role of CoA in metabolism.

## Methods

Detailed procedures for the synthesis of the probes are given in the Supplementary Note.

### Molecular biology

A pET51b(+) vector (Novagen) was used for protein production in *E. coli*. Proteins were N-terminally tagged with Strep-tag and C-terminally tagged with His_x10_, respectively. A pcDNA5/FRT/TO vector (Thermo Fisher Scientific) was used for generating HEK293 cells that stably express sensor proteins.

The plasmids encoding the genes of *PanK1*, *PanK2*, *PanK3*, *COASY*, *SLC25A16*, and *SLC25A42* were obtained from Addgene plasmid repository (Supplementary Data). The genes of *PPCS*, *PPCDC*, *DCAKD*, *ACLY*, and *NUDT8* were amplified from cDNA, which was prepared by QuantiTect Reverse Transcription Kit (Qiagen) using total RNA of HEK293 cells. The genes of *E. coli PANK* were synthesized by Eurofins Genomics. These genes were subsequently cloned into PET51b(+) or pcDNA5/FRT vector by Gibson Assembly.

### Protein production and purification

The *ec*PanK variants, human PanKs (*h*PanK1, *h*PanK2 and *h*PanK3), and sensor proteins were expressed in *E. coli* strain BL21(DE3). The bacterial culture was incubated at 37 °C to reach an optical density at 600 nm (OD_600_) of 0.8. The protein expression was induced by adding 1.0 mM isopropyl β-d-thiogalactopyranoside (IPTG) to the culture. The culture was then cooled to 16 °C and incubated overnight while shaking at 220 r.p.m. After 20 h, the bacteria were harvested by centrifugation at 4,000*g* for 10 min and lysed by sonication in the presence of 1 mg ml^−1^ lysozyme and 1 mM phenylmethylsulfonyl fluoride (PMSF). The cell lysate was cleared by centrifugation at 20,000*g* and 4 °C for 20 min. All proteins were purified using Ni-NTA affinity chromatography (Qiagen), which was followed by Strep-Tactin purification (IBA Lifesciences), according to the manufacturer’s protocol. The protein concentration was determined by measuring the absorbance at 485 nm (*ε*_sfGFP_ = 83,300 M^−1^cm^−1^). The purified proteins were diluted in phosphate-buffered saline (PBS) (10 mM Na_2_HPO_4_, 1.8 mM KH_2_PO_4_, 137 mM NaCl, 2.7 mM KCl, pH 7.4) to 50 μM, aliquoted, flash frozen in liquid nitrogen and stored at −80 °C until use.

### Engineering of *ec*PanK

In a fluorescence polarization competition assay with TMR-TAZ, *ec*PanK only exhibited a 6.6-fold higher affinity for CoA over acetyl-CoA (AcCoA) (Extended Data Fig. 1e) and we thus attempted to increase the specificity of *ec*PanK for CoA over AcCoA. The sulfhydryl moiety (-SH) can interact favorably with aromatic rings through thiol-aromatic interactions^51^, and we therefore mutated L277 in the binding pocket of *ec*PanK to W (Fig. 2b). We furthermore introduced the mutation F252Y to sterically discriminate against AcCoA. The resulting double mutant L277W, F252Y displayed a 21-fold higher affinity for CoA over AcCoA (Supplementary Table 1). Furthermore, the catalytic activity of *ec*PanK was abolished by implementing the D127A mutation in the active site^52^ (Extended Data Fig. 2g). The triple mutant *ec*PanK^D127A,F252Y,L277W^ exhibited a *K*_d_ value of 21.6 ± 3.7 μM for TMR-TAZ whereby CoA competed with TMR-TAZ for binding (Supplementary Table. 1). For the circular permutation of *ec*PanK, we introduced new termini near the substrate binding site at residues D213 and P214 and connected the original termini by a flexible linker GSGGTG, resulting in ^cp^*ec*PanK^D127A,F252Y,L277W^, which was used for the generation of CoA-Snifit^G41^. The point mutations were made using the Q5 site directed mutagenesis kit according to the manufacturer’s protocol.

### HaloTag labeling in vitro

The sensor proteins (1 μM) were labeled in PBS spiking labeling substrate 8 iterative times every 15 min to reach a final concentration of 4 μM. After that, the mixture was incubated at room temperature for another 1 h, the excess of probe was washed out (two cycles) using a centrifugal filter device (Microcon YM-50, Millipore) using PBS. The labeled sensors were stored at 4 °C until further use.

### In vitro PanK activity assays

The activity of wild-type *ec*PanK^WT^, the dead mutant *ec*PanK^D127A^ and *h*PanKs was determined by performing absorbance kinetics on a microplate reader (Spark 20 M, Tecan). The production of ADP was coupled to the consumption of NADH, by using pyruvate kinase (PK, Sigma-Aldrich) and lactate dehydrogenase (LDH, Sigma-Aldrich). The reaction mix (200 μl) contained 50 mM Tris-HCl, 10 mM MgCl_2_, 20 mM KCl, 1.5 mM ATP, 0.5 mM NADH, 0.5 mM phosphoenolpyruvate, 3 units of PK, 3 units of LDH, 0.5 μM of PanK, pH 7.6. The reaction was initiated by the addition of 200 μM pantothenate and was monitored by following the decrease in absorption at 340 nm at 25 °C.

### Sensor titration

One hundred nanomolar sensors were titrated in PBS, supplemented with 50 mM HEPES and 0.5 mg ml^−1^ bovine serum albumin (BSA) with defined concentrations of CoA in a final volume of 50 μl in black flat-bottom non-binding 96-well plates (FALCON). After 5 min incubation at 25 °C, fluorescence emission spectra were recorded on a microplate reader (Spark 20M, Tecan). The sensor was excited at 450 nm (bandwidth 10 nm) and the emission spectra were recorded from 480 to 650 nm (bandwidth 10 nm) with a step size of 2 nm. FRET ratios (sfGFP/MaP) were calculated from the emission intensity of sfGFP (510 nm) and MaP dye (580 nm), and further plotted against the CoA concentration. To obtain the concentration of half-maximal ratio change, *c*_50_, the equation (1) was fitted to the data:

1 $$R = R_{\mathrm{min}} + \frac{{R_{\mathrm{max}} - R_{\mathrm{min}}}}{{1 + \frac{{c_{50}}}{{[\mathrm{CoA}]}}}}$$

Where *R* is the experimental FRET ratio, [CoA] is the concentration of free CoA, and *R*_min_ and *R*_max_ are the FRET ratio in absence and at saturation of CoA, respectively. Fits were performed using OriginPro 2021 with free fit parameters *c*_50_, *R*_min_, and *R*_max_. Dynamic range (maximum ratio change of emission intensity) was calculated as Δ*R* = *R*_max_/*R*_min_.

### Fluorescence polarization

Fifty nanomolar TMR-TAZ was incubated with varying amounts of PanK proteins for 5 min at room temperature in PBS supplemented with 0.5 mg ml^−1^ BSA. Assays were performed in non-binding black 96-well plates (Corning) with a final volume of 50 μl and were measured on a microplate reader (Spark 20M, Tecan) with excitation at 520 nm (bandwidth 20 nm) and emission 600 nm (bandwidth 20 nm). Fluorescence polarization (FP) was calculated according to equation (2).

2 $$\mathrm{FP} = \frac{{I_\parallel - I_ \bot G}}{{I_\parallel + I_ \bot G}}$$

Where FP is the fluorescence polarization, I_∥_ is the fluorescence intensity parallel to the excitation light polarization, I_⊥_ is the fluorescence intensity perpendicular to the excitation light polarization, and *G* is the grating factor (*G* = I_∥_/I_⊥_). Three independent titrations were performed for each protein variant. The equation (3) was fitted to the data using OriginPro 2021.

3 $$\begin{array}{l}\mathrm{FP} = \mathrm{FP}_{0} + \left( {\mathrm{{FP}_{s} - {FP}_{0}}} \right)\\ \times \frac{{\left( {[L] + K_\mathrm{d} + \left[ {\mathrm{protein}} \right]} \right) - \left. {\sqrt {\left( {\left[ L \right] + \left[ {\mathrm{protein}} \right] + K_\mathrm{d}} \right)^2 - 4\left[ L \right]\left[ {\mathrm{protein}} \right]} } \right)}}{{2\left[ L \right]}}\end{array}$$

Where FP_0_ is the fluorescence polarization of the free fluorophore, FP_s_ is the fluorescence polarization of the fluorophore bound with protein, *K*_d_ is the dissociation constant, [protein] is the concentration of *ec*PanK proteins, and [*L*] is the concentration of TMR-TAZ, which was 50 nM in this case.

The binding affinity (*c*_50_) of different *ec*PanKs for CoA and AcCoA were determined by a fluorescence polarization competition assay against TMR-TAZ. 50 nM TMR-TAZ and 20 μM *ec*PanKs, except *ec*PanK^D127A,F252Y,L277W^ (100 μM), were titrated against CoA or AcCoA concentrations ranging from 64 nM to 5 mM in PBS supplemented with 0.5 mg ml^−1^ BSA and 50 mM HEPES. Assays were performed in non-binding black 96-well plates with a final volume of 50 μl with excitation at 520 nm (bandwidth 20 nm) and emission 600 nm (bandwidth 20 nm). Obtained FP values were averaged and fitted to equation (4) to estimate the *c*_50_ values using OriginPro 2021.

4 $$\mathrm{FP} = \mathrm{FP}_{0} + \frac{{\mathrm{{FP}_{s} - {FP}_{0}}}}{{1 + \frac{{c_{50}}}{{[L]}}}}$$

Where FP_0_ is the fluorescence polarization of the fluorophore with *ec*PanKs in the absence of CoA or AcCoA (bound fluorophore), FP_s_ is the fluorescence polarization of the fluorophore in the presence of CoA or AcCoA (free fluorophore) c_50_ is the half-maximal effective concentration, and [*L*] is the concentration of CoA or AcCoA.

### Cell culture

HEK293 cells, which refers to the Flp-In-T-REx-293 cell lines (Thermo Fisher Scientific) in this study, HeLa, and U-2 OS cell lines were grown in high-glucose Dulbecco’s modified Eagle’s medium (DMEM) with GlutaMAX-I, 1 mM pyruvate (Gibco) supplemented with 10% (v/v) heat-inactivated fetal bovine serum (FBS) (Gibco), and HepG2 cell line was grown in RPMI-1640 medium (Gibco) supplemented with 10% (v/v) heat-inactivated FBS. All cells were grown at 37 °C, 5% CO_2_. Cells stably expressing the sensor protein were generated according to the standard protocol from Thermo Fisher Scientific. In brief, Flp-In-T-REx-293 cells were co-transfected with pOG44 and pcDNA5/FRT/TO plasmid encoding Flp-recombinase and sensor proteins, respectively, followed by selection with hygromycin B (100 μg ml^−1^). To induce protein expression, cells expressing cytosolic sensors were incubated in the presence of 400 ng ml^−1^ doxycycline for 12 h and cells expressing mitochondrial sensors were incubated in the presence of 4 ng ml^−1^ doxycycline for 12 h.

### Subcellular colocalization

The subcellular localization of cytosolic and mitochondrial sensor was determined in living cells by staining the cells with commercial fluorescent probes (see below). The localization of PanKs, Nudt8 and CG4241 was determined by transiently transfection of C-terminal GFP-tag fusion proteins (Extended Data Fig. 8). We assume that the localization of PanKs, SLC25A16, SLC25A42, and CG4241 is not affected by their overexpression, as we could not verify their localization through immunostaining with commercial antibodies.

For the cytosolic and mitochondrial sensors, cells stably expressing the sensors were seeded in a glass-bottom 96-well cell imaging plate and the sensor expression was induced in full growth medium at 37 °C, 5% CO_2_ for 12 h with 400 and 4 ng ml^−1^ doxycycline, respectively. For PanK1, PanK2, PanK3, and CG4241, HEK293 transiently transfected with PanK1–GFP (catalytic core of PanK1α), PanK2–GFP, PanK3–GFP, and CG4241–GFP fusion proteins were allowed to grow for 24 h after transfection.

The cells were labeled in full growth medium with 100 nM MitoTracker Red CMXRos and 1 μg ml^−1^ Hoechst 33342 at 37 °C, 5% CO_2_ for 1 h. Excess of dye was removed by washing twice with HBSS supplemented with 0.2 mg ml^−1^ BSA. The medium was exchanged with HBSS before imaging. The images were analyzed with ImageJ^53^.

### Immunostaining

The HEK293 cells transiently transfected with pcDNA5/FRT plasmid encoding NUDT8 gene were grown on a glass-bottom 96-well cell imaging plate for 24 h. After staining with 100 nM MitoTracker Red CMXRos for 1 h, the cells were fixed with 4% PFA in PBS for 15 min at 25 °C, followed by permeabilization with 0.3% Triton X-100 in PBS. The cells were washed with PBS and blocked with 3% BSA in PBS for 1 h at 25 °C and then incubated for 12 h at 4 °C with 1% BSA in PBS containing 2 μg ml^−1^ rabbit primary polyclonal antibody Nudt8 (PA5-59493, 1:50, Thermo Fisher Scientific), followed by 1 h incubation with Alexa Fluor 647 goat anti-rabbit IgG antibody (A32728, 1:1,000, Invitrogen) and 1.0 μg ml^−1^ Hoechst 33342 at 25 °C. The cells were washed three times with PBS before imaging.

### Capillary immunoblotting analysis

Immunoblotting was performed using the system Wes (ProteinSimple) according to the user manual. The cells were extracted using Cell Lysis Reagent (Sigma-Aldrich). Total lysate was mixed with a master mix (ProteinSimple) to a final concentration of 1× sample buffer, 1× fluorescent molecular weight marker and 40 mM dithiothreitol, then heated at 95 °C for 5 min. The samples, blocking reagent, primary antibodies, horse-radish-peroxidase-conjugated secondary antibody, chemiluminescent substrate, separation and stacking matrices were also dispensed to designated wells in 25 well plates. After plate loading, separation electrophoresis and immunodetection steps took place in the capillary system and were fully automated. Capillary immunoblotting analysis was carried out at room temperature, and instrument default settings were used. Capillaries were first filled with separation matrix followed by stacking matrix, and about 40 nl sample loading. During electrophoresis, proteins were separated on the basis of molecular weight through the stacking and separation matrices at 250 V for 40 min and then immobilized on the capillary wall using proprietary photo-activated capture chemistry. The matrices were then washed out. Capillaries were next incubated with a blocking reagent for 15 min, and target proteins were immunoprobed with primary antibodies to GAPDH (NB300-322, 1:1,000, Novus Biologicals), Nudt8 (PA5-59493, 1:50, Thermo Fisher Scientific), PanK2 (PA5-52563, 1:20, Thermo Fisher Scientific), COASY (WH0080347M1-100UG, 1:500, Sigma-Aldrich), and ACLY (PA5-29497, 1:100, Thermo Fisher Scientific), followed by anti-rabbit detection module (DM-001, ProteinSimple) or anti-mouse detection module (DM-002, ProteinSimple) secondary antibodies, addition of chemiluminescence detection mixture (ProteinSimple) and imaging. The images were analyzed by Compass for SW version 4.0.0 (ProteinSimple).

### Labeling sensor protein in mammalian cells

After induction with doxycycline, the cells stably expressing sensor proteins were labeled with 1 μM fluorescent probe (Halo-MaP-TAZ, 1 mM stock in dimethy sulfoxide (DMSO), 1000×) in fresh pre-warmed full growth medium supplemented with 10% FBS at 37 °C, 5% CO_2_ for 12 h. Then, the cells were washed once with HBSS supplemented with 0.2 mg ml^−1^ BSA and incubated in this buffer before imaging.

### Confocal microscopy

Images of HEK293 cells labeled with probes were taken using a Leica TCP SP8 confocal microscope equipped with a 40× plan Apochromat 1.4 numerical-aperture water-immersion objective lens. As excitation source, the white light laser was set to 480 nm with 80 MHz pulse frequency for GFP excitation and was set to 535 nm with 80 MHz pulse frequency for MaP dye excitation. Fluorescence signal was collected at 490–540 nm for GFP and at 560–620 nm for MaP, respectively. The scanning parameters were set to 1.5× zoom, scan speed 200 Hz, pixel size 0.379 μm, image format 512 × 512 pixels, pinhole 77 μm. Unlabeled cells were used as the donor-only sample. The HEK293 cells stably expressing HaloTag-(EAAAK)_5_-*ec*PanK fusion, on which sfGFP was removed, were labeled with Halo-MaP-TAZ and used as the acceptor-only sample. Then the FRET images of the full field of view (FOV) were processed using the PixFRET plugin of ImageJ^54^. The net FRET is calculated according to equation (5):

5 $$\mathrm{netFRET} = \frac{{I_{\mathrm{FRET}} - I_{\mathrm{donor}} \times \mathrm{BT}_{\rm{donor}} - I_{\mathrm{acceptor}} \times \mathrm{BT}_{\rm{acceptor}}}}{{\sqrt {I_{\mathrm{donor}} \times I_{\mathrm{acceptor}}} }}$$

Where *I*_FRET_, *I*_donor_, and *I*_acceptor_ are intensities in the region of interest (ROI) under FRET, sfGFP, and MaP microscopy settings, respectively. BT_donor_ is a factor of the percentage of sfGFP bleedthrough, and BT_acceptor_ is a factor of the percentage of MaP bleedthrough under the FRET microscopy settings. The values for the bleedthrough were determined by analyzing images of donor-only or acceptor-only samples and quantifying the relative intensity ratio under the FRET/donor or FRET/acceptor settings. The ratio was reported as normalized FRET ratio by comparing the net FRET ratio of Halo-MaP-TAZ-labeled cells and Halo-MaP-Me-labeled cells, which do not show ratiometric response to CoA concentrations, according to equation (6).

6 $${\mathrm{normalized}}\,{\mathrm{FRET}} = \frac{{\mathrm{netFRET}_{\rm{Halo-MaP-Me}}}}{{\mathrm{netFRET}_{\rm{Halo-MaP-TAZ}}}} \times 100{{{\mathrm{\% }}}}$$

The ROI was defined for each FOV by thresholding the fluorescence intensities at the GFP channel to identify more than 50 cells per FOV. The normalized FRET values for the ROIs (cells) were extracted and used for further calculations and statistical analysis.

### Intracellular labeling efficiency

The complete labeling of intracellular protein was determined by in-gel fluorescence. HEK293 cells expressing the cytosolic Halo-SNAP fusion protein were labeled with 1 μM Halo-MaP-TAZ in full growth medium for 2, 4, 6, 8, 10, and 12 h. Cells were washed three times with PBS to remove the excess of dyes and were resuspended in PBS supplemented with protease inhibitor cocktail (complete-EDTA-free, Roche). Cells were lysed by two flash freeze–thaw cycles and 1 μM Halo-Alexa488 (Promega) and 1 μM BG-SiR^55^ was added to quantify the unlabeled fraction of HaloTag. After 30 min incubation, the cell extract was centrifuged for 10 min at 20,000*g* and the clarified lysate was collected in new tubes and kept on ice. The controls for quantitative labeling were prepared by dually labeling the recombinant sensor with Halo-MaP-TAZ/BG-SiR or Halo-Alexa488/BG-SiR. The different samples were resolved by SDS-PAGE. In-gel fluorescence of Alexa488 (Cy2 channel), MaP (Cy3 channel) and SiR (Cy5 channel) was measured on an Amersham Typhoon 5 Biomolecular Imager (GE Healthcare Bio-Sciences Corp). For quantification, the integrated values of background-corrected band intensities were measured with ImageJ. Labeling efficiency (LE) determination was performed according to equation (7):

7 $$\mathrm{LE}\left( {{{\mathrm{\% }}}} \right) = 100 \times \left( {1 - \frac{{S_{\mathrm{Alexa488}}/S_{\mathrm{SiR}}}}{{C_{\mathrm{Alexa488}}/C_{\mathrm{SiR}}}}} \right)$$

Where *S* corresponds to the sample fluorescence intensity of intracellularly labeled protein at the defined collection window (Cy2 and Cy5 channels) and *C* corresponds to the control fluorescence intensity of purified and labeled protein at the defined collection window.

Complete labeling of intracellular sensor protein was determined by ratiometric imaging in living cells. HEK293 cells expressing the subcellular sensor proteins were labeled with 1 μM Halo-MaP-TAZ in full growth medium for 2, 4, 6, 8, 10, and 12 h. At each time point, the cells were washed with HBSS (0.2 mg ml^−1^ BSA) once and incubated in this buffer for imaging. The results were presented in Extended Data Fig. 7 and Supplementary Figs 3 and 4.

### Protein overexpression in HEK293 cells

Cells were transfected with plasmids using Lipofectamine 3000 (Thermo Fisher Scientific). After 12 h, the medium was changed to fresh one and the expression of sensor protein was induced by treatment with doxycycline at 37 °C, 5% CO_2_ for 12 h. Subsequently, the cells were labeled for another 12 h. Cells transfected with empty vector were used as a negative control.

### Knockdown of gene by esiRNA

HEK293 cells were reversely transfected with endoribonuclease-prepared short interfering RNA, (esiRNA, Sigma-Aldrich) for the gene of interest or non-targeting esiRNA for firefly luciferase (FLUC) using Lipofectamine RNAiMAX (Thermo Fisher Scientific). 0.3 μl of esiRNA and 0.3 μl of Lipofectamine RNAiMAX reagent were mixed in 10 μl Opti-MEM medium, incubated for 10 min and transferred into 96-well plate. One hundred microliters of the prepared cells (3 × 10^5^ cells per milliliter) was added. After 42 h incubation, the medium was changed to fresh one and the sensor expression was induced by treatment with doxycycline at 37 °C, 5% CO_2_ for 12 h. Subsequently, the cells were labeled for another 12 h. The cells transfected with non-targeting siFLUC were used as a negative control.

### RNA extraction and real-time PCR

Total RNA was isolated from the cells (80–90% confluence) with the RNeasy Mini Kit (QIAGEN). RNA quantity was measured with the Nanodrop (Nanodrop Technologies). RNA was directly used in real-time PCR with the one step SYBR Green Quantitative RT-PCR Kit (Sigma-Aldrich) system. Human glyceraldehyde-3-phosphate dehydrogenase (GAPDH) was used as a control. All the real-time values were compared using the *C*_T_ method, where the amount of cDNA (gene overexpression or knockdown) was normalized to the housekeeping gene GAPDH (Δ*C*_T_) before being compared with the amount of cDNA without treatment (ΔΔ*C*_T_), which was set as the calibrator at 1.0 (ref. ^56^). The data represents an average of three independent replicates.

### Treatments of HEK293 cells with HoPan, PPanSH, and PZ-2891

HEK293 cells stably expressing sensor proteins were transfected with the pcDNA5/FRT plasmid encoding *PANK3* gene using Lipofectamine 3000. After 12 h, the cells were incubated in customized vitamin B_5_-free DMEM (Cell Culture Technologies) supplemented with 1/100 dilution of GlutaMAX-I (Gibco), 1 mM pyruvate (Gibco), 10% (v/v) heat-inactivated FBS (Gibco) with doxycycline for induction for 12 h. At the same time, HoPan (Toronto Research Chemicals) was added to the medium to a final concentration of 400 μM, in either presence or absence of 100 μM PPanSH (Chiralix) for 12 h. Subsequently, the cells were labeled for another 12 h with the indicated compounds.

For the PZ-2891 (Selleckchem) treatment, HEK293 cells stably expressing sensor proteins were treated with 1.0 μM PZ-2891 (2 mM stock in DMSO, 2,000×) in normal growth medium for 12 h with doxycycline for induction. Subsequently, the cells were labeled for another 12 h in the presence of 1.0 μM PZ-2891.

### Flow cytometry measurements

To measure intracellular [ATP] changes, the ATeam sensors were used^57^. ATeam sensors were transiently expressed in HEK293 cells either localized to the cytosol or the inner membrane of mitochondria. The treatment with 1.0 μM PZ-2891 was performed for 12 h during the transfection of the sensors. Subsequently the medium was exchanged and the cells were treated with 1.0 μM PZ-2891 for additional 12 h. Then the cells were washed and resuspended in PBS containing 2% FBS (FACS buffer). The 10 mM 2-DG treatment was performed 24 h after transfection whereby the cells were washed with growth medium without glucose for 30 min before the treatment. The cells were resuspended in 10 mM 2-DG prepared in FACS buffer and incubated for 30 min before analysis. The experiments were measured at the BD LSRFortessa X-20 Flow Cytometer (Becton, Dickinson and Company) using the software BD FACSDiva. For each replicate 8,000 events were analyzed. The following settings were used to record the donor, FRET and acceptor fluorescence: BV421 (excitation 405 nm; emission 450/50 nm) for CFP channel, BV510 (excitation 405nm; emission 525/50) for FRET channel and FITC (excitation 488 nm; emission 530/30 nm) for YFP. Gating strategy involved the removal of dead cells and debris (SSC-A versus FCS-A) and selection of the cell population expressing the sensors (CFP versus YFP). The gated populations in the different conditions were analyzed by determining the mean of their FRET/CFP ratio. The final results are presented as violin plots from two independent biological experiments (Supplementary Fig. 12).

To measure intracellular [CoA] changes, the cytosolic CoA-Snifit^V97T^ and mitochondrial CoA-Snifit^G41S^ were used. HEK293 cells stably expressing sensor protein were plated in 12-well plates and cultured in full growth medium at 37 °C, 5% CO_2_. For PanK3-overexpression, the cells were transfected with a plasmid encoding *PANK3* gene and the empty plasmid was used as a negative control. For the PZ-2891, HoPan, or PPanSH treatments, the cells were directly incubated with the 1.0 μM PZ-2891, 400 μM HoPan, or 100 μM PPanSH, respectively. Then, the cells were washed once with FACS buffer and were resuspended in this buffer. Ten thousand cells were analyzed on a FACSMelody Cell Sorter (BD Biosciences). The following settings were used to record the donor, FRET and acceptor fluorescence: FITC (excitation 488 nm; emission 527/32 nm) for GFP channel, PerCP (excitation 488 nm; emission 700/54 nm) for FRET channel and PE-Cy5 (excitation 561 nm; emission 697/58 nm) for MaP channel. HEK293 cells were used as blank control. The data was analyzed in FlowJo software. Gating strategy involved the removal of dead cells and debris (SSC-A versus FCS-A) and selection of the labeled cell population (GFP versus MaP). The gated populations in the different conditions were analyzed by determining the mean of their GFP/MaP ratio. The final results are presented as violin plots from three independent biological experiments (Supplementary Fig. 14).

### Cell permeabilization test

To quantify the free CoA concentration in cells, we attempted to use fluorescence ratiometry as described by Cambronne et. al.^58^. However, we found that treating the cells with low concentrations of digitonin led to low efficiency of permeabilization for the cells, whereas higher concentrations led to obvious cell death and leakage of the sensors out of the cells. We then turned to another permeabilizing agent, hemolysin, which can permeabilize the cells with 1–2 nm pores to permit rapid flux of ions and nucleotides, but not of proteins^59^. We then treated the cells stably expressing the cytosolic sensors with 1 μg ml^−1^ hemolysin for 40 min, and then equilibrated the cells with certain concentrations of CoA in DPBS buffer. The results showed that the normalized ratio was not stable over 30 min (Extended Data Fig. 10a–c), indicating that it is difficult to maintain the free cytosolic CoA concentration at a defined value.

### Live-cell quantification of CoA by FLIM

HEK293 cells stably expressing sensor protein, HeLa, U-2 OS, and HepG2 cells were plated in tissue-culture-treated 96-well imaging plates and cultured in full growth medium at 37 °C, 5% CO_2_. HEK293 cells were induced with doxycycline for 12 h. HeLa, U-2 OS, and HepG2 cells were transiently transfected with the pcDNA5/FRT plasmids encoding the cytosolic apo-CoA-Snifit^G41^ or mitochondrial apo-CoA-Snifit^G41S^ for 24 h. The sensor constructs were labeled with Halo-MaP-TAZ for 12 h in full growth medium. The cells were washed once with HBSS (0.2 mg ml^−1^ BSA) before imaging. Fluorescence lifetime measurements were performed on Leica TCS SP8 confocal microscope equipped with a 40× water-immersion objective and a TCSPC module. As excitation source, the white light laser was set to 480 nm with 40 MHz pulse frequency collecting 1,000 photons per pixel. The FRET donor emission was measured on a hybrid photodetector for single-molecule detection with a detection window of 490–540 nm. The images were typically acquired with 512 × 512 pixels, pixel size 0.379 μm, scan speed 400 Hz. All measurements were performed at 37 °C, 5% CO_2_. Data acquisition and analysis were performed using Leica Application Suite X (LAS X). The fluorescence decays of individual cells were extracted by ROIs (sum of the photons of all the pixels of a ROI, typically with 10^6^ photon counts, total cells > 50) and were fitted using a triexponential decay models (*n*-exponential reconvolution, *χ*^2^ < 1.2).

To set up the calibration curves for CoA concentration, CoA-Snifit^V97T^, CoA-Snifit^G41^, and CoA-Snifit^G41S^ were diluted in PBS (25 mM HEPES, 1 mM Mg^2+^, 1 mM ATP, pH 7.4 for CoA-Snifit^V97T^ and CoA-Snifit^G41^; pH 8.0 for CoA-Snifit^G41S^) with a concentration of 200 nM. The fluorescence lifetime was measured in the presence of increasing concentration of CoA and analyzed using LAS X FLIM/FCS (v.3.5.6). An example of fluorescence decays and fitting results were presented in Extended Data Fig. 10e. The amplitude weighted average lifetimes *τ* were used to calculate the FRET efficiencies according to Equations (8) and (9).

8 $$\tau = \frac{{{\sum} {\alpha _i\tau _i} }}{{\alpha _i}}$$

9 $$E = 1 - \frac{{\tau _{\mathrm{FRET}}}}{{\tau _D}}$$

10 $$\left[ {\mathrm{CoA}} \right] = c_{50}\frac{{E - E_{\mathrm{min}}}}{{E_{\mathrm{max}} - E}}$$

*τ*_FRET_ and *τ*_D_ represent the amplitude weighted average lifetimes for the FRET and donor-only sample (unlabeled sensor protein). The lifetime was measured and reported in Supplementary Table 5, respectively. [CoA] was quantified using equation (10), where *E*, *E*_min_, and *E*_max_ correspond to the FRET efficiency of the sensor in situ prior treatment (basal state), in the absence and presence of CoA. *c*_50_ is the CoA concentration corresponding to half of the maximum sensor response determined from in vitro titrations at 37 °C.

### Liquid chromatography–tandem mass spectrometry measurements for total CoA in cells

The cells were incubated and treated according to the protocols as described above. A total of 2–8 × 10^6^ cells were collected by centrifugation. The cell pellets were lysed by thorough mixing with 200 μl 80% ethanol, which was pre-mixed with 2.5 μM internal standard (CoA-MA, Supplementary Scheme 3). The mixture was vortexed for 5 min and subsequently, the cell debris and protein aggregates were separated by centrifugation at 20,000*g* for 10 min at 4 °C. The supernatant was diluted 50 times in a 10 mM HCOONH_4_ buffer (pH 6.8) for analysis by mass spectrometry.

The analysis was performed using a QT6500+ mass spectrometer from Sciex hyphenated to a Nexera X2 UHPLC from Shimadzu. The instruments were controlled using the Sciex Analyst 1.7 (HotFix 3) software. Data analysis was performed using Sciex MultiQuant 3.0.2 software.

Initially, the analysis of CoA suffered from the lack of a commercially available stable-isotope-labeled internal standard (IS) and the compound instability. Under acidic conditions a drastic CoA signal loss over time was observed, whereas tandem mass spectrometry experiments indicated the formation of dephospho-CoA as a major degeneration product. The degenerative processes were slowed down, resulting in an approximate signal loss of 20% within 8 h, if the samples were dissolved in neutral or slightly alkaline HCOONH_4_ buffer (15 mM HCOONH_4_, pH 7.95), permanently cooled (0–4 °C) and separated using chromatography on the basis of an aqueous phase featuring 15 mM HCOONH_4_ (pH 7.95). In this context, it is worth mentioning that we also found that CoA is temperature labile, resulting in decreased CoA signals with increasing mass spectrometry source temperature. Owing to the compound instability and possible day-to-day variations in the workup procedure, usage of an internal standard was considered as mandatory. Lacking a stable-isotope-labeled CoA analog, a CoA maleimide derivative, CoA-MA, was prepared. The maleimide was incubated in 100 μM GSH to ensure its redox stability. No signal loss was detected within 4 h (data not shown). Furthermore, the chromatographic behavior of the IS compound was evaluated in comparison to CoA. The maleimide featured a retention time of 2.27 min versus a retention time of 2.26 min of CoA, indicating that the IS might also be able to correct electrospray ionization suppression effects.

Freshly prepared samples (10 μl), as described above, were injected on an Acquity UPLC HSS T3 (Waters, 2.1 × 50 mm, 1.8 μm) column. The column temperature was set to 20 °C. The analytes were eluted using a 0.5 ml min^−1^ flow of an aqueous 10 mM HCOONH_4_ solution (pH 7.95) and acetonitrile. After sample injection, a 2-min isocratic flow of 98% of the aqueous phase was applied, followed by a 2–95% organic phase gradient in 3 min. Subsequently, the column was washed using 95% acetonitrile and re-equilibrated. For CoA ionization and fragmentation under the described high-flow conditions the following ion source parameters were chosen: curtain gas 40 p.s.i., collision gas ‘medium’, ionization voltage 5,500 V, temperature 450 °C, heater gas 50 p.s.i. and nebulizer gas 80 p.s.i. Tandem mass spectrometry analysis was performed in the multiple reaction monitoring mode. The explicit multiple reaction monitoring transitions were listed in Supplementary Table 6. Samples and calibrators featuring concentration levels at 1 (LOQ), 2.5, 5, 25, 50, and 100 ng ml^−1^ were run in duplicates. The 10 ng ml^−1^ calibrator was run four times for statistical evaluation, resulting in coefficients of variation with values <5%. At least five consecutive calibrators were either fitted linear or quadratic with a weighting of 1/*x*, resulting in the best possible fit in the concentration range of the respective samples.

### Statistics

Data for in vitro titrations were from three independent replicates and shown as the mean ± s.d. Cell imaging experiments were performed in four independently treated samples (imaging dishes). The normalized FRET ratio was calculated from *n* = 4, 5, or 6 FOVs for each sample and was used for statistical analysis, which was performed using Microsoft Excel 2016. *P* values were calculated using two-tailed Student’s *t*-test. The precise numbers of FOVs (*n*) and *P* values for the figures containing imaging data are listed in the Supplementary Data. For all of the imaging experiments, with the exception of the one comprising PanK2ΔN, the entire procedure was performed in three biological replicates repeated three times. Significance was evaluated for each of these three replicates individually as described above. Only if all biological replicates showed significant differences relative to an appropriate control (*P* < 0.05), the experimental result was considered as significant. One arbitrarily chosen replicate was used for preparation of the figures in the manuscript. The results of all biological replicates are shown in Supplementary Figs. 15 and 16.

### Reporting summary

Further information on research design is available in the Nature Research Reporting Summary linked to this article.

## Online content

Any methods, additional references, Nature Research reporting summaries, source data, extended data, supplementary information, acknowledgements, peer review information; details of author contributions and competing interests; and statements of data and code availability are available at 10.1038/s41589-022-01172-7.

## Supplementary information

Supplementary Information Supplementary Figs 1–17, Supplementary Tables 1–6, Supplementary Note for chemical synthesis, NMR spectra for the compounds.

Reporting Summary

Supplementary Data Information of protein sequences, antibodies, esiRNA used in this study and statistical information.

## Data Availability

The crystal structures for *mt*PanK and *ec*PanK were previously reported with Protein Data Bank ID of 4BFU and 1ESM, respectively. The data supporting the findings of this study are available within the paper and its Supplementary Information. Additional information and files are available from the corresponding author upon reasonable request. Source data are provided with this paper.

## Source data

Source Data Fig. 3 Source Spectral Data.
Source Data Fig. 4 Statistical source data.
Source Data Fig. 5 Statistical source data.
Source Data Extended Data Fig. 1 Source spectral data.
Source Data Extended Data Fig. 2 Source spectral data.
Source Data Extended Data Fig. 3 Source spectral data.
Source Data Extended Data Fig. 4 Source spectral data.
Source Data Extended Data Fig. 5 Source spectral data.
Source Data Extended Data Fig. 6 Source imaging data.
Source Data Extended Data Fig. 7 Source imaging data and calculations.
Source Data Extended Data Fig. 8 Source imaging data.
Source Data Extended Data Fig. 9 Statistical source data.
Source Data Extended Data Fig. 10 Source spectral data.
