# Supplementary material for: Probing coenzyme A homeostasis with semisynthetic biosensors
Source: Nat Chem Biol. 2022 Oct 31;19(3):346–55. doi: 10.1038/s41589-022-01172-7 (PMC9974488; doi:10.1038/s41589-022-01172-7)
Supplement: Supplementary file 1 — Supplementary Figs 1–17, Supplementary Tables 1–6, Supplementary Note for chemical synthesis, NMR spectra for the compounds. [file 41589_2022_1172_MOESM1_ESM.pdf]

nature chemical biology

Article https://doi.org/10.1038/s41589-022-01172-7

Probing coenzyme A homeostasis with semisynthetic biosensors

In the format provided by the authors and unedited

Probing coenzyme A homeostasis with semisynthetic biosensors

Lin Xue,[1,2,*] Paul Schnacke,[1] Michelle S. Frei,[1] Birgit Koch,[1] Julien Hiblot,[1] Richard Wombacher,[1] Sebastian Fabritz[1] and Kai Johnsson[1,3,*]

[1] Department of Chemical Biology, Max Planck Institute for Medical Research, Jahnstrasse 29, 69120 Heidelberg, Germany
[2] MOE Key Laboratory for Cellular Dynamics, Hefei National Center for Physical Sciences at Microscale, & University of Science and Technology of China, Hefei, China
[3]Institute of Chemical Sciences and Engineering, École Polytechnique Fédérale de Lausanne (EPFL), 1015 Lausanne, Switzerland

Email: johnsson@mr.mpg.de; linxue@ustc.edu.cn

Table of contents

1. Supplementary Figs. 1-3 Page 2

2. Supplementary Figs. 4-6 Page 3

3. Supplementary Figs. 7-8 Page 4

4. Supplementary Figs. 9-11 Page 5

5. Supplementary Figs. 12-13 Page 6

6. Supplementary Fig. 14 Page 7

7. Supplementary Fig. 15 Page 8

8. Supplementary Fig. 16 Page 9

9. Supplementary Fig. 17 Page 10

10. Supplementary Tables 1-4 Page 11

11. Supplementary Tables 5-6 Page 12

12. Supplementary Note Page 13

Supplementary Figures

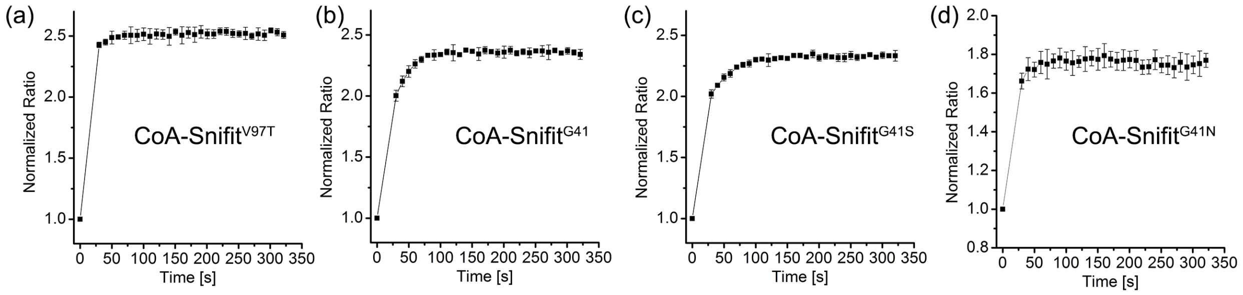

Supplementary Fig. 1 | Response kinetics of CoA-Snifits. Kinetics performed with 100 nM CoA-Snifits upon addition of (a) 100 μM CoA for CoA-Snifit^{V97T}, (b) 250 μM CoA for CoA-Snifit^{G41}, (c) 1 mM CoA for CoA-Snifit^{G41S}, and (d) 5 mM CoA for CoA-Snifit^{G41N}. The data are presented as mean ± SD, n = 3 independent replicates.

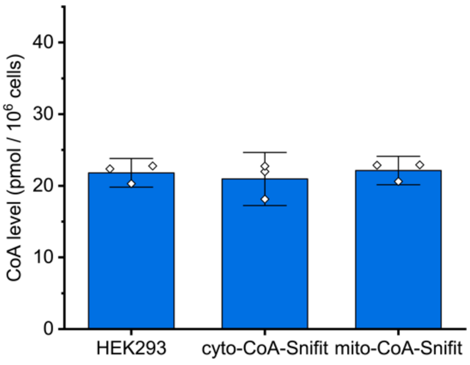

Supplementary Fig. 2 | Quantification of total CoA in cell lysates by using LC-MS/MS. The total CoA levels were measured by analyzing the cell lysate from the HEK293 cells without expression of sensor protein, the HEK293 cells stably expressing cytosolic apo-CoA-Snifit^{V97T}, or mitochondrial apo-CoA-Snifit^{G41S}. The data are presented as mean ± SD, n = 3 independent samples.

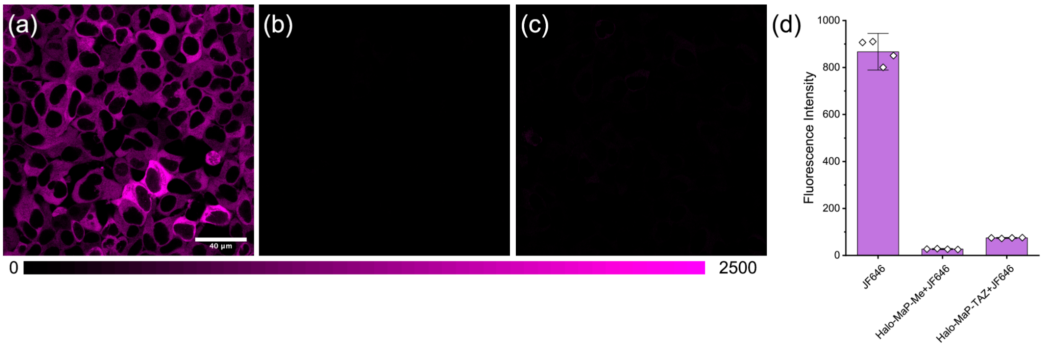

Supplementary Fig. 3 | Evaluating labeling efficiency of fluorescent probes in living cells. Fluorescence images of HEK293 cells stably expressing cytosolic apo-CoA-Snifit^{V97T} induced with 400 ng/mL doxycycline for 12 h. (a) Cells were labeled with 1 μM JF646 for 30 min. (b) Cells were labeled with 1 μM Halo-MaP-Me for 12 h, and further labeled with 1 μM JF646 for 30 min. (c) Cells were labeled with 1 μM Halo-MaP-TAZ for 12 h, and further labeled with 1 μM JF646 for 30 min. Scale bar: 40 μm. (d) Average fluorescence intensity of JF646 for (a), (b), and (c). λ_{ex} = 633 nm, λ_{em} = 650 – 720 nm. The data are presented as mean ± SD, n = 4 field of views (FOVs) over 4 independent samples with > 50 cells per FOV.

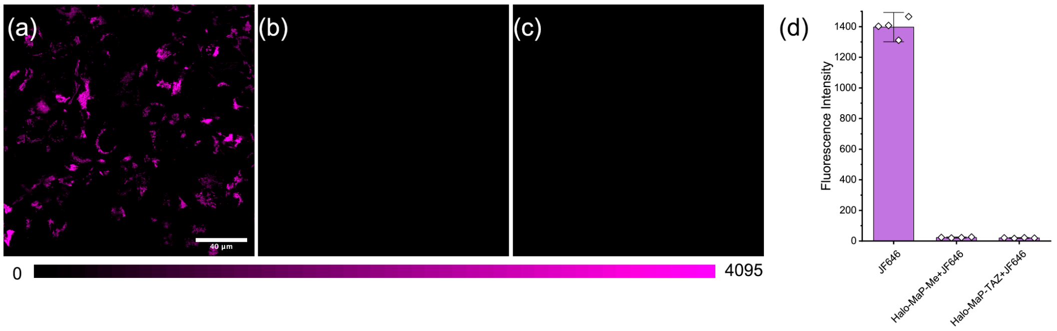

Supplementary Fig. 4 | Labeling efficiency of the fluorescent probes in living cells. Fluorescence images of the HEK293 cells stably expressing mitochondrial apo-CoA-Snifit^{G41S} induced with 400 ng/mL doxycycline for 12 h. (a) Cells were labeled with 1 µM JF646 for 30 min. (b) Cells were labeled with 1 µM Halo-MaP-Me for 12 h, and further labeled with 1 µM JF646 for 30 min. (c) Cells were labeled with 1 µM Halo-MaP-TAZ for 12 h, and further labeled with 1 µM JF646 for 30 min. Scale bar: 40 µm. (d) Average fluorescence intensity of JF646 for (a), (b), and (c). λ_{ex} = 633 nm, λ_{em} = 650 – 720 nm. The data are presented as mean ± SD, n = 4 field of views (FOVs) over 4 independent samples with > 50 cells per FOV.

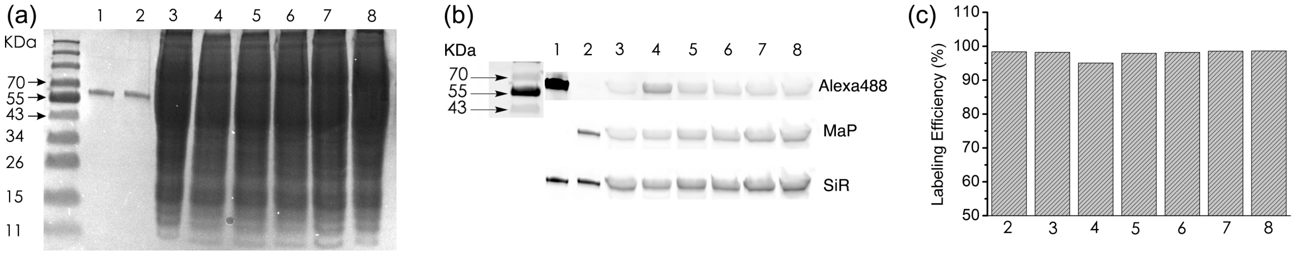

Supplementary Fig. 5 | Evaluating the labeling efficiency of the probe Halo-MaP-TAZ by in-gel (SDS-PAGE) fluorescence scanning. (a) Representative coomassie staining after protein SDS-PAGE (n = 2 independent experiments). (b) The in-gel fluorescence of the same region of interest (ROI) in the Cy2 (Alexa-488), Cy3 (MaP), and Cy5 (SiR) channels. (c) Calculated labeling efficiency of the probe. Lane 1: protein expressed from *e.coli* is labeled with BG-SiR and Halo-Alexa488, lane 2: protein expressed from *e.coli* is labeled with BG-SiR and Halo-MaP-TAZ for 2 h and then labeled with Halo-Alexa488 for 30 min, lane 3: HEK293 cells stably expressing Halo-SNAP protein were induced with 400 ng/mL doxycycline for 12 h and lysed. The lysate was labeled with BG-SiR and Halo-MaP-TAZ for 2 h. lane 4 to 8: the HEK293 cells stably expressing Halo_SNAP protein were induced with 400 ng/mL doxycycline for 12 h and labeled with BG-SiR for 3 h and Halo-MaP-TAZ for 2, 4, 6, 9, 12 h, respectively. The cells are then lysed and labeled with Halo-Alexa488 for 30 min. Please see the **Supplementary Fig. 17** for the uncropped figures.

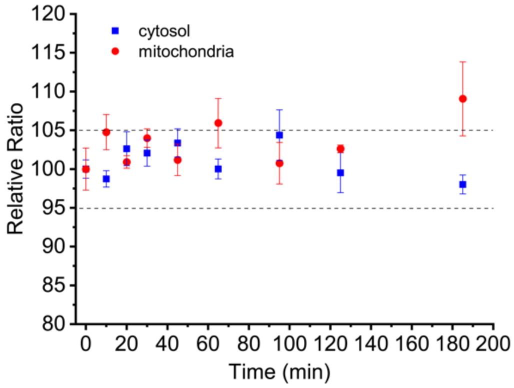

Supplementary Fig. 6 | Stability test of FRET ratio measured in HEK293 cells in continuous measurements. Relative ratio changes were measured during 3 h for cytosolic holo-CoA-Snifit^{V97T} and mitochondrial holo-CoA-Snifit^{G41S}. All the ratio values are normalized to the first data point (reported as 100%) and are presented as mean ± SD, n = 3 field of views (FOVs) over 3 independent samples with > 50 cells per FOV. The dashed lines show the ± 5% variation of the first data point.

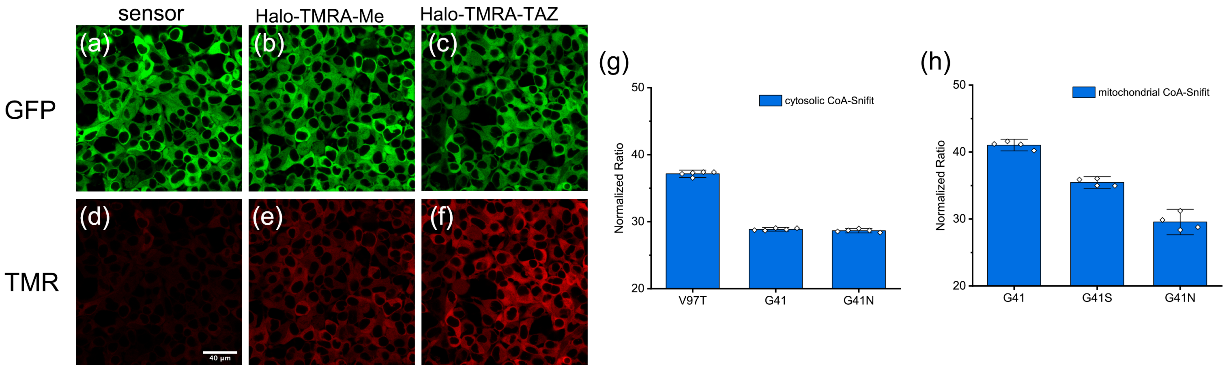

Supplementary Fig. 7 | Evaluation of CoA-Snifits in living HEK293 cells. (a)-(f) Representative fluorescence images of the cells expressing cytosolic sensor protein of CoA-Snifit^{V97T} without labeling (a and d), with 1 μM Halo-MaP-Me labeling for 12 h (b and e), with 1 μM Halo-MaP-TAZ labeling for 12 h (c and f). The fluorescence emission of (a)-(c) and (d)-(f) were collected in the GFP channel and MaP channel, respectively. (g and h) Normalized ratio values were calculated from the fluorescence images of cytosolic CoA-Snifits (g) and mitochondrial CoA-Snifits (h) respectively. The data are presented as mean ± SD, n = 3 field of views (FOVs) over 3 independent samples with > 50 cells per FOV.

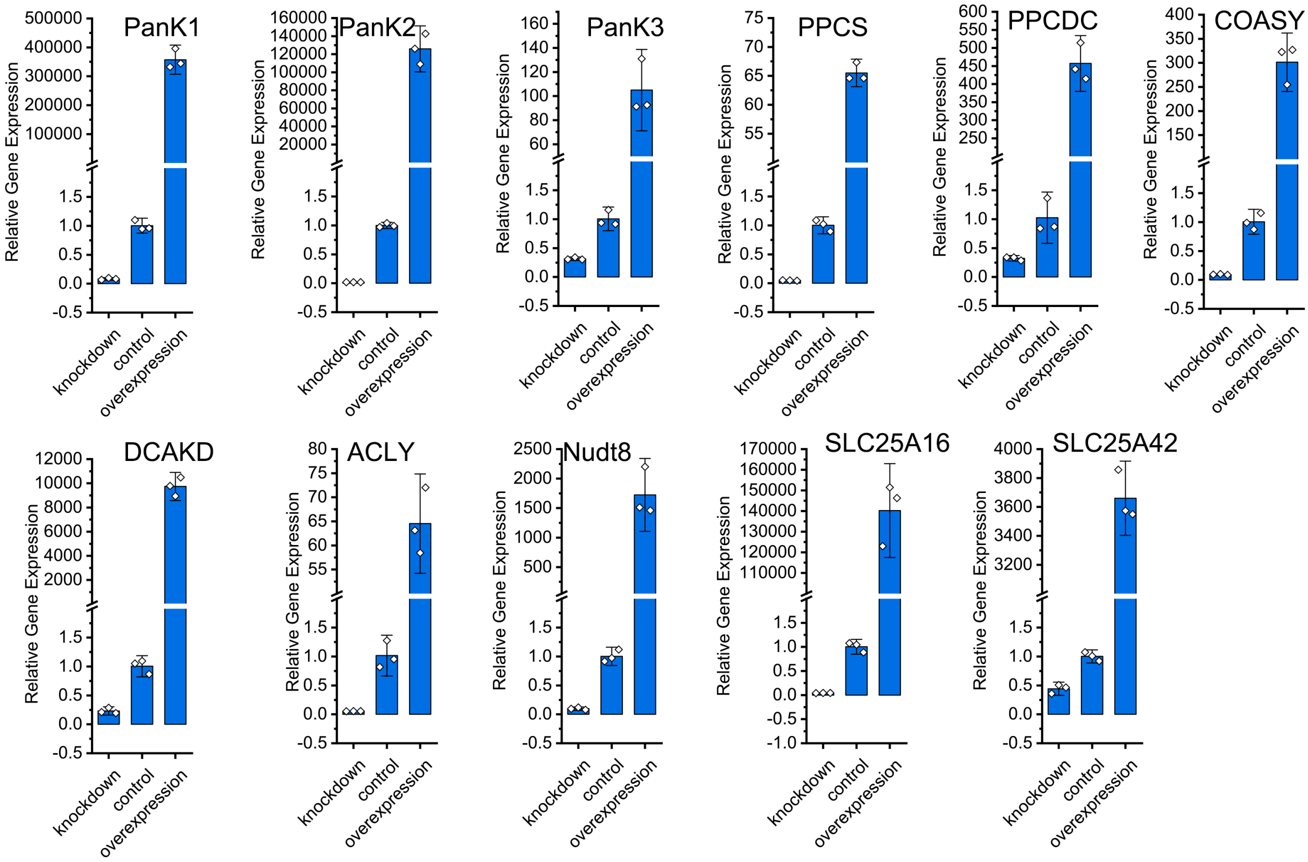

Supplementary Fig. 8 | The relative mRNA level was evaluated by quantitative RT-PCR (qPCR) experiments. Lysate of HEK293 cells was collected after gene knocking down for 72 h or gene overexpression for 24 h. The RNA was isolated and prepared for qPCR. Data were normalized to GAPDH (glyceraldehyde-3-phosphate dehydrogenase) expression. The untreated HEK293 cells were used as a negative control. The data are presented as mean ± SD, n = 3 independent replicates.

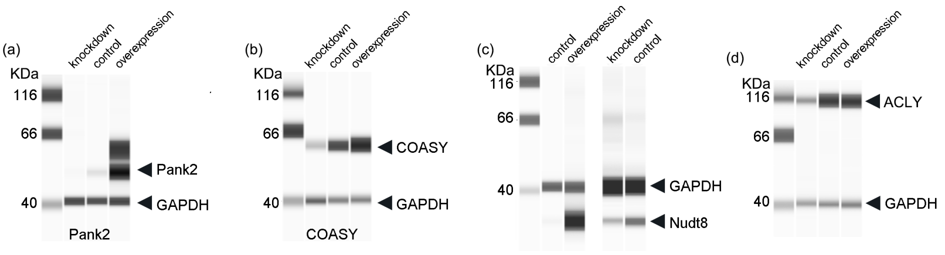

Supplementary Fig. 9 | Western Blot analysis of gene-knockdown or overexpression of proteins in HEK293 cells. Representative (n =2 independent experiments) western blots of HEK293 cells which were transiently transfected with plasmids encoding the indicated genes (overexpression) or esiRNA for the knockdown of the proteins in cells. GAPDH was used as the loading control.

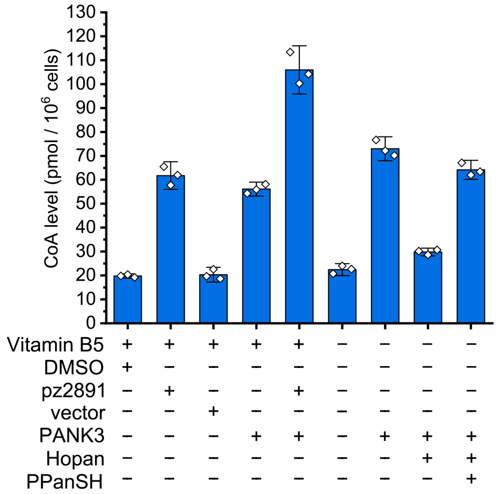

Supplementary Fig. 10 | The total CoA level was measured by LC-MS/MS. HEK293 cells were transfected with either an empty vector or vector encoding PANK3, and cells were treated with 1 μM PZ-2891, 400 μM HoPan, or 100 μM PPanSH for 24 h in the full growth medium or vitamin B_5-free medium (for HoPan treatment). The data are presented as mean ± SEM, n = 3 independent samples.

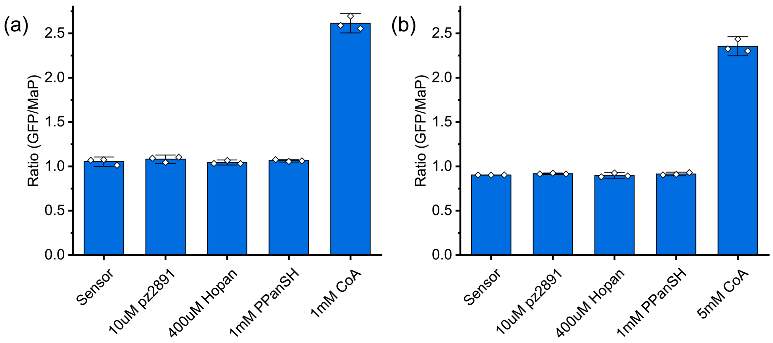

Supplementary Fig. 11 | In vitro specificity test of CoA-Snifits to PZ2891, HoPan, and PPanSH. The ratio (GFP/MAP) responses of 100 nM (a) CoA-Snifit^{V97T} and (b) CoA-Snifit^{G41S} in the presence of 10 μM PZ-2891, 400 μM HoPan, 1mM PPanSH, and 1mM CoA/5mM CoA in PBS. These titration results suggested that PZ-2891, HoPan and PPanSH did not directly interact with CoA-Snifits. The data are presented as mean ± SD, n = 3 independent replicates.

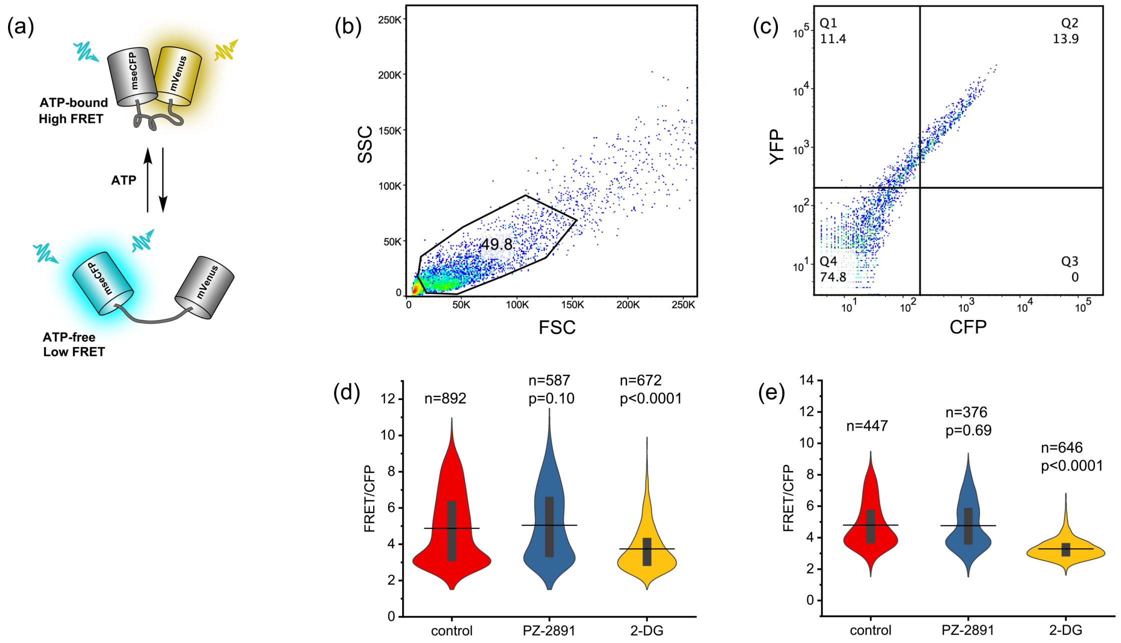

Supplementary Fig. 12 | The variations of cellular ATP concentration were evaluated by ATeam sensors. (a) the design of ATeam sensors. Gating strategy involved the removal of dead cells and debris (FSC vs SSC) (b) and selection of the cell population that expressing the ATeam sensors (CFP vs YFP, Q2) (c). (d)-(e) the FRET ratio response (FRET/CFP) of the cytosolic sensor (d) and mitochondrial sensor (e) in the presence of 1 μM PZ-2891 and 10 mM 2-deoxy-D-glucose (2-DG). The data are shown as violin plots of the FRET ratio (FRET/CFP) for the cells (n and p values are indicated in (d) and (e)). The central lines in the violins indicated the mean value of FRET ratio. Black boxes show the 25 and 75% percentile. The results demonstrated that the PZ-2891 did not induce obvious ATP changes (as judged by changes in FRET ratio) and 2-DG, which was selected as the positive control, resulted in a 24% and 20% decrease in the FRET ratio in cytosol and mitochondria, respectively, indicating the expected significant ATP decrease. *P ≤ 0.05, **P ≤ 0.01, ***P ≤ 0.001, NS p > 0.05, two-tailed unpaired t-test.

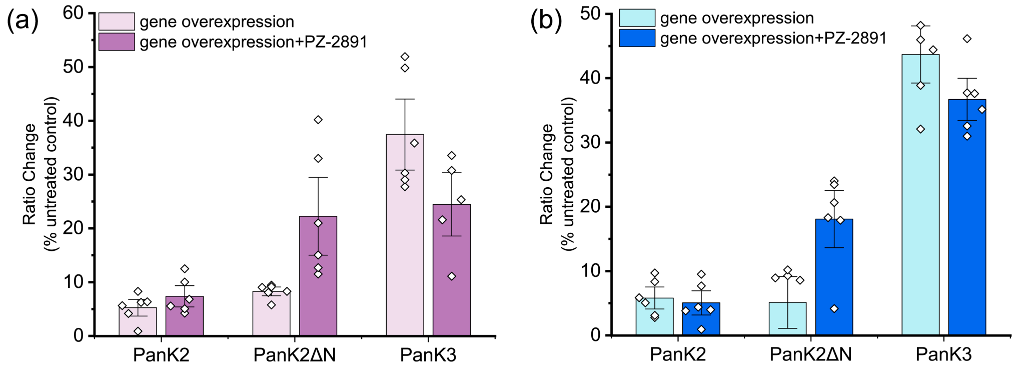

Supplementary Fig. 13 | Fluorescence ratio changes (mean ± SEM) of CoA-Snifit upon gene overexpression. (a) cytosolic CoA-Snifit^{V97T} and (b) mitochondrial CoA-Snifit^{G41S} were overexpressed with Pank2, PanK2ΔN, and PanK3 in the absence and presence of 1 μM PZ-2891. For gene overexpression experiments, the empty-vector-transfected cells were used as control. For gene overexpression in the presence of PZ-2891, the cells transfected with empty vector and further treated with 1 μM PZ-2891, were used as control. The data are presented as mean ± SEM, n = 5 or 6 field of views (FOVs) over 4 independent samples with > 50 cells per FOV. The precise n and p values are listed in Supplementary Data.

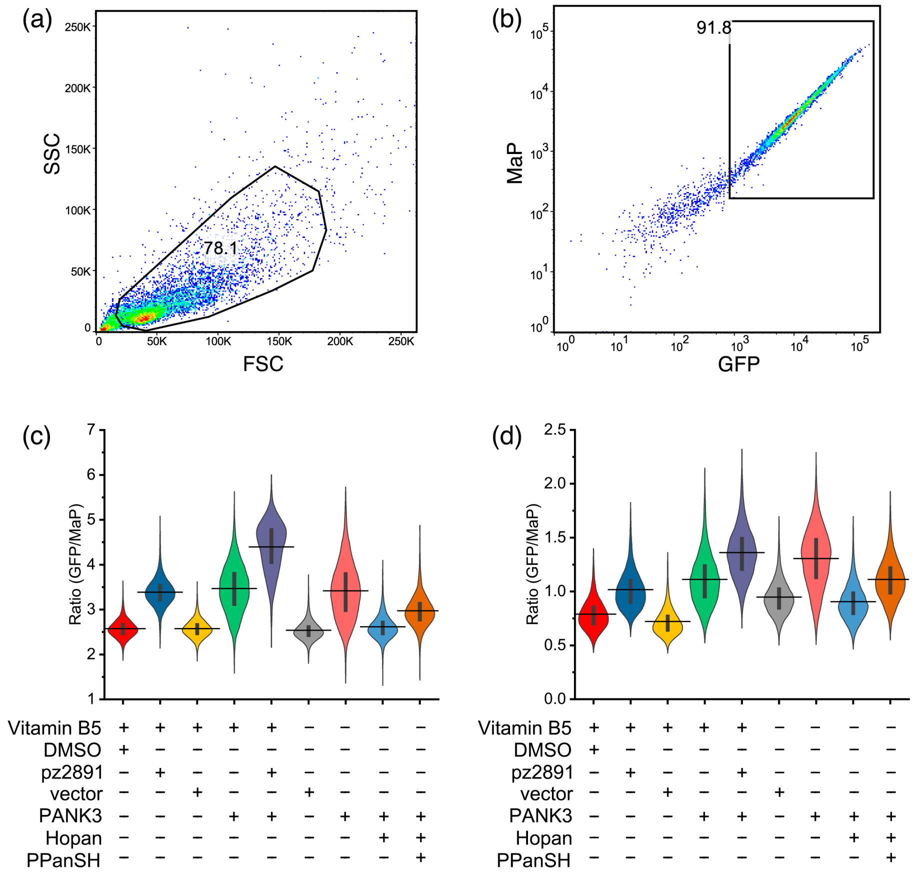

Supplementary Fig. 14 | Regulation of CoA in HEK293 cells. FRET ratios as measured by flow cytometry of CoA-Snifits. Gating strategy involved the removal of dead cells and debris (FSC vs SSC) (a) and selection of the labeled cell population (GFP vs MaP) (b). FRET ratio values (GFP/MaP) were calculated from cytosolic CoA-Snifit^{V97T} (c) and mitochondrial CoA-Snifit^{G41S} (d) under the indicated conditions (0.05% DMSO, 1 μM PZ-2891, 400 μM HoPan, and 100 μM PPanSH). The data are shown as violin plots of the FRET ratio (GFP/MaP) for the cells (n = 18000). The central lines in the violins indicate the mean value of FRET ratio. Black boxes show the 25 and 75% percentile.

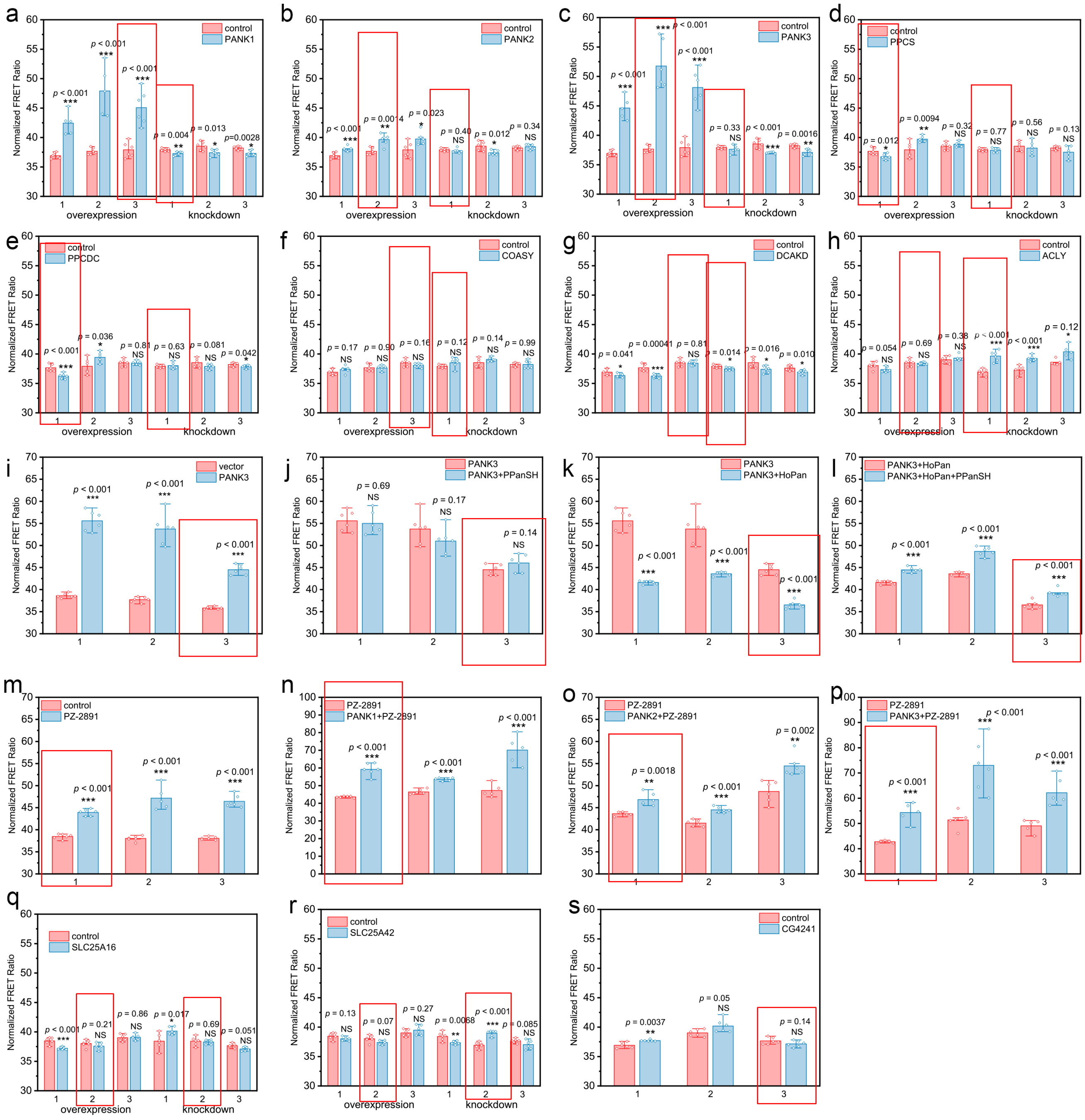

Supplementary Fig. 15 | Biological replicates of all imaging experiments performed with cytosolic sensor CoA-Snifit^{V97T}. The ratio values are shown as mean ± SEM, n = 5 or 6 field of views (FOVs) over 4 independent samples with > 50 cells per FOV. The precise n and p values are listed in Supplementary Data. * P < 0.05, ** P < 0.01, *** P < 0.001, NS p > 0.05, two-tailed unpaired t-test. (a-h) Biological replicates for data shown in Figure 4a. Overexpression (empty PCDNA5 vector as control) and knockdown (siFLUC as the control) of proteins. (i-l) Biological replicates for data shown in Figure 4d. The experiments were performed in VB5-free medium. (m-p) Biological replicates for data shown in Figure 4f. (q-s) Biological replicates for data shown in Figure 5a. Overexpression (empty PCDNA5 vector as control) and knockdown (siFLUC as the control) of proteins. The arbitrarily chosen biological replicates for each experiment shown in Figure 4 or Figure 5a (main text) are highlighted with red rectangles.

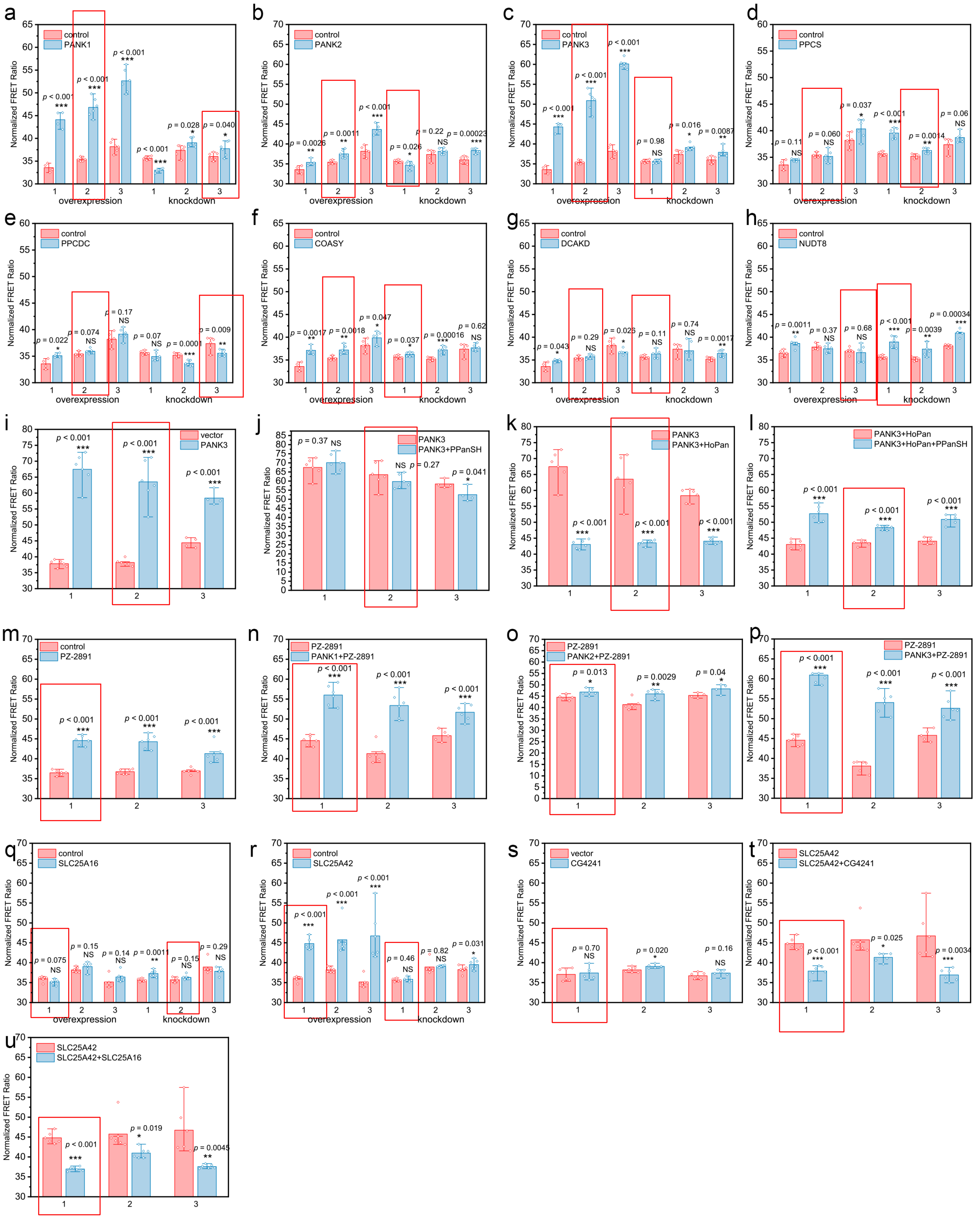

Supplementary Fig. 16 | Biological replicates of all imaging experiments performed with mitochondrial sensor CoA-Snifit^{G41S}. The ratio values are shown as mean ± SEM, n = 4, 5 or 6 field of views (FOVs) over 4 independent samples with > 50 cells per FOV. The precise n and p values are listed in Supplementary Data. * P < 0.05, ** P < 0.01, *** P < 0.001, NS p > 0.05, two-tailed unpaired t-test. (a-h) Biological replicates for data shown in Figure 4b. Overexpression (empty PCDNA5 vector as control) and knockdown (siFLUC as the control) of proteins. (i-l) Biological replicates for data shown in Figure 4e. The experiments were performed in VB5-free medium. (m-p) Biological replicates for data shown in Figure 4g. (q-u) Biological replicates for data shown in Figure 5b. Overexpression (empty PCDNA5 vector as control) and knockdown (siFLUC as the control) of proteins. The arbitrarily chosen biological replicates for each experiment shown in Figure 4 or 5b (main text) are highlighted with red rectangles.

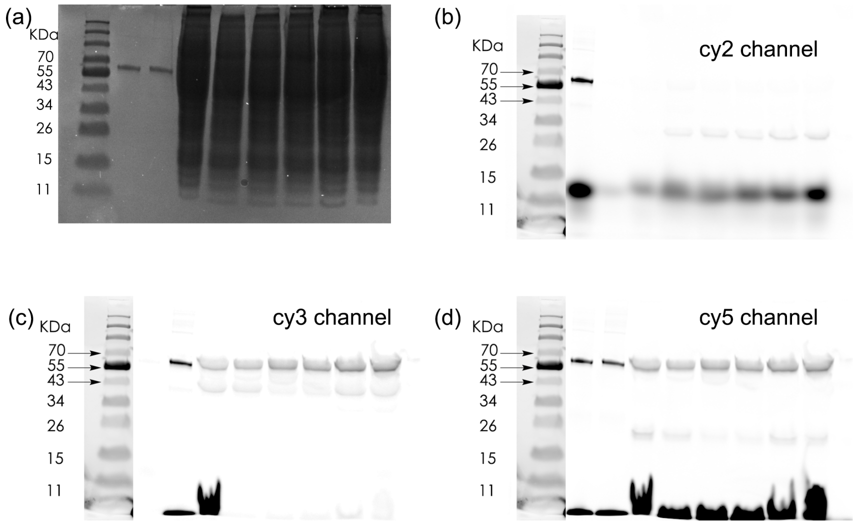

Supplementary Fig. 17 | Uncropped scans of gels for Supplementary Fig 5. The same gels were scanned in bright field (a), Cy2 channel (b), Cy3 channel (c), and Cy5 channel (d). Note that the marker for (b) and (c) were copied from Cy5 channel, because the marker did not show fluorescence in the Cy2 and Cy3 channel, but showed weak fluorescence (the contrast was adjusted for clarity) in Cy5 channel.

Supplementary Tables

Supplementary Table 1. Affinity of ecPanK variants to CoA and AcCoA as measured by fluorescence polarization.[a]

Proteins	K_d for TMR-TAZ (μM)	c_{50} for CoA (μM)	c_{50} for AcCoA (μM)	Ratio (AcCoA/CoA) of c_{50}
ecPanK^{WT}	1.2 ± 0.2	214.1 ± 19.2	1420.0 ± 315.4	6.6
ecPanK^{F252Y}	0.4 ± 0.1	2240 ± 449	12960 ± 976	5.8
ecPanK^{L277F}	0.5 ± 0.0	119.2 ± 6.1	1420 ± 167	11.9
ecPanK^{L277Y}	1.3 ± 0.2	43.2 ± 4.5	598.0 ± 83.1	13.8
ecPanK^{L277W}	1.1 ± 0.2	31.7 ± 6.4	471.1 ± 68.9	14.9
ecPanK^{F252Y,L277W}	0.5 ± 0.0	137.5 ± 6.8	2870 ± 129	20.9
ecPanK^{D127,F252Y,L277W}	21.6 ± 3.7	1.0 ± 0.5	19.6 ± 1.5	19.6

[a] The measurement was performed in PBS buffer (50 mM HEPES, 0.5 mg/mL BSA, pH = 7.4) and the signal is collected on microplate reader. The data were presented as mean ± SD, n = 3 independent replicates.

Supplementary Table 2. Affinity, dynamic range and specificity of the CoA-Snifits.[a]

Sensor	ΔR_{max}	c_{50} (μM)			Ratio of c_{50}	Ratio of c_{50}
		CoA	AcCoA	dPCoA	(AcCoA/CoA)	(dPCoA/CoA)
CoA-Snifit^{V97T}	2.5	2.4 ± 0.2	94.3 ± 10.6	1310 ± 78	39.3	545.8
CoA-Snifit^{G41}	2.8	13.9 ± 1.9	808.8 ± 48.1	2800 ± 223	58.2	201.4
CoA-Snifit^{G41S}	2.6	58.9 ± 5.6	3660 ± 218	2340 ± 407	62.1	39.7
CoA-Snifit^{G41N}	2.4	956.5 ± 132.3	11230 ± 157	1650 ± 122	11.7	1.7

[a] The measurement was performed in PBS buffer (50 mM HEPES, 0.5 mg/mL BSA, pH = 7.4) and the signal is collected on microplate reader. The data were presented as mean ± SD, n = 3 independent replicates.

Supplementary Table 3. Effect of ATP and ADP on CoA-Snifits.[a]

	c_{50} of CoA-Snifit^{V97T}(μM)		c_{50} of CoA-Snifit^{G41}(μM)		c_{50} of CoA-Snifit^{G41S}(μM)	
Total [AXP]	[ATP/[ADP]=9:1	[ATP]/[ADP]=99:1	[ATP/[ADP]=9:1	[ATP]/[ADP]=99:1	[ATP/[ADP]=9:1	[ATP]/[ADP]=99:1
0.5 mM	4.8 ± 0.4	5.6 ± 0.2	15.7± 0.7	15.4 ± 1.5	86.0 ± 5.4	75.8 ± 6.7
1.0 mM	5.5 ± 0.3	7.2 ± 0.5	15.9 ± 0.9	19.3 ± 0.9	77.2 ± 7.3	94.2 ± 10.0
2.0 mM	9.3 ± 1.6	9.6 ± 0.5	21.2 ± 1.0	23.5 ± 2.2	99.0 ± 9.6	106.7 ± 12.1

[a] The measurement was performed in PBS buffer (50 mM HEPES, 0.5 mg/mL BSA, pH = 7.4) in the presence of different concentrations of total AXP. The data were presented as mean ± SD. n = 3 independent replicates.

Supplementary Table 4. Comparison of affinity and dynamic range of recombinant CoA-Snifits in buffer and of CoA-Snifits in lysates of HEK293 cells [a]

	c_{50} (μM) ± SD		R_{min}		R_{max}	
Sensor	*E.coli*	HEK293	*E.coli*	HEK293	*E.coli*	HEK293
CoA-Snifit^{V97T}	3.9 ± 0.3	5.8± 0.8	1.07	1.20	2.50	2.64
CoA-Snifit^{G41}	13.9± 1.9	37.7± 2.2	0.94	1.07	2.61	2.55
CoA-Snifit^{41S}	58.9 ± 5.6	141.6 ± 11.3	0.90	1.00	2.35	2.29

[a] The data were presented as mean ± SD. n = 3 independent replicates.

Supplementary Table 5. Quantification of CoA using fluorescence lifetimes of CoA-Snifits.

Cell lines	Localization[a]	<τ> (ns) ± SD	E (%) ± SD	Concentration (μM) ± SD [b]	Whole cell (fpmol/10^6 cells)[c]	Cells counts
Donor-only[d]	cytosol	2.11 ± 0.02	-	-		> 50 (from 3 imaging dishes)
	mitochondria	1.96 ± 0.02	-	-		> 50 (from 3 imaging dishes)
HEK293	cytosol	1.36 ± 0.02	35.5 ± 0.6	81 ± 11	25.4 ± 1.5	> 100 (10 imaging dishes from 3 biological replicates)
	mitochondria	1.25 ± 0.02	36.1 ± 0.8	258 ± 28		> 100 (10 imaging dishes from 3 biological replicates)
HeLa	cytosol	1.36 ± 0.02	35.7 ± 0.9	79 ± 16	30.4 ± 4.5	> 50 (8 imaging dishes from 2 biological replicates)
	mitochondria	1.28 ± 0.03	35.0 ± 1.3	308 ± 59		> 50 (7 imaging dishes from 3 biological replicates)
U-2 OS	cytosol	1.34 ± 0.02	36.5 ± 1.0	67 ± 13	39.0 ± 4.7	> 50 (12 imaging dishes from 4 biological replicates)
	mitochondria	1.40 ± 0.02	28.7 ± 0.9	816 ± 148		> 50 (10 imaging dishes from 4 biological replicates)
HepG2	cytosol	1.36 ± 0.03	35.4 ± 1.5	85 ± 24	58.1 ± 5.5	24 (8 imaging dishes from 2 biological replicates)
	mitochondria	1.40 ± 0.02	28.8 ± 0.8	794 ± 106		31 (11 imaging dishes from 3 biological replicates)

[a] The free cytosolic and mitochondrional CoA concentrations were measured by using cytosolic Co-Snifit^{G41} and mitochondrial Co-Snifit^{G41S} with FLIM in living cells, respectively. [b] The free CoA concentration was measured by measuring the basal FLIM signal of the sensors. [c] Total cellular CoA was measured by LC-MS/MS. [d] The unlabeled sensors were used as the donor-only control.

Supplementary Table 6. The multiple reaction monitoring (MRM) transitions for MS/MS analysis.

	m/z	declustering potential (V)	collision energy (V)	cell exit potential (V)	dwell time (ms)
CoA1	768.1→261.1	60	39	12.3	100
CoA2	768.1→428.0	60	32	16.8	50
dPCoA	688.2→261.1	60	39	12.3	20
CoA-MA1	985.1→478.1	30	42	18	75
CoA-MA2	985.1→428.1	30	39.5	17	50

Supplementary Note

General considerations of chemistry.

All reactions were carried out in oven-dried glassware under nitrogen atmosphere, unless stated otherwise. Commercial chemicals were purchased from Sigma-Aldrich, Fisher Scientific, Merck, Alfa Aesar, Axon Lab or Reactolab and used without further purification.

Anhydrous solvents from Acros were used without further treatment. Flash column chromatography was performed with silica gel (230-400 mesh). Reverse-phase semi-preparative high-pressure liquid chromatography was performed on a Thermo Fisher ultimate 3000 system equipped with an Ultimate 3000 pump and an UVD 170U UV-Vis detector for product visualization with a SUPELCO ascentis c18 HPLC column (5 µm, 25 cm × 21.2 mm) or a SUPELCO ascentis c18 HPLC column (5 µm, 25 cm × 10 mm). Buffer A: 0.1% v/v TFA in H_2O, Buffer B: acetonitrile.

Nuclear magnetic resonance (NMR) spectra were recorded at room temperature on a Bruker Avance-III 400 with chemical shifts (δ) reported in ppm relative to the solvent residual signals. $CDCl_3$: H 7.26 ppm, C 77.16 ppm, DMSO-d_6: H 2.5 ppm, C 39.52 ppm, CD_3CN: H 1.94 ppm, C 1.32 ppm, MeOD: H 3.31 ppm, C 49.00 ppm. Coupling constants are reported in Hz. High resolution mass spectra (HRMS) were measured on a maXis II ETD HRMS system.

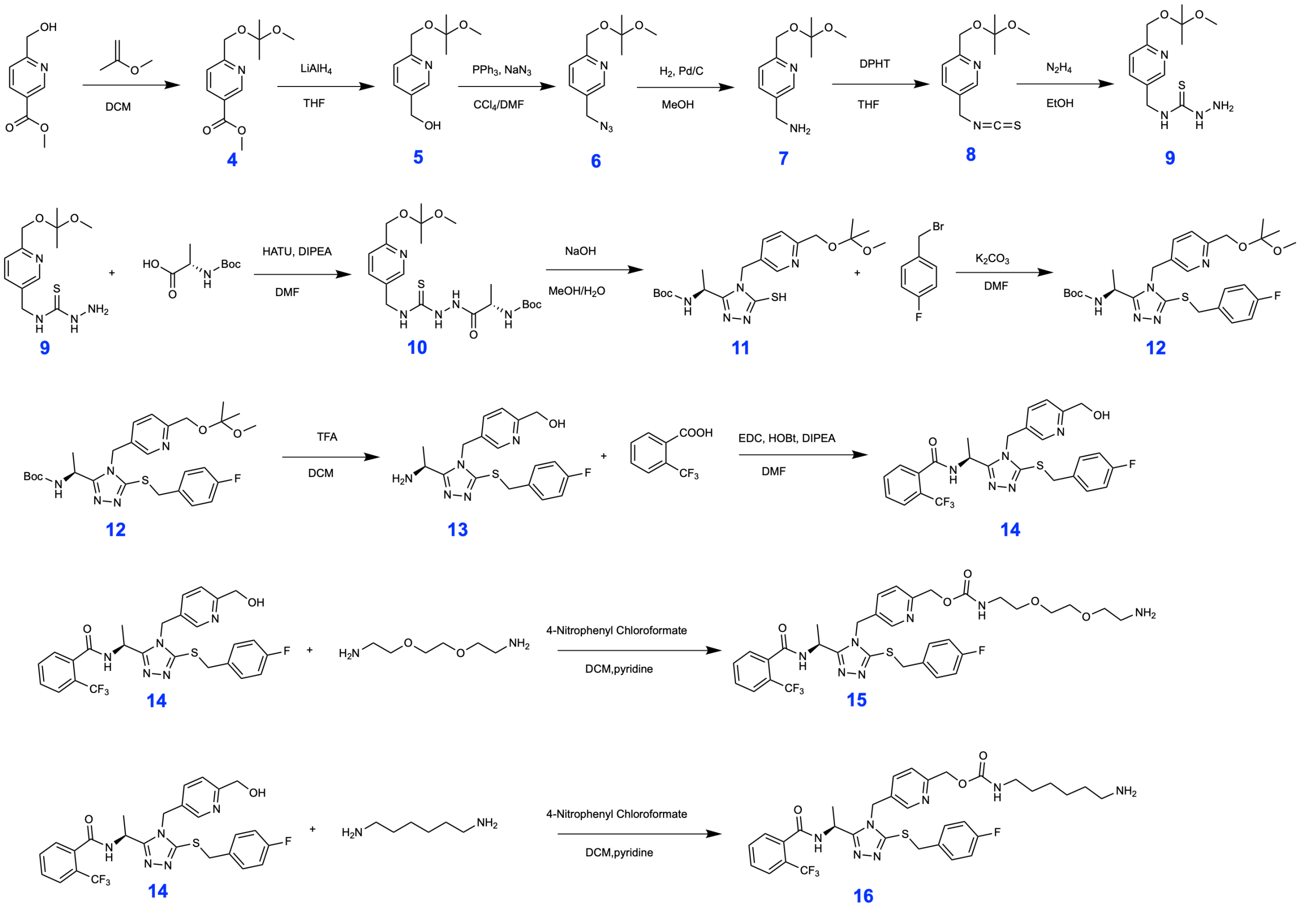

Supplementary Scheme 1. Synthetic route of intermediate compounds.

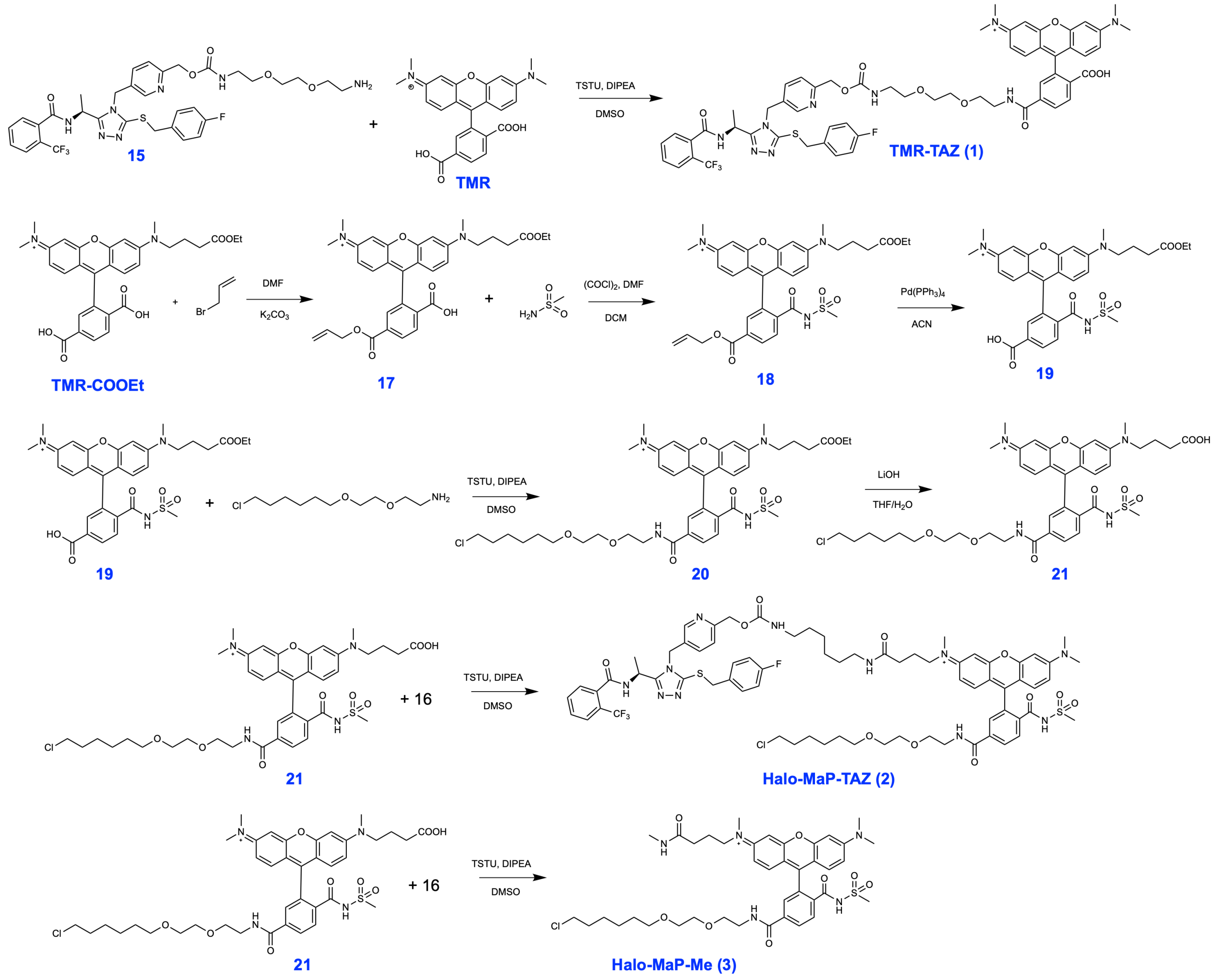

Supplementary Scheme 2. Synthetic route of probes.

Compound 4. To the solution of methyl 6-(hydroxymethyl)nicotinate (0.5 g, 3 mmol) in 20 mL CH_2Cl_2, pyridinium *p*-toluenesulfonate (75 mg, 0.3 mmol) and 2-methoxypropene were (0.65 g, 9 mmol) were added. After stirring at room temperature for 3 h, the solvent was removed by vacuum. The residue was purified by flash chromatography (hexane/0-50% ethyl acetate) to give compound **4** as a colorless oil (0.65 g, 90% yield). **^{1}H NMR** (400 MHz, $CDCl_3$) δ 9.10 (dd, J = 2.16, 0.85 Hz, 1H), 8.25 (dd, J = 8.14, 2.15 Hz, 1H), 7.55 (dq, J = 8.18, 0.84 Hz, 1H), 4.64 (d, J = 0.72 Hz, 2H), 3.91 (s, 3H), 3.20 (s, 3H), 1.42 (s, 6H). **^{13}C NMR** (100 MHz, $CDCl_3$) δ 165.88, 164.04, 150.32, 137.78, 124.52, 120.36, 100.75, 63.85, 52.37, 48.83, 24.55. **HRMS** (ESI, pos. mode) m/z calc. for $C_8H_{10}NO_3^+$ 168.0661, found 168.0655 $[M\text{-}MOP+H]^+$. *Note: The MOP protection group was removed under the weak acidic condition of MS measurements.*

Compound 5. To the solution of compound **4** (0.48 g, 2 mmol) in 20 mL dry THF, $LiAlH_4$ (1.1 mL, 2 M solution in THF) was gradually added at 0 ºC. After stirring at room temperature for 3 h, TLC monitoring was performed. When the reaction was completed, brine (50 mL) was added carefully and the resulting mixture was extracted with ethyl acetate (30 mL). The organic phase was washed with brine (3 × 50 mL), dried over Na_2SO_4, and concentrated under reduced pressure. The residue was purified by flash chromatography (ethyl acetate/0-10% MeOH) to give compound **5** as a colorless oil (0.39 g, 92% yield). **^{1}H NMR** (400 MHz, $CDCl_3$) δ 8.47 (s, 1H), 7.71 (dd, J = 8.1, 2.2 Hz, 1H), 7.46 (d, J = 8.0 Hz, 1H), 4.70 (s, 2H), 4.60 (s, 2H), 3.23 (s, 3H), 1.43 (s, 6H). **^{13}C NMR** (100 MHz, $CDCl_3$) δ 158.65, 147.93, 135.78, 134.84, 121.16, 100.73, 63.95, 62.67, 48.85, 24.64. **HRMS** (ESI, pos. mode) m/z calc. for $C_{11}H_{17}NNaO_3^+$ 234.1106, found 234.1101 $[M+Na]^+$.

Compound 6. A mixture of compound **5** (0.42 g, 2 mmol), sodium azide (0.26 g, 4 mmol) and PPh_3 (0.58 g, 2.2 mmol) in 30 mL mixture of CCl_4-DMF (1:4, v/v) was warmed at 60 ºC with stirring. After full disappearance of starting material (monitored by TLC), the mixture was cooled to room temperature and quenched by addition of 5 mL water. After stirring for 10 min, the mixture was extracted with ethyl acetate (3 × 30 mL). The organic phase was washed with brine (3 × 50 mL), dried over Na_2SO_4, and concentrated under reduced pressure. The residue was purified by flash chromatography (hexane/0-70% ethyl acetate) to give compound **6** as a yellow oil (0.38 g, 80% yield). **^{1}H NMR** (400 MHz, $CDCl_3$) δ 8.50 (dd, J = 2.3, 0.8 Hz, 1H), 7.67 (dd, J = 8.1, 2.3 Hz, 1H), 7.51 (dd, J = 8.0, 0.8 Hz, 1H), 4.63 (s, 2H), 4.37 (s, 2H), 3.24 (s, 3H), 1.45 (s, 6H). **^{13}C NMR** (100 MHz, $CDCl_3$) δ 159.69, 148.79, 136.60, 129.56, 121.25, 100.75, 63.93, 52.13, 48.88, 24.65. **HRMS** (ESI, pos. mode) m/z calc. for $C_7H_9N_4O^+$ 165.0776, found 165.0771 $[M\text{-}MOP+H]^+$.

Compound 8. A solution of compound **6** (0.5 g, 2.1 mmol) and 10% Pd/C (50 mg) in 20 mL MeOH was stirred at room temperature with H_2 gas for 12 h. After the solvent was evaporated, the crude compound **7** (0.42 g) was obtained as colorless oil and used without further purification. To a solution of di-2-pyridyl thiocarbonate (DPT, 0.43 g, 1.9 mmol) and DIPEA (1.0 mL, 5.7 mmol) in dry THF (20 mL), a solution of compound **7** (0.42 g, crude) in dry THF (10 mL) was added dropwise. After total disappearance of starting material (monitored by TLC), the solvent was removed by vacuum. The residue was dissolved in ethyl acetate (30 mL), washed with brine (3 × 50 mL), dried over Na_2SO_4, and concentrated under reduced pressure. The residue was purified by flash chromatography (hexane/0-70% ethyl acetate) to give compound **8** as a yellow oil (0.27 g, 51% two-step yield). **^{1}H NMR** (400 MHz, $CDCl_3$) δ 8.50 (dd, J = 2.3, 0.8 Hz, 1H), 7.70 (dd, J = 8.1, 2.4 Hz, 1H), 7.53 (dd, J = 8.1, 0.8 Hz, 1H), 4.73 (s, 2H), 4.63 (s, 2H), 3.24 (s, 3H), 1.45 (s, 6H). **^{13}C NMR** (100 MHz, $CDCl_3$) δ 159.95, 147.73, 135.52, 128.63, 121.37, 100.77, 63.86, 48.90, 46.43, 24.65. **HRMS** (ESI, pos. mode) m/z calc. for $C_{12}H_{16}N_2NaO_2S^+$ 275.0830, found 275.0824 $[M+Na]^+$.

Compound 9. To a solution of compound **8** (0.30 g, 1.2 mmol) in 20 mL ethanol, a solution of $N_2H_4{\cdot}H_2O$ (90 µL, 1.8 mmol) in 5 mL ethanol were gradually added. After stirring at room temperature for 30 min, the solvent was evaporated. The residue was purified by flash chromatography (ethyl acetate /0-10% MeOH) to give compound **9** as a white solid (0.27 g, 79% yield). **^{1}H NMR** (400 MHz, $CDCl_3$) δ 8.75 – 8.45 (m, 1H), 7.76 (dd, J = 8.1, 2.3 Hz, 1H), 7.72 (s, 1H), 7.46 (d, J = 8.0 Hz, 1H), 7.20 (d, J = 5.2 Hz, 1H), 4.89 (d, J = 5.9 Hz, 2H), 4.61 (s, 2H), 3.25 (s, 3H), 1.44 (s, 6H). **^{13}C NMR** (100 MHz, $CDCl_3$) δ 182.34, 158.33, 148.44, 136.34, 132.17, 121.17, 100.57, 63.84, 48.71, 44.89, 24.50. **HRMS** (ESI, pos. mode) m/z calc. for $C_8H_{13}N_4OS^+$ 213.0810, found 213.0804 $[M\text{-}MOP+H]^+$.

Compound 10. To a solution of Boc-Ala-OH (0.19 g, 1 mmol) and DIPEA (0.53 mL, 3 mmol) in 15 mL dry DMF, a solution of HATU (0.38 g, 1 mmol) in 5 mL DMF was added. The resulting mixture was stirred at room temperature for 30 min, and then compound **9** (0.26 g, 0.9 mmol) was added. After stirring at room temperature overnight, brine (50 mL) was added carefully and the resulting mixture was extracted with ethyl acetate (3 × 30 mL). The organic phase was washed with brine (3 × 100 mL), dried over Na_2SO_4, and concentrated under reduced pressure. The residue was purified by flash chromatography (hexane/0-100% ethyl acetate) to give compound **10** as a yellow oil (0.4 g, 88% yield). **^{1}H NMR** (400 MHz, MeOD) δ 8.44 (dd, J = 2.3, 0.9 Hz, 1H), 7.82 (dd, J = 8.1, 2.2 Hz, 1H), 7.50 (dd, J = 8.1, 0.8 Hz, 1H), 4.88 (d, J = 2.4 Hz, 2H), 4.55 (s, 2H), 3.96 (q, J = 7.0 Hz, 1H), 3.22 (s, 3H), 1.41 (s, 6H), 1.33 (s, 3H), 1.30 (s, 9H). **^{13}C NMR** (100 MHz, MeOD) δ 184.47, 175.48, 158.69, 158.34, 148.48, 137.92, 134.67, 122.54, 101.89, 80.94, 64.24, 50.96, 49.07, 45.88, 28.62, 24.77, 17.08. **HRMS** (ESI, pos. mode) m/z calc. for $C_{20}H_{34}N_5O_5S^+$ 456.2281, found 456.2276 $[M+H]^+$.

Compound 11. To a solution of compound **10** (0.2 g) in 20 mL MeOH, 10 mL 20% NaOH was added. The resulting mixture was stirred at room temperature for 12 h. The mixture was then neutralized to pH 7 and the solvent was evaporated under reduced pressure. The residue was purified by flash chromatography (hexane/0-100% ethyl acetate) to give compound **11** as a white solid (0.14 g, 74% yield). **^{1}H NMR** (400 MHz, $CDCl_3$) δ 13.14 (s, 1H), 8.61 (s, 1H), 7.86 – 7.64 (m, 1H), 7.42 (d, J = 8.1 Hz, 1H), 5.55 – 5.48 (m, 2H), 5.18 (d, J = 15.7 Hz, 1H), 4.89 – 4.81 (m, 1H), 4.59 (s, 2H), 3.18 (s, 3H), 1.38 (s, 9H). **^{13}C NMR** (100 MHz, $CDCl_3$) δ 159.01, 155.17, 155.05, 147.53, 136.84, 129.82, 121.50, 100.83, 80.84, 63.43, 48.90, 44.58, 42.27, 31.07, 28.37, 24.60,

19.22. **HRMS** (ESI, pos. mode) m/z calc. for $C_{20}H_{32}N_5O_4S^+$ 438.2175, found 438.2170 $[M+H]^+$.

Compound 12. To a solution of compound **11** (0.2 g, 0.46 mmol) and K_2CO_3 (0.13 g, 0.94 mmol) in 20 mL DMF, a solution of 4-fluorobenzyl bromide (87 mg, 0.46 mmol in 5 mL DMF) was added. The resulting mixture was stirred at room temperature for 2 h. When the reaction was completed, the solvent was evaporated under reduced pressure. The residue was purified by flash chromatography (ethyl acetate /0-10% MeOH) to give **12** as a white solid (0.17 g, 68% yield). **^{1}H NMR** (400 MHz, $CDCl_3$) δ 8.34 (dd, J = 2.3, 0.8 Hz, 1H), 7.38 (dd, J = 8.1, 0.8 Hz, 1H), 7.32 – 7.23 (m, 3H), 7.19 (dd, J = 8.1, 2.3 Hz, 1H), 7.04 – 6.84 (m, 2H), 5.05 (d, J = 2.3 Hz, 2H), 4.90 (m, 2H), 4.58 (s, 2H), 4.37 (s, 2H), 3.22 (s, 3H), 1.54 (d, J = 6.7 Hz, 3H), 1.42 (s, 6H), 1.36 (s, 9H). **^{13}C NMR** (100 MHz, $CDCl_3$) δ 163.49, 161.04, 159.39, 157.04, 154.98, 150.73, 147.51, 134.77, 132.48, 130.82, 130.74, 128.92, 121.03, 115.65, 100.54, 80.18, 63.60, 48.66, 44.40, 41.58, 37.36, 28.20, 24.45, 19.72. **HRMS** (ESI, pos. mode) m/z calc. for $C_{27}H_{37}FN_5O_4S^+$ 546.2550, found 546.2545 $[M+H]^+$.

Compound 13. Compound **12** (0.1 g, 0.18 mmol) was dissolved in CH_2Cl_2/TFA (20%). The resulting mixture was stirred at room temperature for 1 h. Then the solvent was removed by vacuum and the residue was further purified by RP-HPLC (0.1% TFA in H_2O/acetonitrile 15 - 90%, 50 min) to give compound **13** as a yellow oil. Retention time 25 min, yield 90%. **^{1}H NMR** (400 MHz, CD_3CN) δ 8.42 (s, 1H), 7.73 (dd, J = 8.3, 2.0 Hz, 1H), 7.58 (d, J = 8.3 Hz, 1H), 7.36 – 7.20 (m, 2H), 7.06 – 6.93 (m, 2H), 5.39 – 5.05 (m, 2H), 4.78 (s, 2H), 4.70 (m, 1H), 4.32 (s, 2H), 1.53 (d, J = 6.7 Hz, 3H). **^{13}C NMR** (100 MHz, CD_3CN) δ 164.39, 161.96, 159.89, 155.00, 152.70, 144.10, 141.15, 134.15, 132.05, 131.96, 123.83, 116.44, 116.22, 62.76, 45.14, 44.12, 38.01, 19.77. **HRMS** (ESI, pos. mode) m/z calc. for $C_{18}H_{21}FN_5OS^+$ 374.1451, found 374.1445 $[M+H]^+$.

Compound 14. To a solution of 2-(trifluoromethyl)benzoic acid (95 mg, 0.5 mmol), HOBt (81 mg, 0.6 mmol) and DIPEA (0.26 mL, 1.5 mmol) in 15 mL dry DMF, EDC·HCl (115 mg, 0.6 mmol) was added. The resulting mixture was stirred at room temperature for 30 min, and then compound **13** (0.19 g, 0.5 mmol) was added. After stirring at room temperature overnight, brine (50 mL) was added carefully and the resulting mixture was extracted with ethyl acetate (3 × 30 mL). The organic phase was washed with brine (3 × 50 mL), dried over Na_2SO_4, and concentrated under reduced pressure. The residue was purified by flash chromatography (ethyl acetate /0-10% MeOH) to give compound **14** as a white solid (242 mg, 89% yield). **^{1}H NMR** (400 MHz, DMSO-d_6) δ 9.17 (d, J = 8.3 Hz, 1H), 8.24 (s, 1H), 7.82 – 7.72 (m, 1H), 7.63 – 7.57 (m, 2H), 7.39– 7.32 (m, 4H), 7.13 – 7.07 (m, 2H), 7.00 – 6.95 (m, 1H), 5.44 (t, J = 5.8 Hz, 1H), 5.34 (dd, J = 8.1, 6.7 Hz, 1H), 5.26 – 5.05 (m, 2H), 4.54 (d, J = 5.8 Hz, 2H), 4.35 (s, 2H), 1.53 (d, J = 6.8 Hz, 3H). **^{13}C NMR** (100 MHz, DMSO-d_6) δ 166.59, 162.70, 161.67, 160.28, 156.19, 149.91, 146.79, 135.34, 134.75, 133.46, 132.18, 131.00, 129.91, 129.19, 128.26, 126.23, 125.99, 125.68, 125.05, 120.09, 115.34, 63.98, 43.93, 35.98, 18.33. **HRMS** (ESI, pos. mode) m/z calc. for $C_{26}H_{24}F_4N_5O_2S^+$ 546.1587, found 546.1581 $[M+H]^+$.

Compound 15. To a solution of compound **14** (5.5 mg, 10 μmol) in 2 mL CH_2Cl_2, pyridine (2.4 μL, 30 μmol) and 4-nitrophenyl chloroformate (6.0 mg, 30 μmol) was added. After stirring at room temperature for 2 h, the mixture was gradually added to a solution of 1,2-bis(2-aminoethoxy)ethane (30 μL, 200 μmol) in 200 μL dry CH_2Cl_2. After stirring for 15 minutes at room temperature, the reaction mixture was concentrated under reduced pressure. The residue was acidified by addition of 100 μL H_2O and 200 μL acetic acid. The product was purified by RP-HPLC (0.1% TFA in H_2O/acetonitrile 10 - 90%, 50 min) and lyophilized to give compound **15** as a white solid. Retention time 27.3 min, yield 65%. **^{1}H NMR** (400 MHz, DMSO-d_6) δ 9.19 (d, J = 8.3 Hz, 1H), 8.29 (d, J = 2.3 Hz, 1H), 7.82 – 7.70 (m, 4H), 7.65 – 7.55 (m, 2H), 7.48 – 7.38 (m, 2H), 7.37 – 7.26 (m, 3H), 7.09 (t, J = 8.9 Hz, 2H), 6.99 (m, 1H), 5.45 – 5.28 (m, 1H), 5.26 – 5.11 (m, 2H), 5.07 (s, 2H), 4.35 (s, 2H), 3.59 – 3.54 (m, 6H), 3.43 (t, J = 6.0 Hz, 2H), 3.17 (q, J = 6.0 Hz, 2H), 2.97 (h, J = 5.6 Hz, 2H), 1.53 (d, J = 6.9 Hz, 3H). **^{13}C NMR** (100 MHz, DMSO-d_6) δ 166.73, 162.78, 160.36, 156.42, 156.32, 156.03, 150.00, 147.22, 135.36, 135.25, 133.45, 132.27, 131.06, 130.34, 130.00, 128.30, 126.37, 126.06, 125.11, 122.39, 121.21, 115.40, 69.70, 69.46, 69.16, 66.72, 65.62, 43.93, 40.25, 36.09, 18.38. **HRMS** (ESI, pos. mode) m/z calc. for $C_{33}H_{39}F_4N_7O_5S^{2+}$ 360.6335, found 360.6329 $[M+2H]^{2+}$.

Compound 16. Compound **16** was prepared according to similar procedure for compound **15**. The product was purified by RP-HPLC (0.1% TFA in H_2O/acetonitrile 10 - 90%, 50 min) and lyophilized to give compound **16** as a white solid. Retention time 27.3

min, yield 85%. **^{1}H NMR** (400 MHz, DMSO-d_6) δ 9.19 (d, J = 8.3 Hz, 1H), 8.30 (d, J = 2.3 Hz, 1H), 7.74 (m, 4H), 7.66 – 7.55 (m, 2H), 7.49 – 7.24 (m, 5H), 7.09 (t, J = 8.9 Hz, 2H), 6.97 (dd, J = 5.4, 3.5 Hz, 1H), 5.39 – 5.30 (m, 1H), 5.19 (q, J = 17.1 Hz, 2H), 5.07 (s, 2H), 4.35 (s, 2H), 3.00 (q, J = 6.6 Hz, 2H), 2.85 – 2.67 (m, 2H), 1.52 (m, 5H), 1.41 (t, J = 6.9 Hz, 2H), 1.32 – 1.19 (m, 4H). **^{13}C NMR** (100 MHz, DMSO-d_6) δ 166.66, 162.73, 160.31, 156.52, 155.86, 149.94, 147.19, 135.33, 135.15, 133.39, 132.23, 131.02, 130.25, 128.26, 126.21, 126.01, 125.70, 121.10, 117.40, 115.36, 65.46, 43.89, 40.23, 38.79, 36.03, 29.19, 26.96, 25.74, 25.47, 18.35. **HRMS** (ESI, pos. mode) m/z calc. for $C_{33}H_{38}N_7O_3F_4S^+$ 688.2693, found 688.2690 $[M+H]^+$.

TMR-TAZ. To a 50 mM solution of tetramethylrhodamine (TMR) in DMSO (200 µL), and DIPEA (5.3 µL, 30 µmol), a 50 mM solution of TSTU in dry DMSO (200 µL) was added. After stirring for 10 minutes at room temperature, the reaction was gradually added to a 200 µL DMSO solution of compound **15** (7.2 mg, 10 µmol). After stirring 15 minutes at room temperature, the reaction was quenched by addition of 100 µL H_2O and 30 µL acetic acid. The product was purified by RP-HPLC (0.1% TFA in H_2O/acetonitrile 20 - 90%, 45 min) and lyophilized to give compound **TMR-TAZ** as a purple solid. Retention time 24.2 min, yield 72 %. **^{1}H NMR** (400 MHz, DMSO-d_6) δ 9.17 (d, J = 8.4 Hz, 1H), 8.81 (t, J = 5.5 Hz, 1H), 8.38 – 8.18 (m, 3H), 7.88 (d, J = 1.7 Hz, 1H), 7.75 – 7.73 (m, 1H), 7.65 – 7.56 (m, 2H), 7.45 – 7.22 (m, 5H), 7.14 – 6.89 (m, 9H), 5.31 – 5.35 (m, 1H), 5.25 – 5.11 (m, 2H), 5.04 (s, 2H), 4.33 (s, 2H), 3.56 – 3.47 (m, 10H), 3.26 (s, 12H), 3.09 (q, J = 5.9 Hz, 2H), 1.52 (d, J = 6.8 Hz, 3H). HRMS (ESI, pos. mode) m/z calc. for $C_{58}H_{59}F_4N_9O_9S^{2+}$ 566.7044, found 566.7041 $[M+H]^{2+}$.

Compound 17. To a solution of **TMR-COOEt** (21 mg, 40 µmol) and K_2CO_3 (11 mg, 80 µmol) in DMF (400 µL), a solution of allyl bromide (14.5 mg, 120 µmol) in 200 µL DMF were added in portions. After stirring at room temperature for 3 h, the reaction was quenched by addition of 100 µL acetic acid and 200 µL H_2O. The product was purified by RP-HPLC (0.1% TFA in H_2O/acetonitrile 20 - 90%, 60 min) and lyophilized to give compound **17** as a purple solid. Retention time 32 min, yield 80%. **^{1}H NMR** (400 MHz, DMSO-d_6) 8.35 (s, 2H), 7.96 (s, 1H), 7.12 – 6.87 (m, 6H), 6.03 (m, 1H), 5.41 (dd, J = 17.2, 1.6 Hz, 1H), 5.27 (dd, J = 10.5, 1.5 Hz, 1H), 4.83 (d, J = 5.6 Hz, 2H), 4.05 (q, J = 7.1 Hz, 2H), 3.64 (t, J = 7.6 Hz, 2H), 3.26 (s, 6H), 3.22 (s, 3H), 2.41 (t, J = 7.1 Hz, 2H), 1.86 (q, J = 7.3 Hz, 2H), 1.16 (t, J = 7.1 Hz, 3H). **HRMS** (ESI, pos. mode) m/z calc. for $C_{33}H_{35}N_2O_7^+$ 571.2439, found 571.2438 $[M]^+$.

Compound 18. To a solution of **17** (5.7 mg, 10 µmol) in dry CH_2Cl_2 (2 mL), oxalyl dichloride (100 µL) and dry DMF (30 µL) were added. After stirring at room temperature for 3 h, the solvent was evaporated by vacuum. The residue was dissolved in dry CH_2Cl_2 and gradually added into a solution of methanesulfonamide (2.0 mg, 20 µmol) and DIPEA (5.3 µL, 30 µmol) in 2 mL dry DMF. After stirring at room temperature for 1 h, all the solvents were evaporated. The residue was purified by RP-HPLC (0.1% TFA in H_2O/acetonitrile 20 - 90%, 60 min) and lyophilized to give **18** as a purple solid. Retention time 39 min, yield 64%. **^{1}H NMR** (400 MHz, DMSO-d_6) δ 8.18 (dd, J = 7.9, 1.4 Hz, 1H), 8.11 (dd, J = 7.9, 1.3 Hz,1H), 7.45 (s, 1H), 6.63 (s, 2H), 6.45 (s, 4H), 5.97 (m, 1H), 5.32 (dd, J = 17.2, 1.6 Hz, 1H), 5.22 (dd, J = 10.5, 1.5 Hz, 1H), 4.74 (d, J = 5.7 Hz, 2H), 4.04 (q, J = 7.1 Hz, 2H), 3.35 (t, J = 7.4 Hz, 2H), 3.09 – 2.84 (m, 12H), 2.34 (t, J = 7.1 Hz, 2H), 1.76 (p, J = 7.3 Hz, 2H), 1.15 (t, J = 7.1 Hz, 3H). **HRMS** (ESI, pos. mode) m/z calc. for $C_{34}H_{38}N_3O_8S^+$ 648.2374, found 648.2380 $[M]^+$.

Compound 19. To a mixture of **18** (4.5 mg, 7 µmol), 1,3-dimethylbarbituric acid (3.1 mg, 21 µmol), and $Pd(PPh_3)_4$ (4 mg, 3.5 µmol), 3 mL acetonitrile was added. The resulting solution was stirred under N_2 at room temperature for 4 h. When the reaction was completed, the solvent was evaporated by vacuum. The residue was purified by RP-HPLC (0.1% TFA in H_2O/acetonitrile 10 - 90%, 60 min) and lyophilized to give compound **19** as purple solid. Retention time 31 min, yield 90%. **^{1}H NMR** (400 MHz, DMSO-d_6) δ 8.22 – 8.02 (m, 2H), 7.45 (s, 1H), 6.65 (s, 2H), 6.47 (s, 4H), 4.03 (q, J = 7.1 Hz, 2H), 3.36 (d, J = 7.9 Hz, 2H), 3.00 (s, 3H), 2.95 – 2.92 (m, 9H), 2.34 (t, J = 7.1 Hz, 2H), 1.76 (p, J = 7.3 Hz, 2H), 1.14 (t, J = 7.1 Hz, 3H). **HRMS** (ESI, pos. mode) m/z calc. for $C_{31}H_{34}N_3O_8S^+$ 608.2061, found 608.2063 $[M]^+$.

Compound 20. To a 50 mM solution of compound **19** in DMSO (200 µL), DIPEA (5.3 µL, 30 µmol) and a 50 mM solution of TSTU in 200 µL dry DMSO were added. After stirring at room temperature for 10 min, the solution was gradually added to a solution of 2-(2-((6-chlorohexyl)oxy)ethoxy)ethanamine hydrochloride (5.2 mg, 20 µmol) in 200 µL dry DMSO. After stirring at room

temperature for 15 min, the reaction was quenched by addition of 100 µL H_2O and 30 µL acetic acid. The product was purified by RP-HPLC (0.1% TFA in H_2O/acetonitrile 20 - 90%, 60 min) and lyophilized to give compound **20** as a purple solid. Retention time 39 min, yield 71%. **^{1}H NMR** (400 MHz, DMSO-d_6) δ 8.73 (t, J = 5.6 Hz, 1H), 8.20 – 7.97 (m, 2H), 7.49 (s, 1H), 6.59 (t, J = 7.8 Hz, 2H), 6.44 (s, 4H), 4.05 (q, J = 7.1 Hz, 2H), 3.58 (t, J = 6.6 Hz, 2H), 3.53 – 3.24 (m, 12H), 2.98 (s, 3H), 2.93 (d, J = 11.6 Hz, 9H), 2.34 (t, J = 7.1 Hz, 2H), 1.76 (p, J = 7.3 Hz, 2H), 1.66 (dt, J = 14.6, 6.7 Hz, 2H), 1.40 (p, J = 6.9 Hz, 2H), 1.36 – 1.29 (m, 2H), 1.28 – 1.20 (m, 2H), 1.16 (t, J = 7.1 Hz, 3H). **HRMS** (ESI, pos. mode) m/z calc. for $C_{41}H_{54}ClN_4O_9S^+$ 813.3295, found 813.3294 $[M]^+$.

Compound 21. Compound **20** (4.4 mg, 5.4 µmol) and LiOH·H_2O (0.7 mg, 16.2 µmol) was dissolved in 2 mL THF/H_2O (1:1, v/v). The resulting mixture was stirred at room temperature for 1 h. Then the mixture was further purified by RP-HPLC (0.1% TFA in H_2O/acetonitrile 10 - 90%, 60 min) and lyophilized to give **21** as a purple solid. Retention time 34.5 min, yield 20 %. **^{1}H NMR** (400 MHz, DMSO-d_6) δ 8.72 (t, J = 5.6 Hz, 1H), 8.14 – 7.93 (m, 2H), 7.48 (s, 1H), 6.57 (d, J = 7.7 Hz, 2H), 6.43 (s, 4H), 3.49 – 3.37 (m, 6H), 3.31 (m, 6H), 2.97 (s, 3H), 2.92 (m, 9H), 2.27 (t, J = 7.2 Hz, 2H), 1.73 (q, J = 7.4 Hz, 2H), 1.65 (m, 2H), 1.40 (m, 2H), 1.32 (m, 2H), 1.28 – 1.18 (m, 2H). **HRMS** (ESI, pos. mode) m/z calc. for $C_{39}H_{50}ClN_4O_9S^+$ 785.2982, found 785.2983 $[M]^+$.

Halo-MaP-TAZ. To a 50 mM solution of **21** in 200 µL dry DMSO, DIPEA (5.3 µL, 30 µmol) and a 50 mM solution of TSTU in 200 µL dry DMSO were added. After stirring at room temperature for 10 min, the mixture was gradually added to a solution of **16** (7.2 mg, 10 µmol) in 200 µL dry DMSO. After stirring at room temperature for 15 min, the reaction was quenched by addition of 100 µL H_2O and 30 µL acetic acid. The product was purified by RP-HPLC (0.1% TFA in H_2O/acetonitrile 20 - 70%, 60 min) and lyophilized to give **Halo-MaP-TAZ** as a purple solid. Retention time 47.5 min, yield 55%. **^{1}H NMR** (400 MHz, DMSO-d_6) δ 9.17 (d, J = 8.3 Hz, 1H), 8.73 (t, J = 5.6 Hz, 1H), 8.30 (d, J = 2.3 Hz, 1H), 8.06 (q, J = 8.1 Hz, 2H), 7.87 – 7.71 (m, 2H), 7.60 (dd, J = 5.7, 3.3 Hz, 2H), 7.51 – 7.40 (m, 2H), 7.38 – 7.26 (m, 4H), 7.18 – 7.03 (m, 2H), 6.94 (m, 1H), 6.58 (s, 2H), 6.43 (s, 4H), 5.39 – 5.30 (m, 2H), 5.19 (q, J = 17.2 Hz, 4H), 5.06 (s, 2H), 4.34 (s, 2H), 3.58 (t, J = 6.6 Hz, 2H), 3.45 (m, 4H), 3.39 (m, 2H), 3.30 (m, 6H), 2.95 (q, J = 12.1, 11.7 Hz, 16H), 2.10 (t, J = 7.2 Hz, 2H), 1.80 – 1.61 (m, 4H), 1.53 (d, J = 6.9 Hz, 3H), 1.45 – 1.13 (m, 16H). **HRMS** (ESI, pos. mode) m/z calc. for $C_{72}H_{86}ClF_4N_{11}O_{11}S_2^{2+}$ 727.7785, found 727.7779 $[M+H]^{2+}$.

Halo-MaP-Me (3). To a 50 mM solution of **21** in 200 µL dry DMSO, DIPEA (5.3 µL, 30 µmol) and a 50 mM solution of TSTU in 200 µL dry DMSO were added. After stirring at room temperature for 10 min, the mixture was gradually added to a solution of methylamine (5 mg, 74 µmol) in 200 µL dry DMSO. After stirring at room temperature for 15 min, the reaction was quenched by addition of 100 µL H_2O and 30 µL acetic acid. The product was purified by RP-HPLC (0.1% TFA in H_2O/acetonitrile 20 - 95%, 45 min) and lyophilized to give **Halo-MaP-Me** as a purple solid. Retention time 18 min, yield 90%. **^{1}H NMR** (400 MHz, DMSO-d_6) δ 8.725 (t, *J* = 5.61 Hz, 1H), 8.118 – 8.007 (m, 2H), 7.732 (d, *J* = 4.91 Hz, 1H), 7.477 (s, 1H), 6.580 (t, *J* = 8.61 Hz, 2H), 6.426 (d, *J* = 7.38 Hz, 4H), 3.595 (t, *J* = 6.63 Hz, 2H), 3.475 – 3.444 (m, 6H), 3.321 – 3.282 (m, 7H), 3.006 – 2.888 (m, 12H), 2.566 (d, *J* = 4.53 Hz, 3H), 2.111 (t, *J* = 7.27 Hz, 2H), 1.769 – 1.623 (m, 4H), 1.452 – 1.233 (m, 6H). **HRMS** (ESI, pos. mode) m/z calc. for $C_{40}H_{53}ClN_5O_8S^+$ 798.3298, found 798.3303 $[M]^+$.

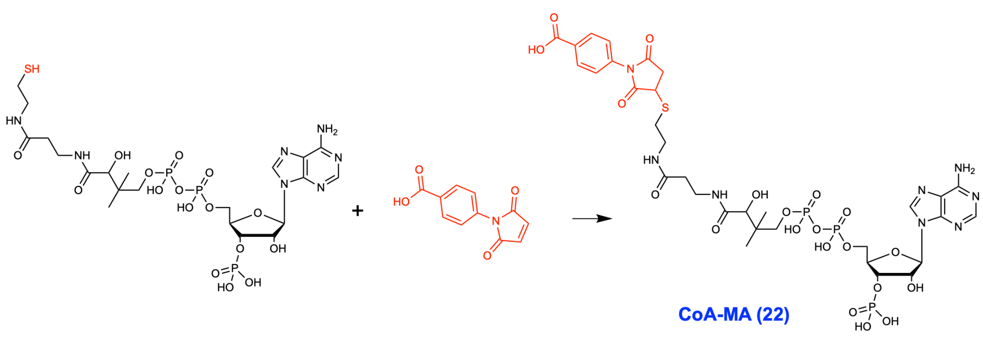

Supplementary Scheme 3. Synthesis of **CoA-MA (22)** as the internal standard for LC-MS/MS measurements.

CoA-MA. Coenzyme A trilithium salt 2-hydrate (10 mg, 12.2 µmol) were dissolved in 500 µL water and the resulting solution was

adjusted to pH 7.0 by adding NaOH (1.0 M). In the meanwhile, 10 mg of *N*-(4-carboxyphenyl)maleimide (4 mg, 18.4 mg) were dissolved in another 500 μL water and the solution was adjusted to pH 7.0 by adding NaOH (1.0 M). Subsequently the maleimide solution was gradually added to the CoA solution. After stirring at room temperature for 1h, the product was purified by RP-HPLC (0.1% TFA in H_2O/acetonitrile 0 - 50%, 60 min) and lyophilized to give **CoA-MA** as a white solid. Retention time 27 min, yield 46%. **HRMS** (ESI, pos. mode) m/z calc. for $C_{32}H_{44}N_8O_{20}P_3S^+$ 985.1605, found 985.1607 $[M+H]^+$.

NMR spectra

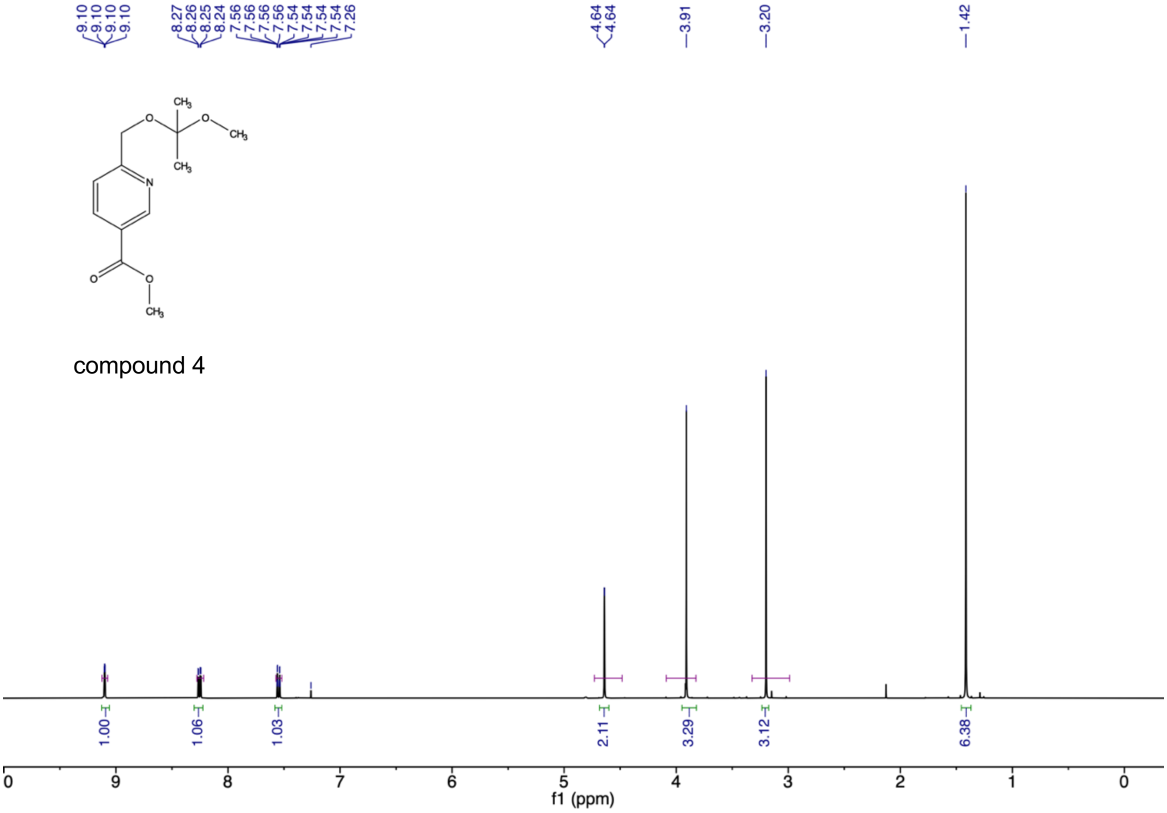

compound 4

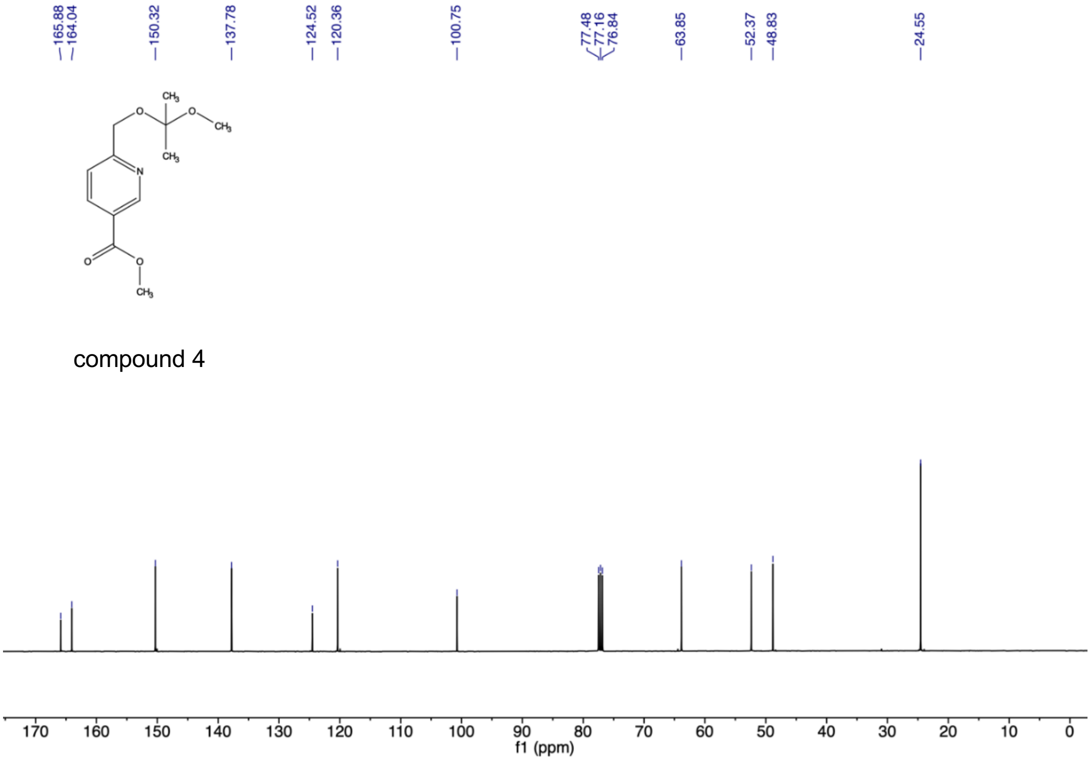

compound 4

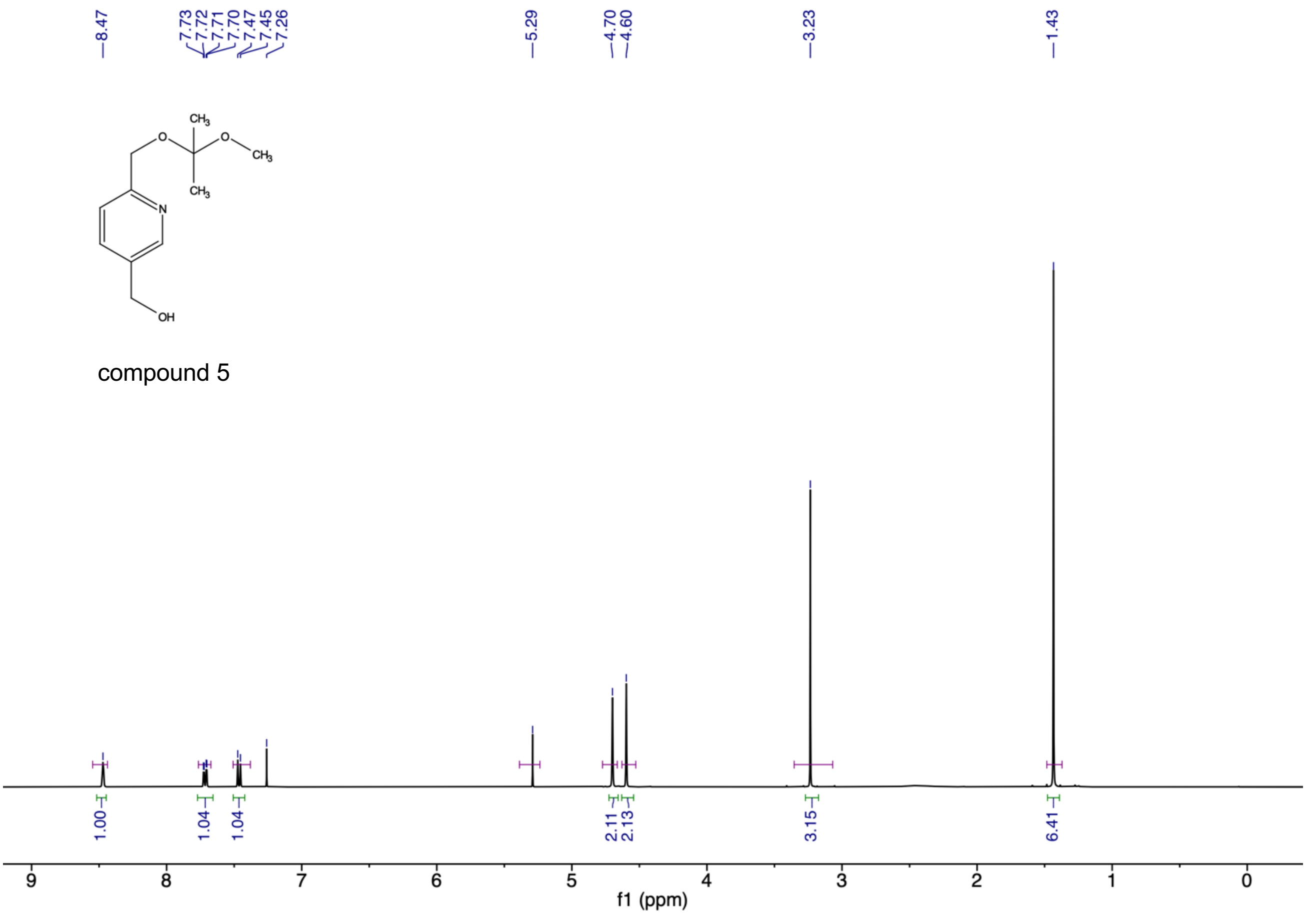
8.47
7.73
7.72
7.71
7.70
7.47
7.45
7.26
5.29
4.70
4.60
3.23
1.43
compound 5
1.00
1.04
1.04
2.11
2.13
3.15
6.41
f1 (ppm)

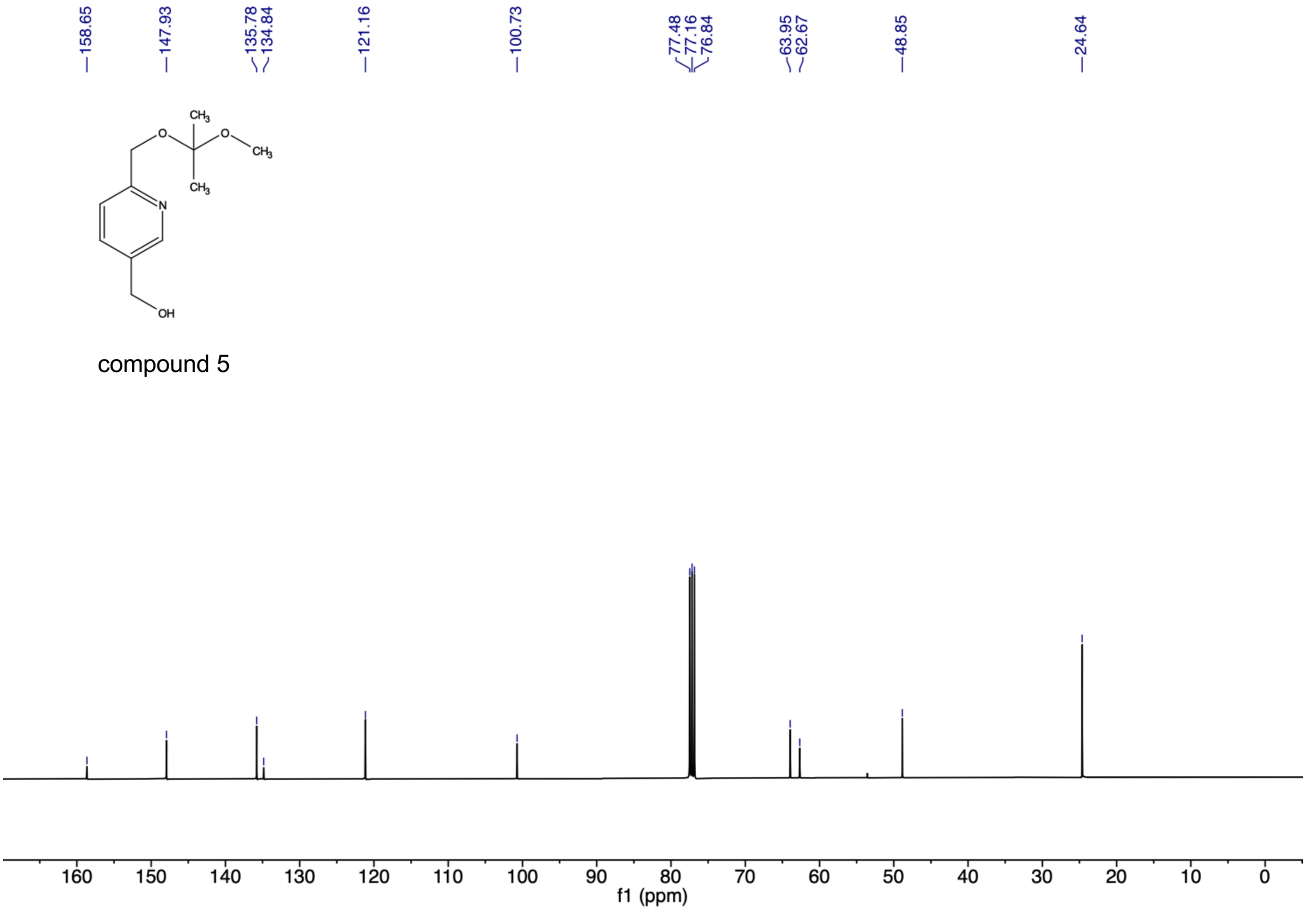
158.65
147.93
135.78
134.84
121.16
100.73
77.48
77.16
76.84
63.95
62.67
48.85
24.64
compound 5
f1 (ppm)

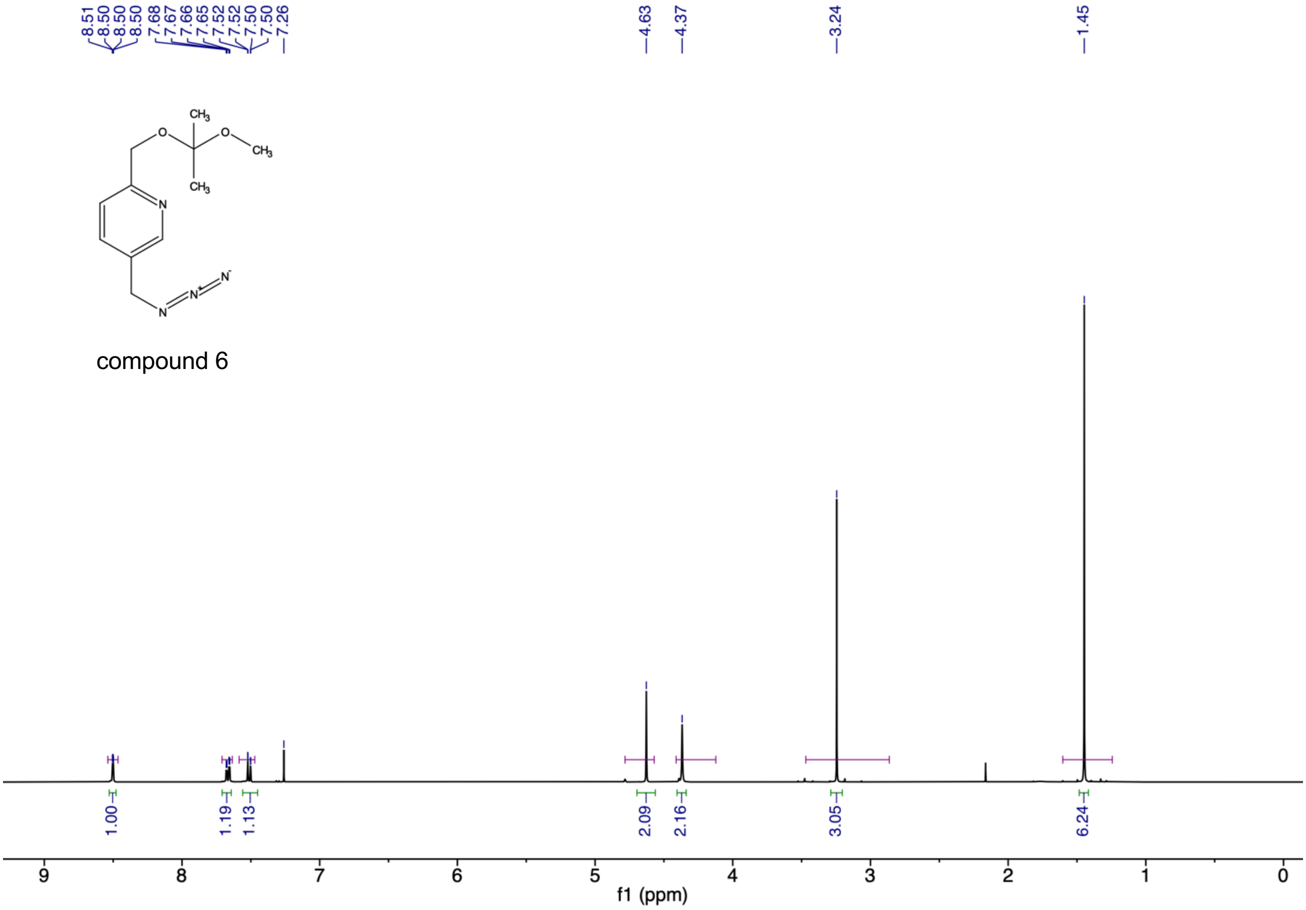
8.51 8.50 8.50 8.50 7.68 7.67 7.66 7.65 7.52 7.52 7.50 7.50 7.26
4.63 4.37
3.24
1.45
compound 6
1.00 1.19 1.13 2.09 2.16 3.05 6.24
f1 (ppm)

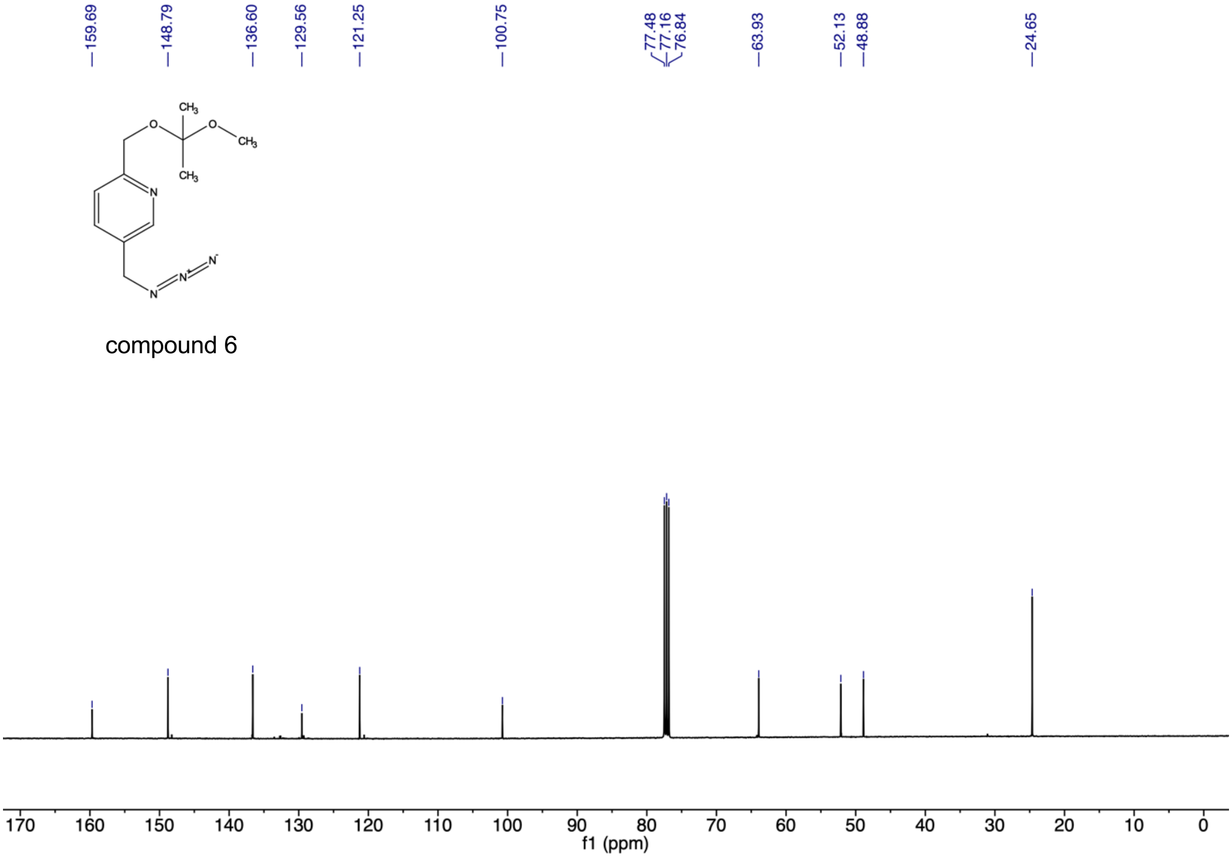
159.69 148.79 136.60 129.56 121.25 100.75 77.48 77.16 76.84 63.93 52.13 48.88 24.65
compound 6
f1 (ppm)

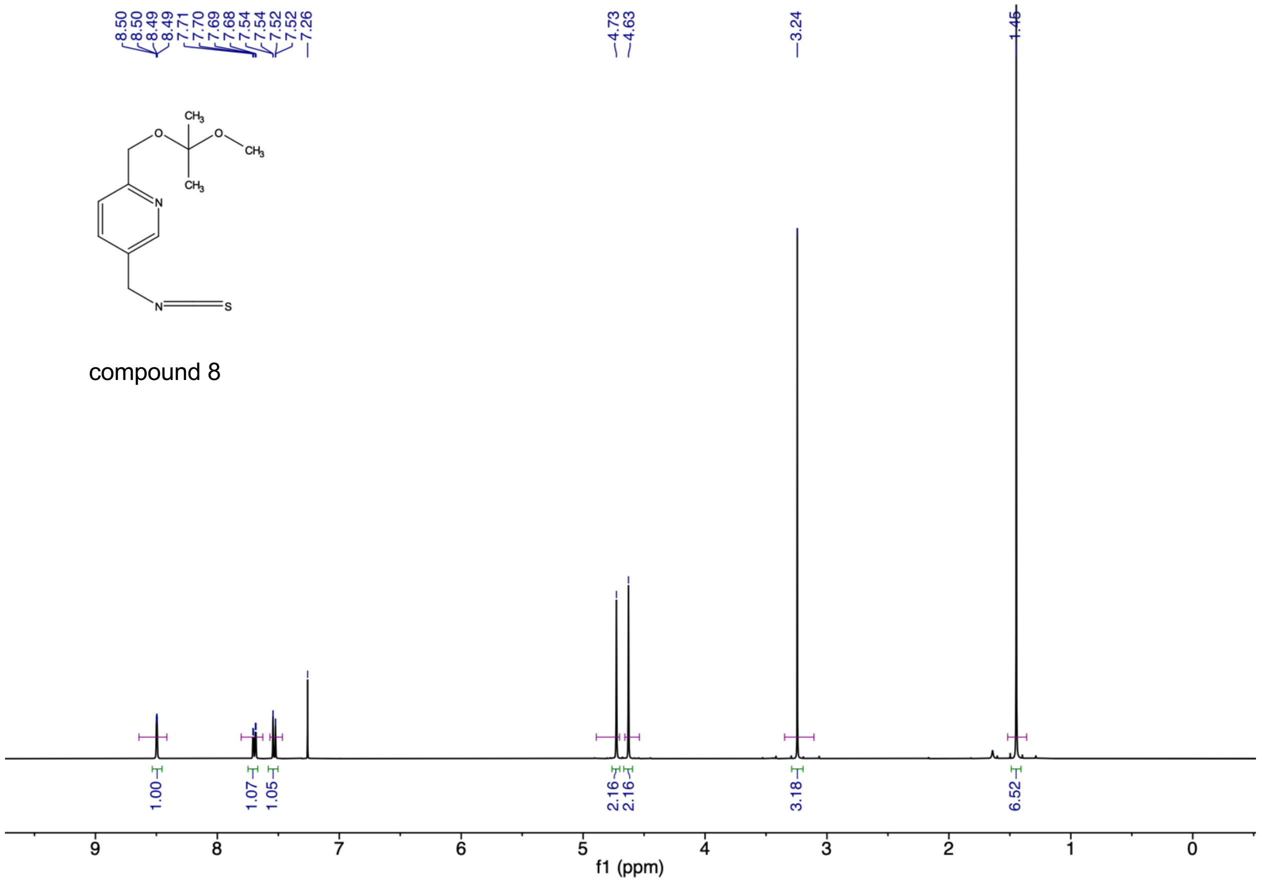

compound 8

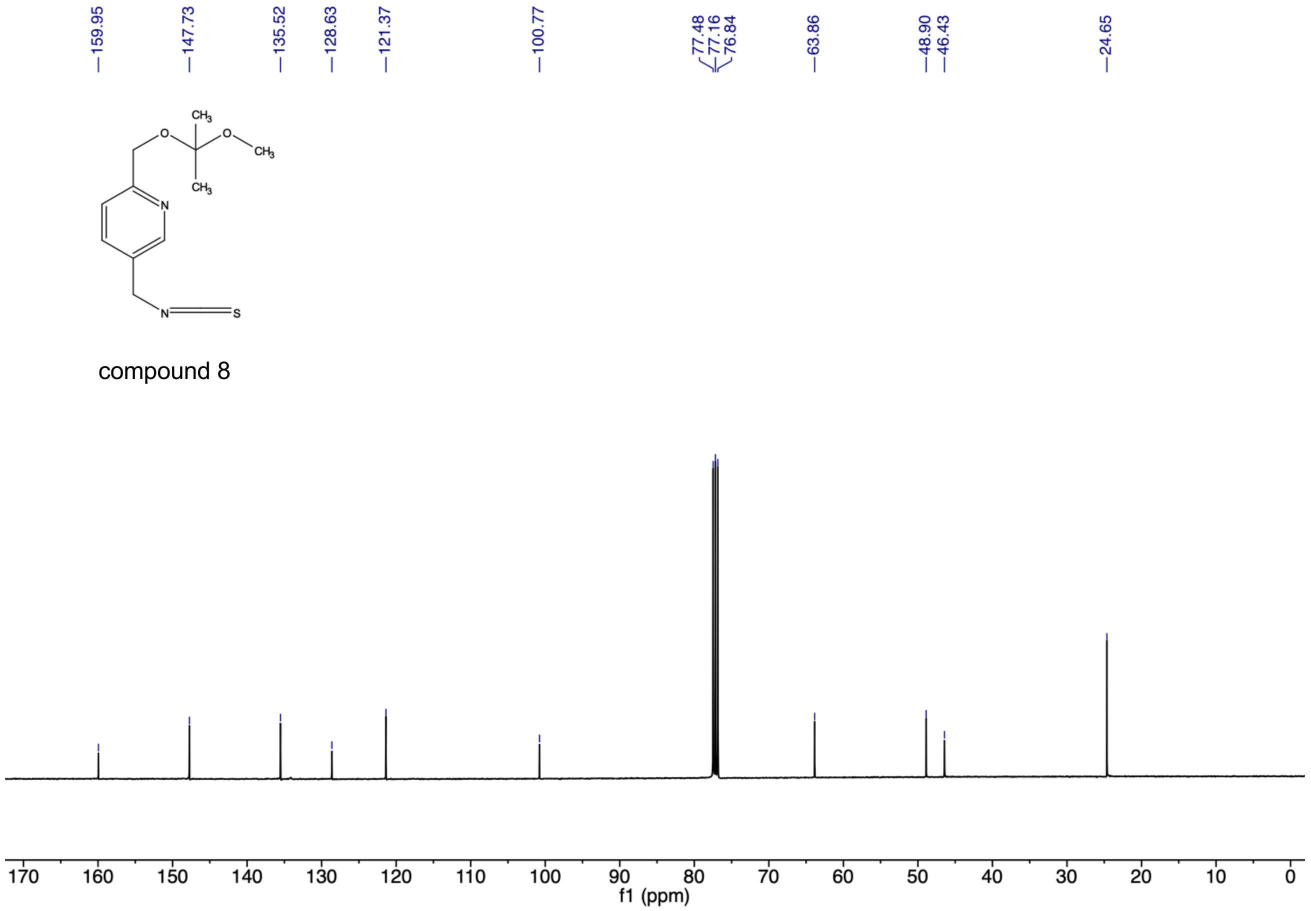

compound 8

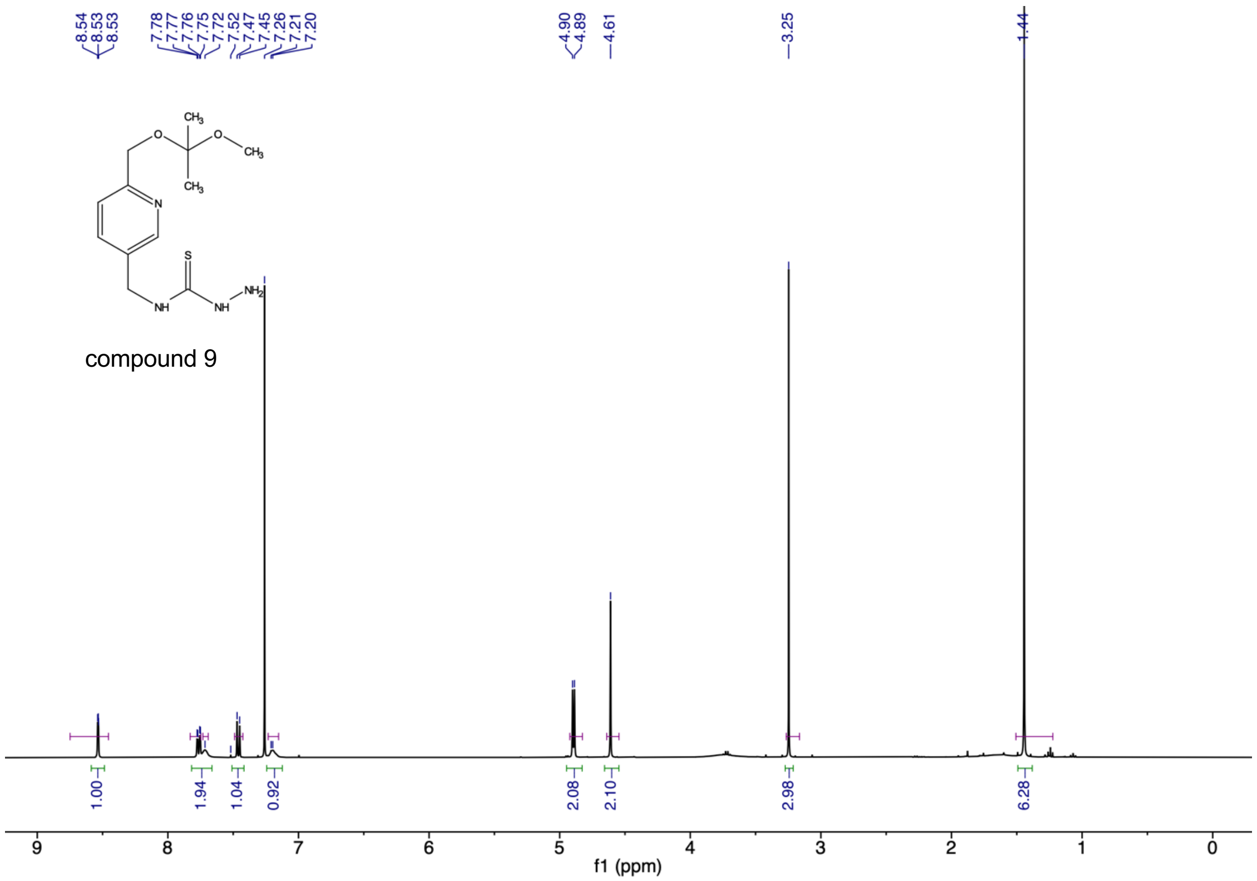
8.54
8.53
8.53
7.78
7.77
7.76
7.75
7.72
7.52
7.47
7.45
7.26
7.21
7.20
4.90
4.89
4.61
3.25
1.44
compound 9
1.00
1.94
1.04
0.92
2.08
2.10
2.98
6.28
f1 (ppm)

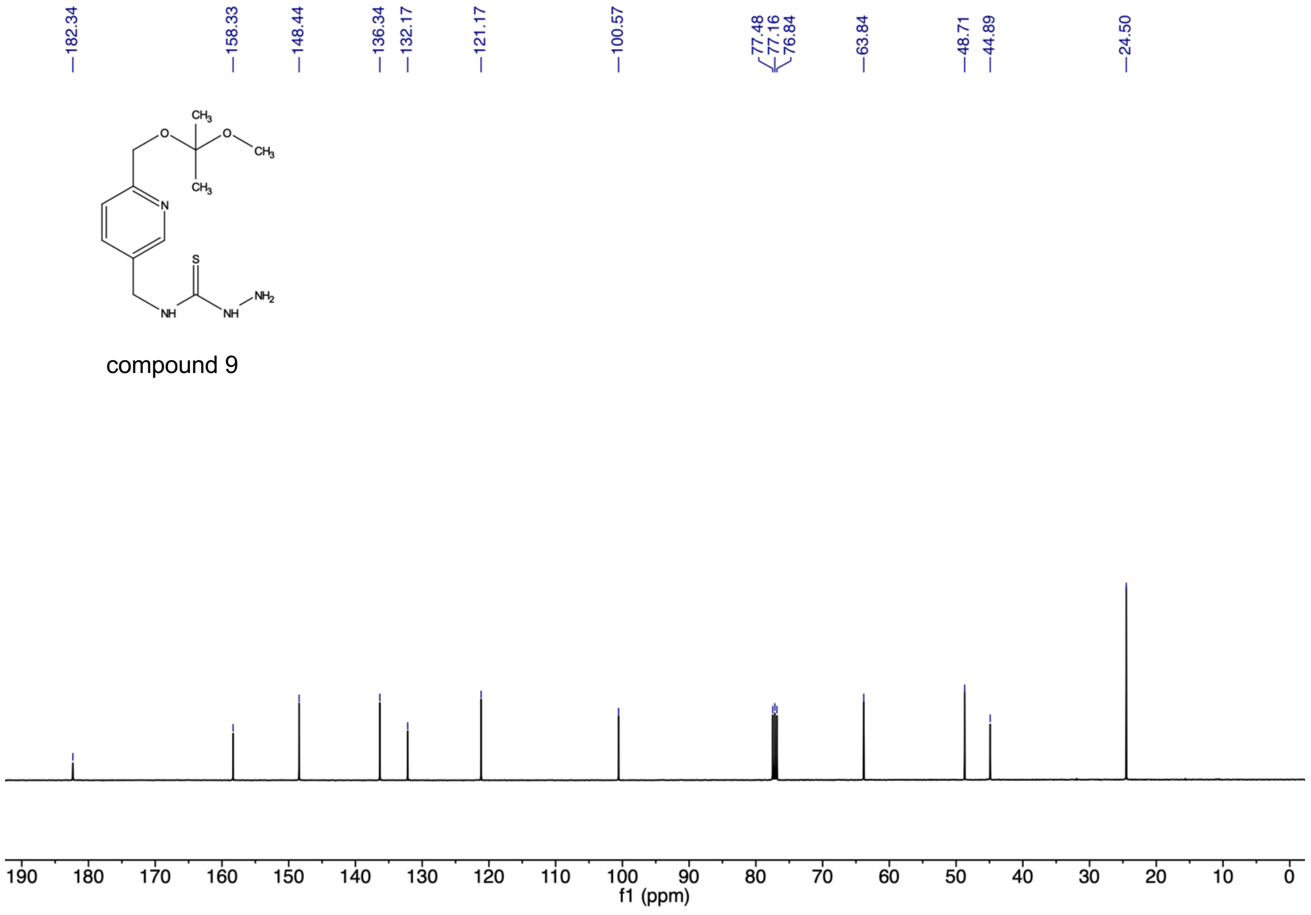
182.34
158.33
148.44
136.34
132.17
121.17
100.57
77.48
77.16
76.84
63.84
48.71
44.89
24.50
compound 9
f1 (ppm)

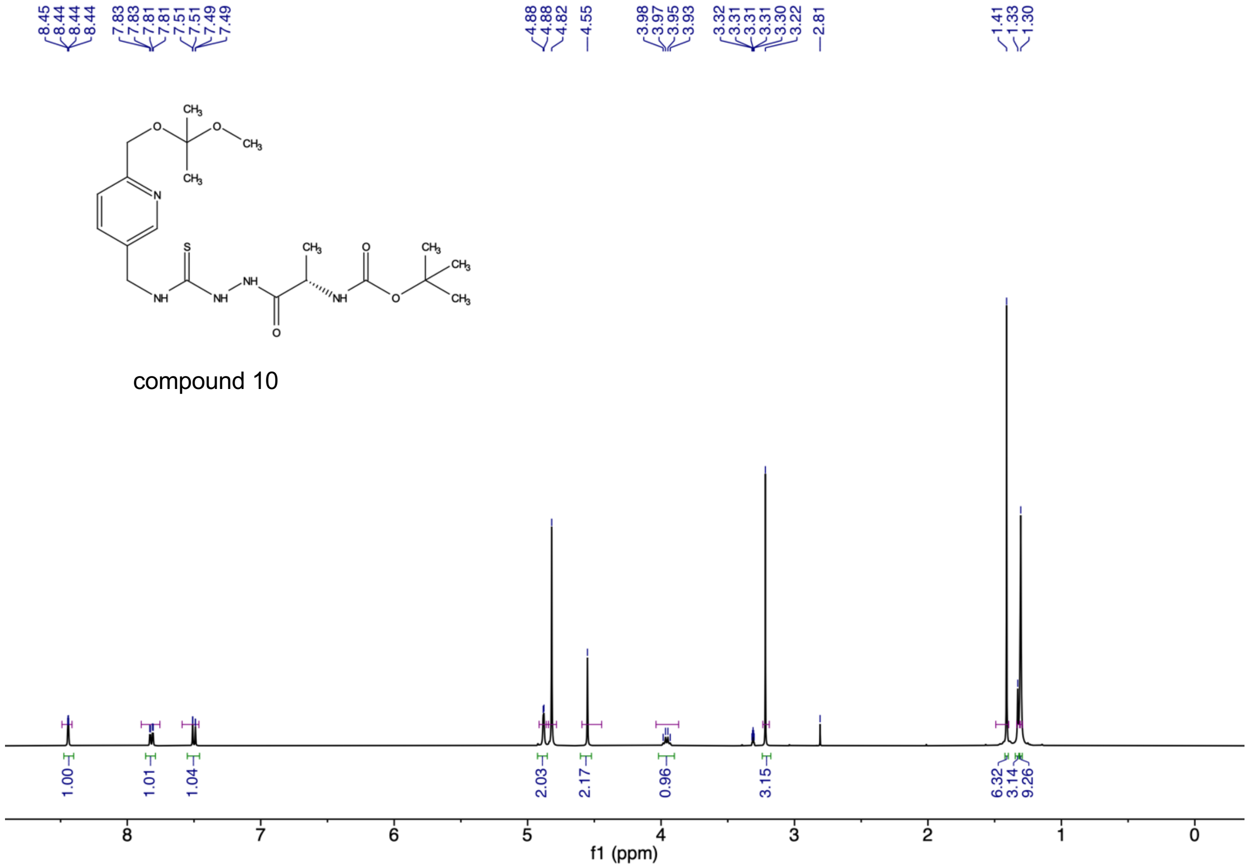
8.45 8.44 8.44 8.44 7.83 7.83 7.81 7.81 7.51 7.51 7.49 7.49
4.88 4.88 4.82 4.55
3.98 3.97 3.95 3.93 3.32 3.31 3.31 3.31 3.30 3.22 2.81
1.41 1.33 1.30
compound 10
1.00 1.01 1.04 2.03 2.17 0.96 3.15 6.32 3.14 9.26
f1 (ppm)

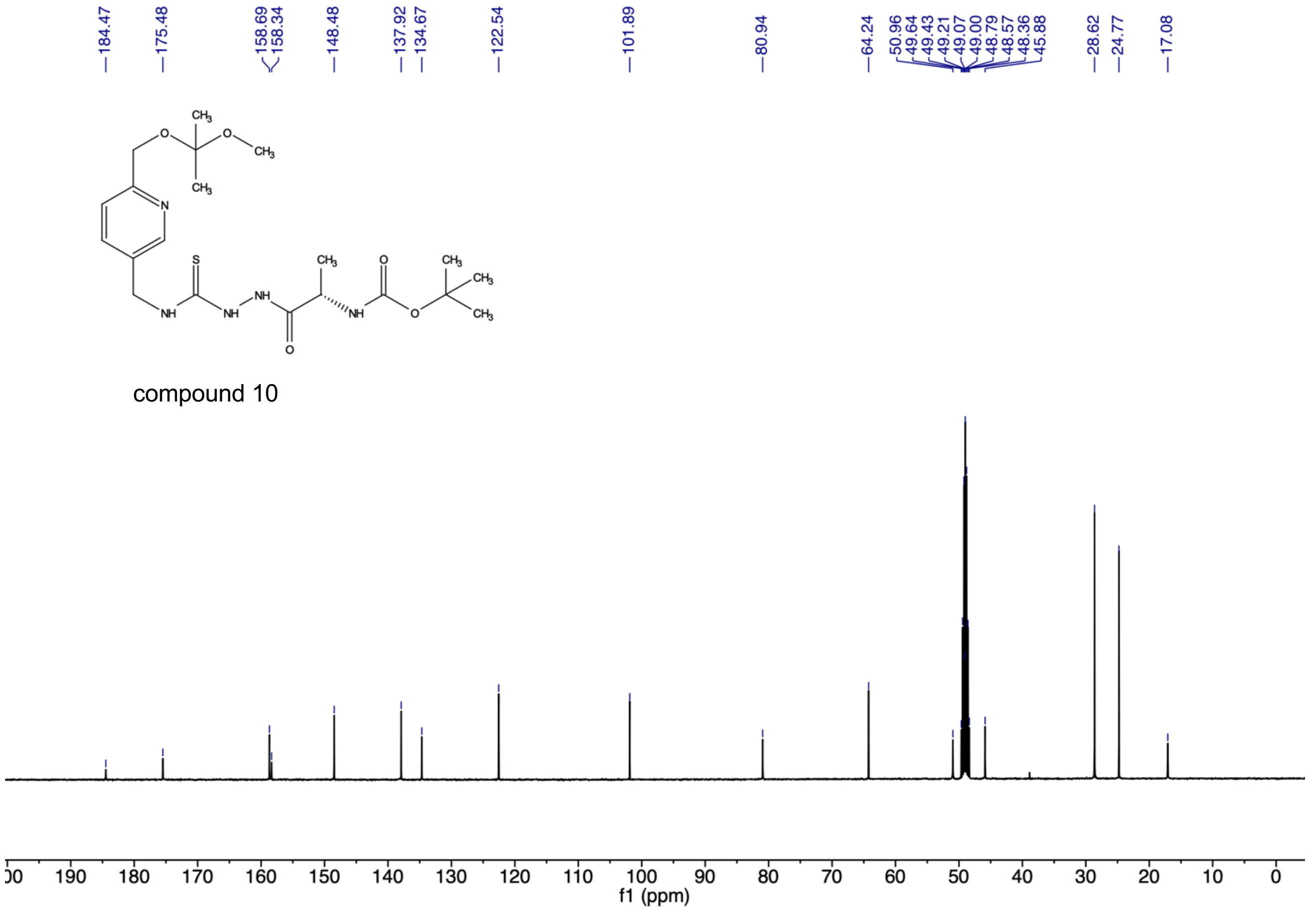
184.47 175.48 158.69 158.34 148.48 137.92 134.67 122.54 101.89 80.94
64.24 50.96 49.64 49.43 49.21 49.07 49.00 48.79 48.57 48.36 45.88 28.62 24.77 17.08
compound 10
f1 (ppm)

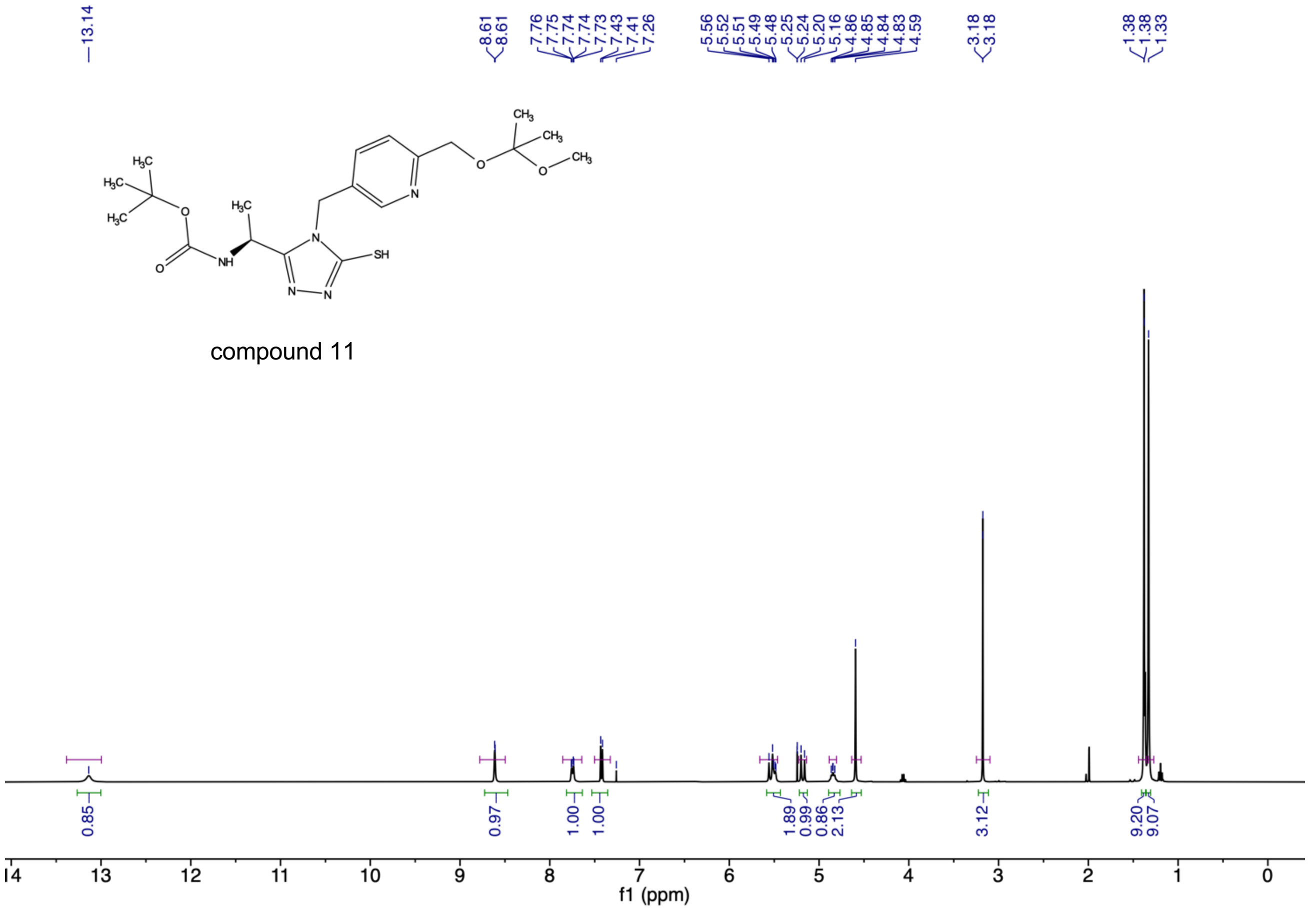
13.14
8.61 8.61
7.76 7.75 7.74 7.74 7.73 7.43 7.41 7.26
5.56 5.52 5.51 5.49 5.48 5.25 5.24 5.20 5.16 4.86 4.85 4.84 4.83 4.59
3.18 3.18
1.38 1.38 1.33
compound 11
0.85 0.97 1.00 1.00 1.89 0.99 0.86 2.13 3.12 9.20 9.07
f1 (ppm)

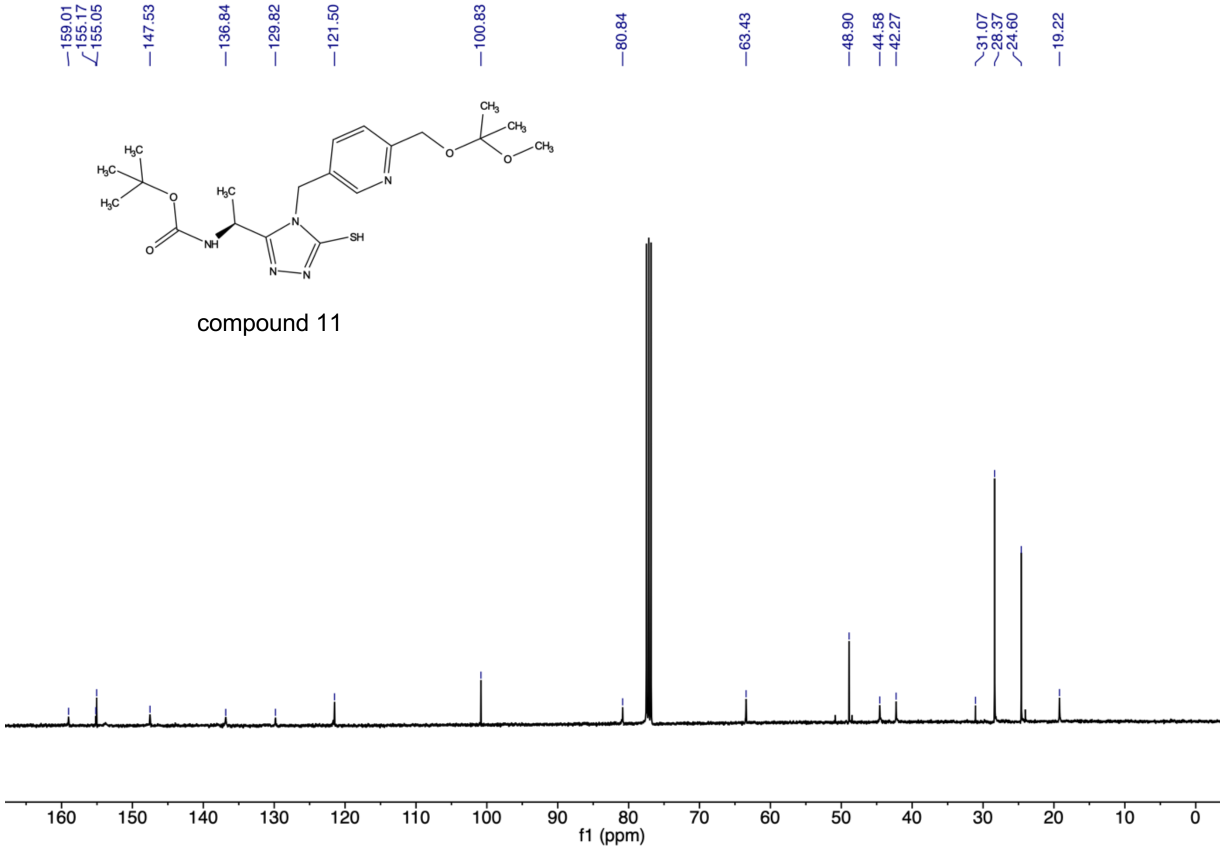
159.01 155.17 155.05 147.53 136.84 129.82 121.50 100.83 80.84 63.43 48.90 44.58 42.27 31.07 28.37 24.60 19.22
compound 11
f1 (ppm)

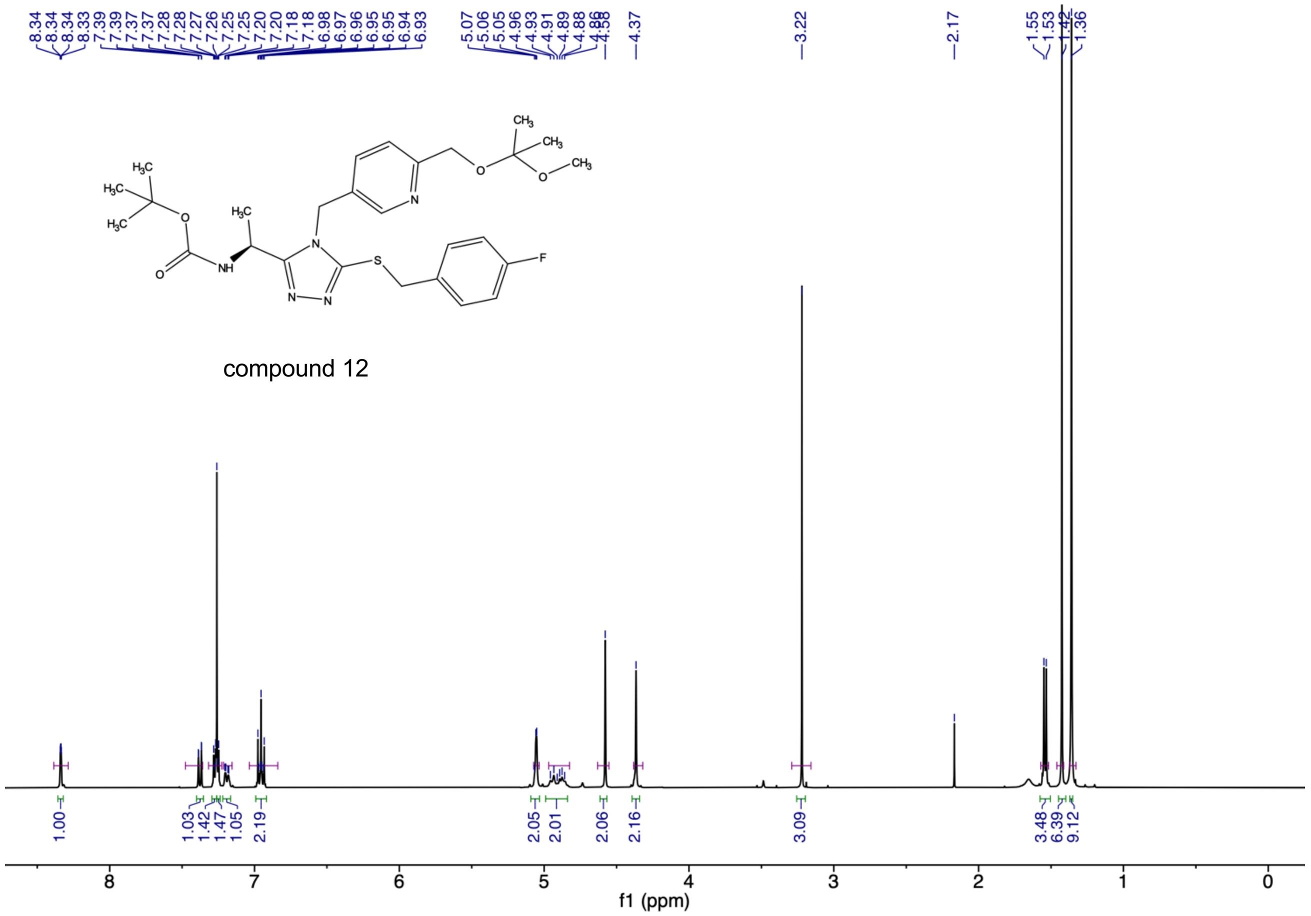
8.34 8.34 8.34 8.33 7.39 7.39 7.37 7.37 7.28 7.28 7.27 7.26 7.25 7.25 7.20 7.20 7.18 7.18 6.98 6.97 6.96 6.95 6.94 6.94 6.93 5.07 5.06 5.05 4.96 4.93 4.91 4.89 4.88 4.86 4.58 4.37 3.22 2.17 1.55 1.53 1.42 1.36
compound 12
1.00 1.03 1.42 1.47 1.05 2.19 2.05 2.01 2.06 2.16 3.09 3.48 6.39 9.12
f1 (ppm)

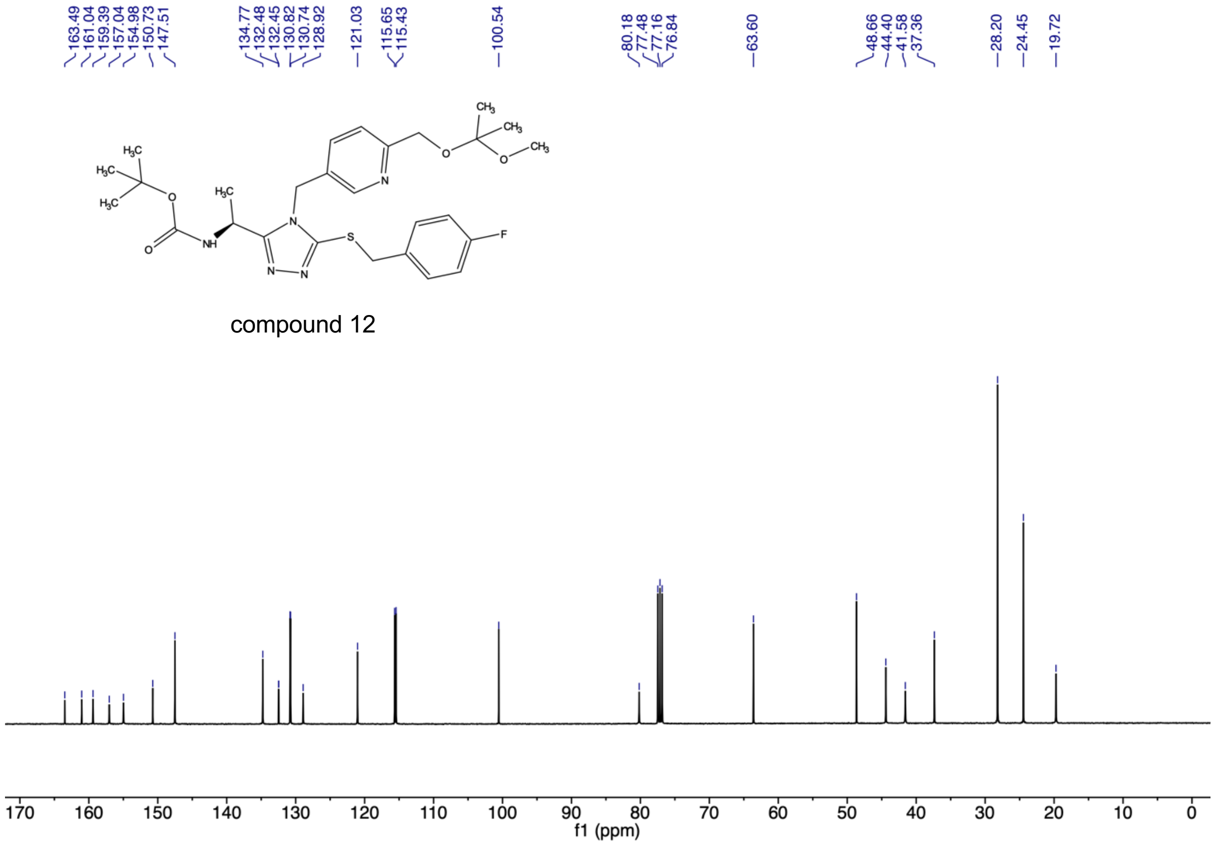
163.49 161.04 159.39 157.04 154.98 150.73 147.51 134.77 132.48 132.45 130.82 130.74 128.92 121.03 115.65 115.43 100.54 80.18 77.48 77.16 76.84 63.60 48.66 44.40 41.58 37.36 28.20 24.45 19.72
compound 12
f1 (ppm)

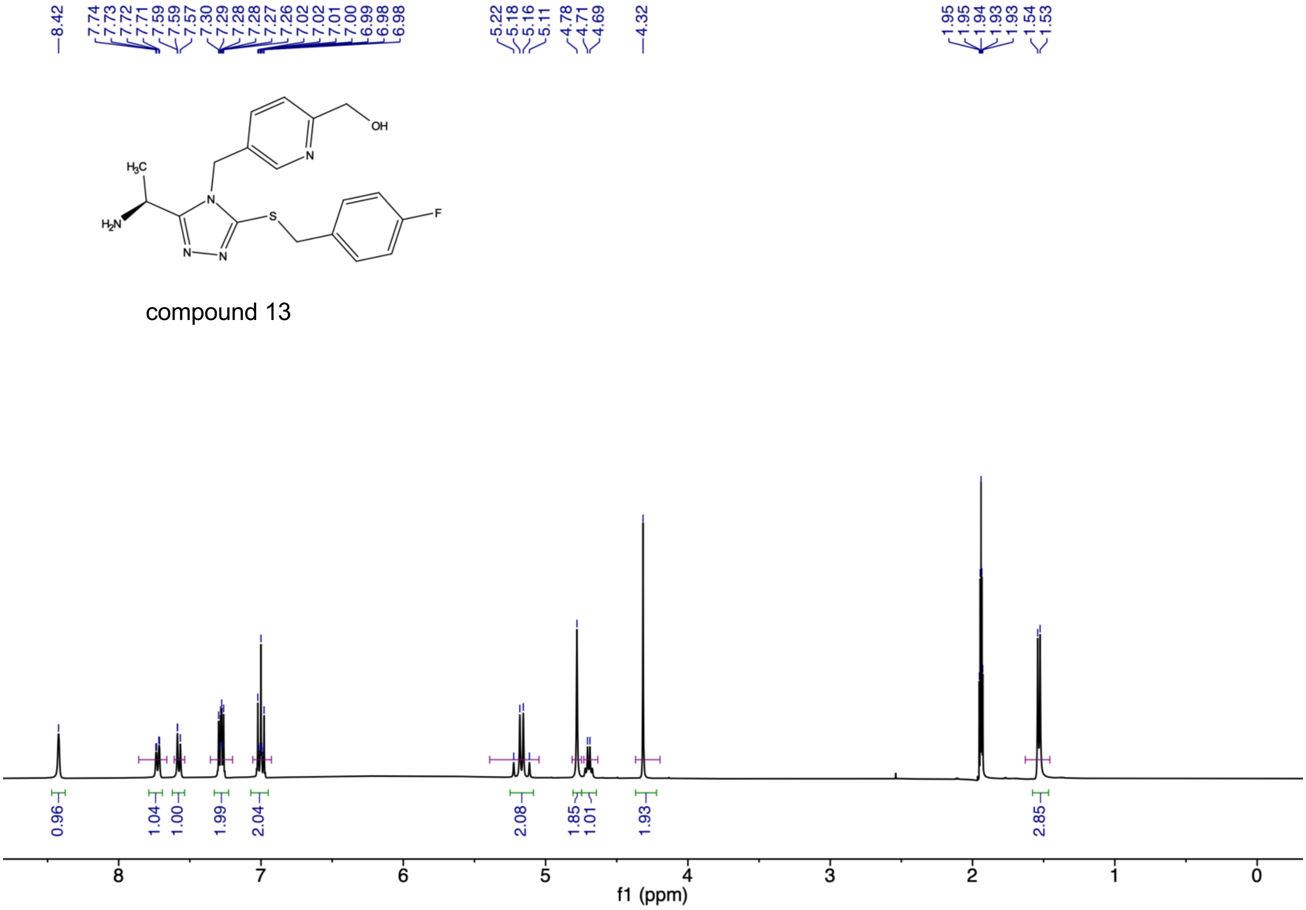
8.42
7.74 7.73 7.72 7.71 7.59 7.59 7.57 7.30 7.29 7.28 7.28 7.27 7.26 7.02 7.02 7.01 7.00 6.99 6.98 6.98
5.22 5.18 5.16 5.11 4.78 4.71 4.69
4.32
1.95 1.95 1.94 1.93 1.93 1.54 1.53
compound 13
0.96 1.04 1.00 1.99 2.04 2.08 1.85 1.01 1.93 2.85
f1 (ppm)

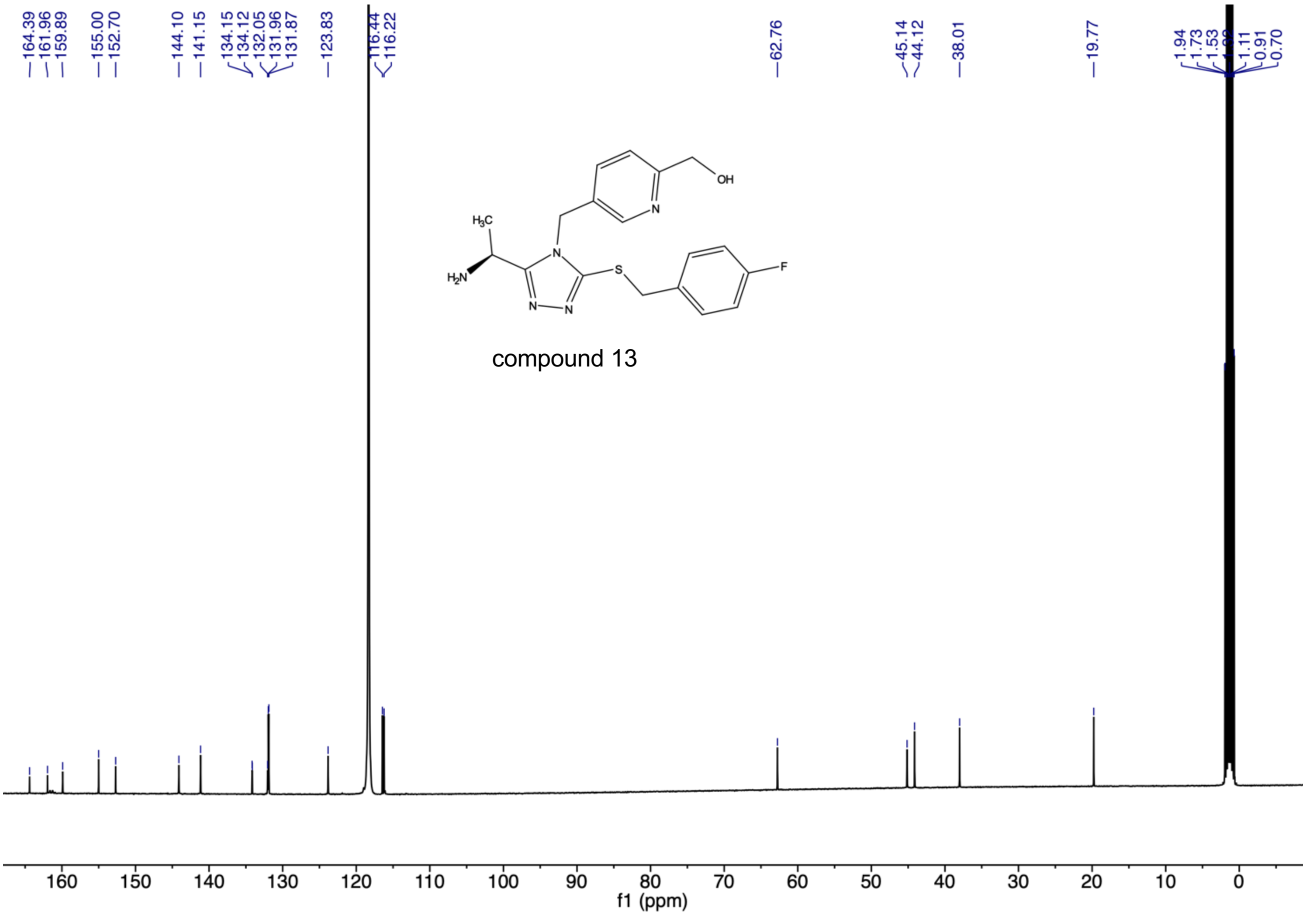
164.39 161.96 159.89 155.00 152.70 144.10 141.15 134.15 134.12 132.05 131.96 131.87 123.83 116.44 116.22
62.76
45.14 44.12 38.01
19.77
1.94 1.73 1.53 1.11 0.91 0.70
compound 13
f1 (ppm)

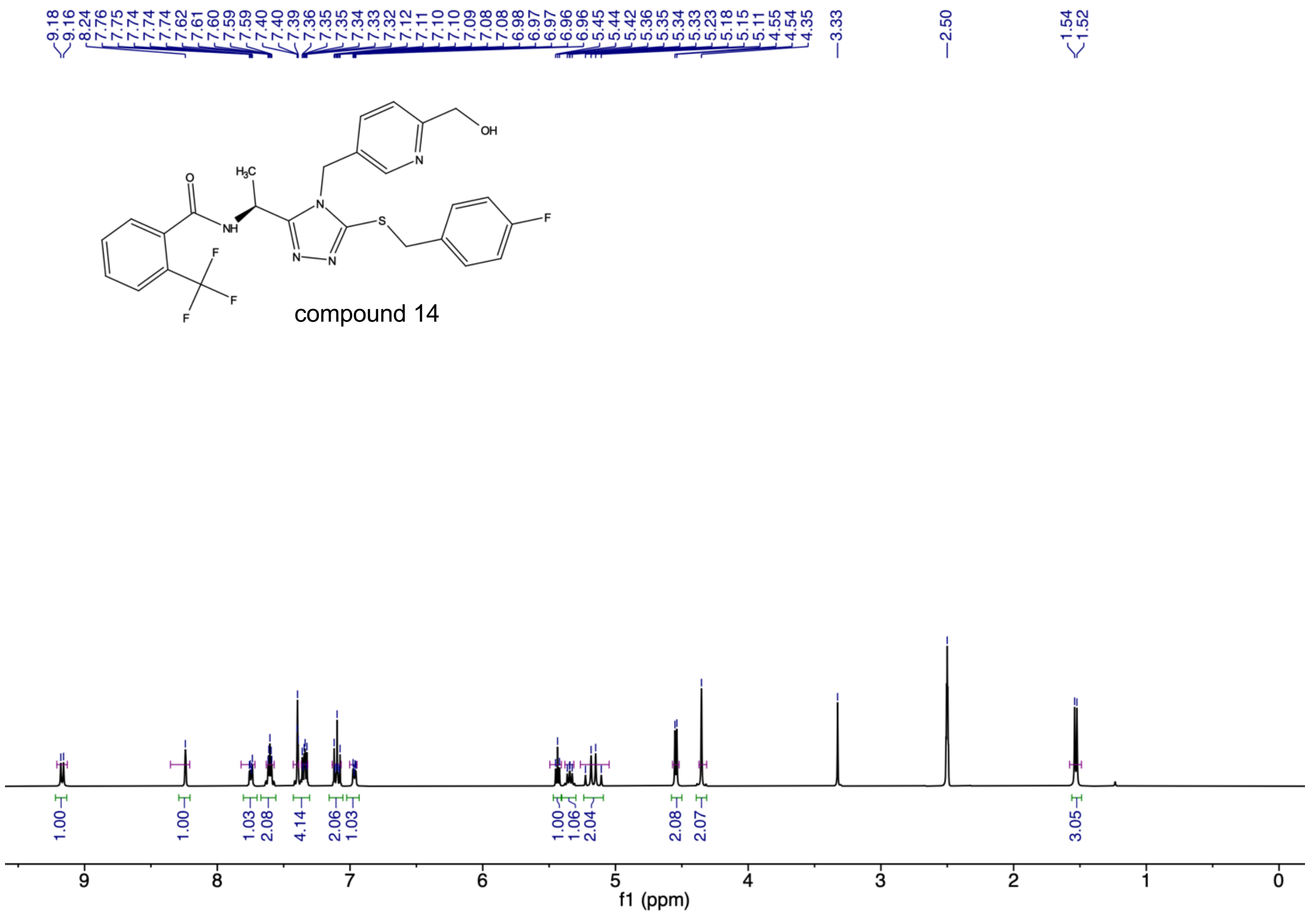
9.18
9.16
8.24
7.76
7.75
7.74
7.74
7.74
7.62
7.61
7.60
7.59
7.59
7.40
7.40
7.39
7.36
7.35
7.35
7.34
7.33
7.32
7.12
7.11
7.10
7.10
7.09
7.08
7.08
6.98
6.97
6.97
6.96
6.96
5.45
5.44
5.42
5.36
5.35
5.34
5.33
5.23
5.18
5.15
5.11
4.55
4.54
4.35
3.33
2.50
1.54
1.52
OH
N
H_3C
O
NH
N
S
F
N
N
F
F
F
compound 14
1.00
1.00
1.03
2.08
4.14
2.06
1.03
1.00
1.06
2.04
2.08
2.07
3.05
f1 (ppm)

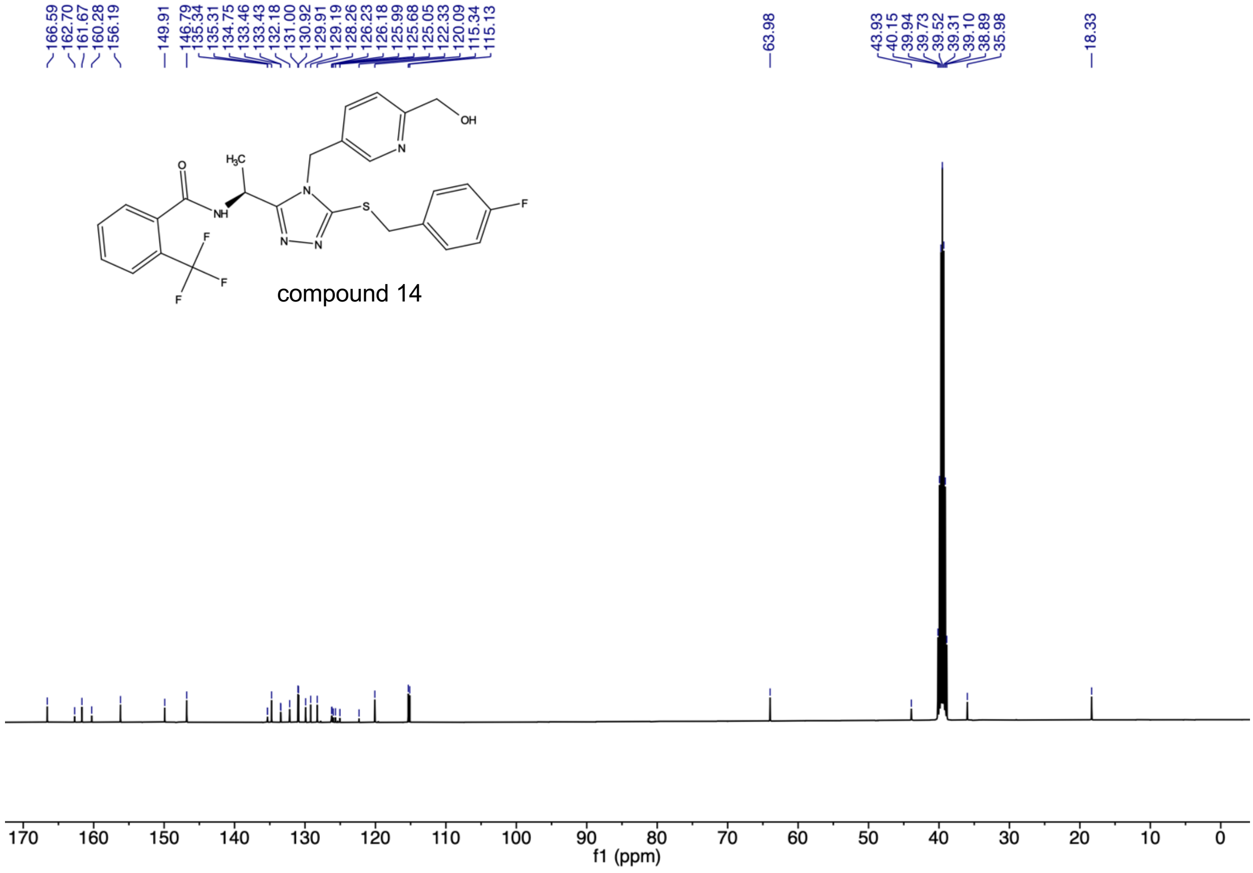
166.59
162.70
161.67
160.28
156.19
149.91
146.79
135.34
135.31
134.75
133.46
133.43
132.18
131.00
130.92
129.91
129.19
128.26
126.23
126.18
125.99
125.68
125.05
122.33
120.09
115.34
115.13
63.98
43.93
40.15
39.94
39.73
39.52
39.31
39.10
38.89
35.98
18.33
OH
N
H_3C
O
NH
N
S
F
N
N
F
F
F
compound 14
f1 (ppm)

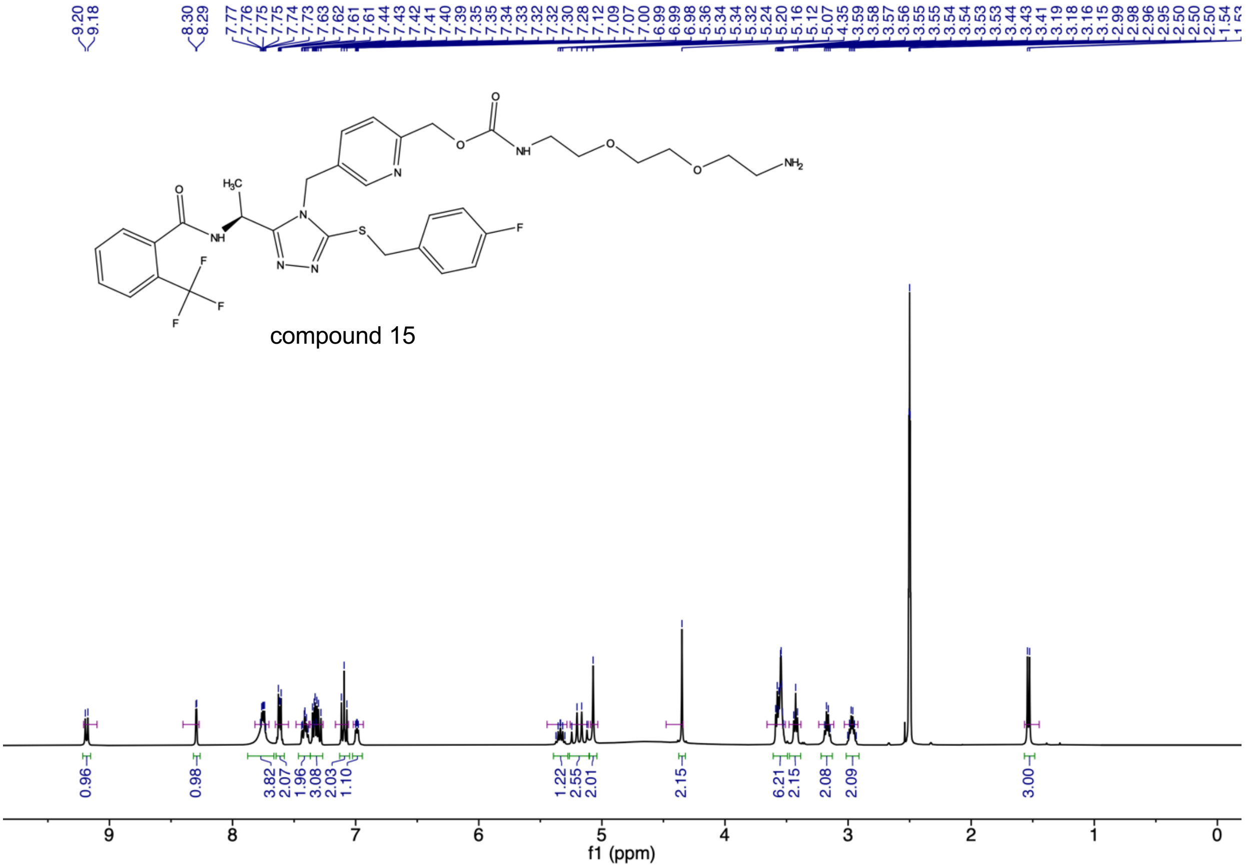
9.20
9.18
8.30
8.29
7.77 7.76 7.75 7.75 7.74 7.73 7.63 7.62 7.61 7.61 7.44 7.43 7.42 7.41 7.40 7.39 7.35 7.35 7.34 7.33 7.32 7.32 7.30 7.28 7.12 7.09 7.07 7.00 6.99 6.99 6.98 5.36 5.34 5.34 5.32 5.24 5.20 5.16 5.12 5.07 4.35 3.59 3.58 3.57 3.56 3.55 3.55 3.54 3.53 3.53 3.44 3.43 3.41 3.19 3.18 3.16 3.15 2.99 2.98 2.96 2.95 2.50 2.50 2.50 1.54
O
NH
O
O
NH2
N
H3C
O
NH
N
S
F
N
N
F
F
F
compound 15
0.96
0.98
3.82 2.07 1.96 3.08 2.03 1.10
1.22 2.55 2.01
2.15
6.21 2.15
2.08
2.09
3.00
9 8 7 6 5 4 3 2 1 0
f1 (ppm)

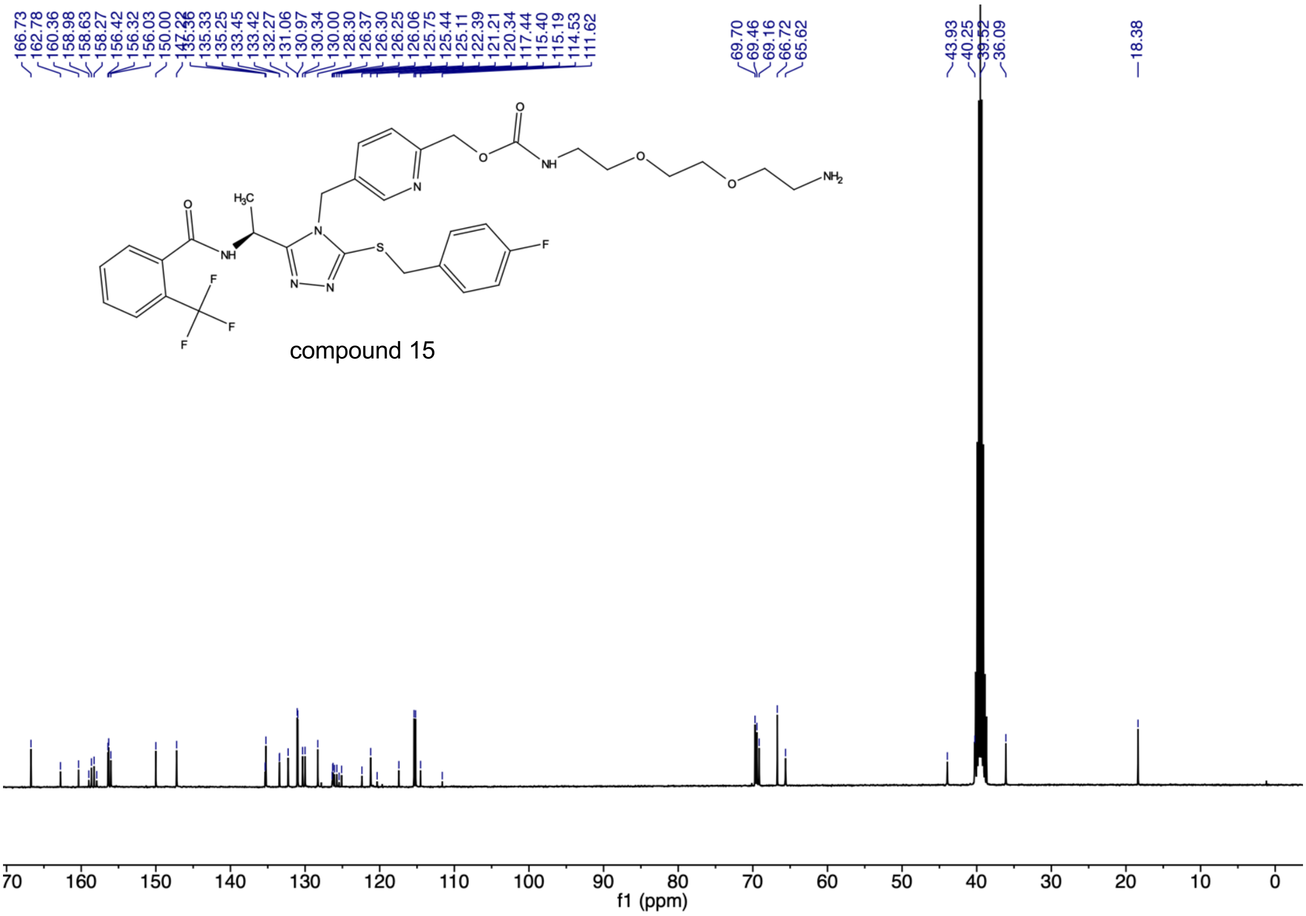
166.73 162.78 160.36 158.98 158.63 158.27 156.42 156.32 156.03 150.00 147.22 135.36 135.33 135.25 133.45 133.42 132.27 131.06 130.97 130.34 130.00 128.30 126.37 126.30 126.25 126.06 125.75 125.44 125.11 122.39 121.21 120.34 117.44 115.40 115.19 114.53 111.62
69.70 69.46 69.16 66.72 65.62
43.93 40.25 39.52 36.09
18.38
O
NH
O
O
NH2
N
H3C
O
NH
N
S
F
N
N
F
F
F
compound 15
170 160 150 140 130 120 110 100 90 80 70 60 50 40 30 20 10 0
f1 (ppm)

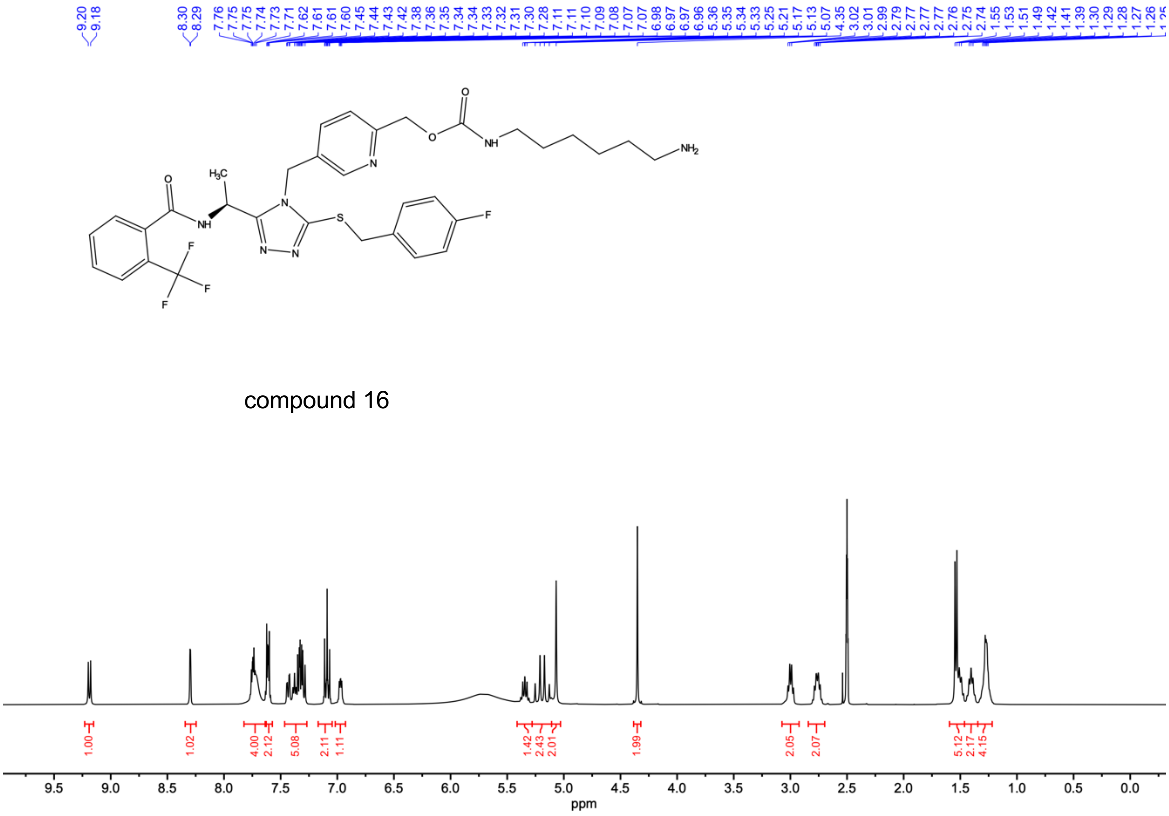

compound 16

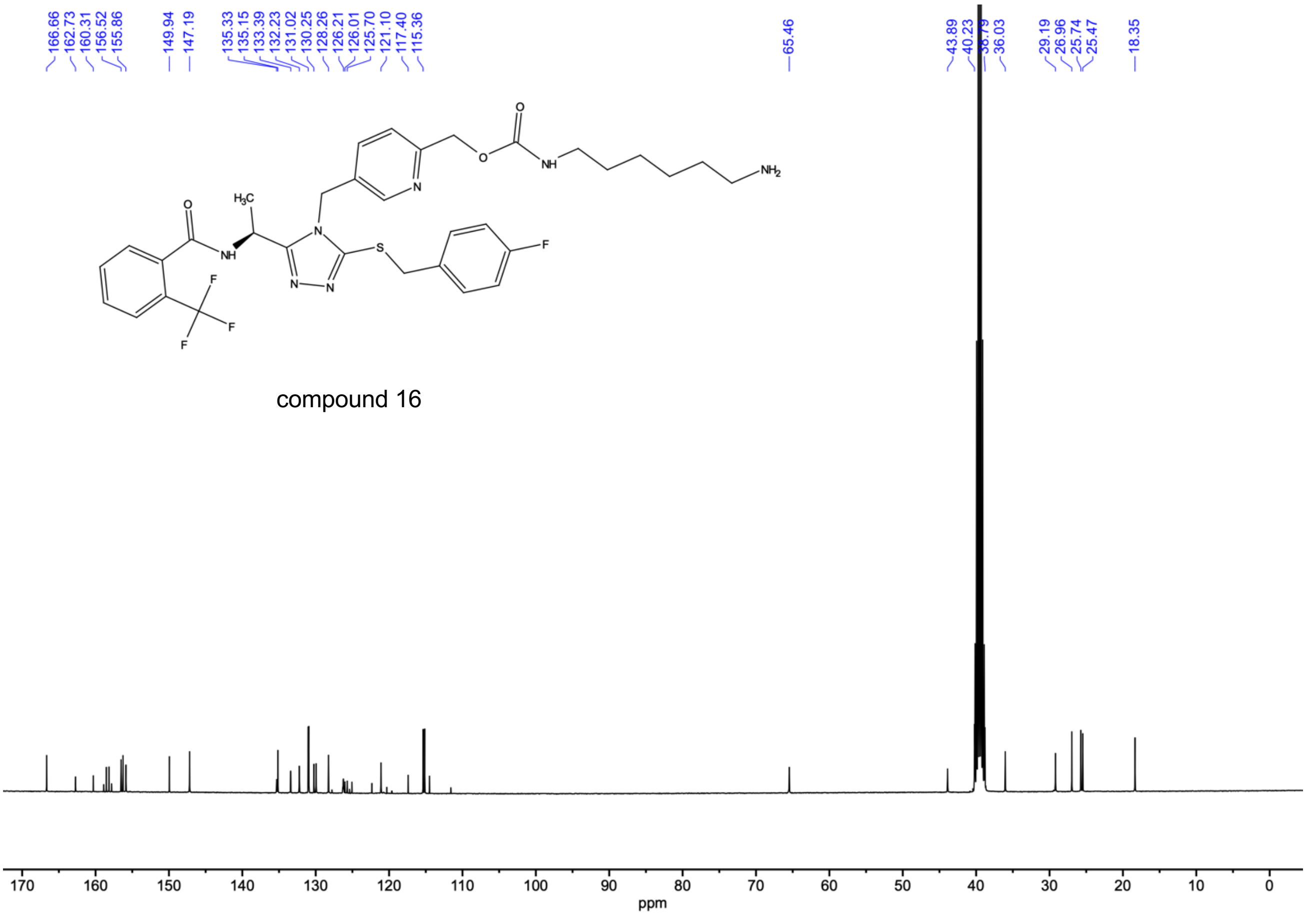

compound 16

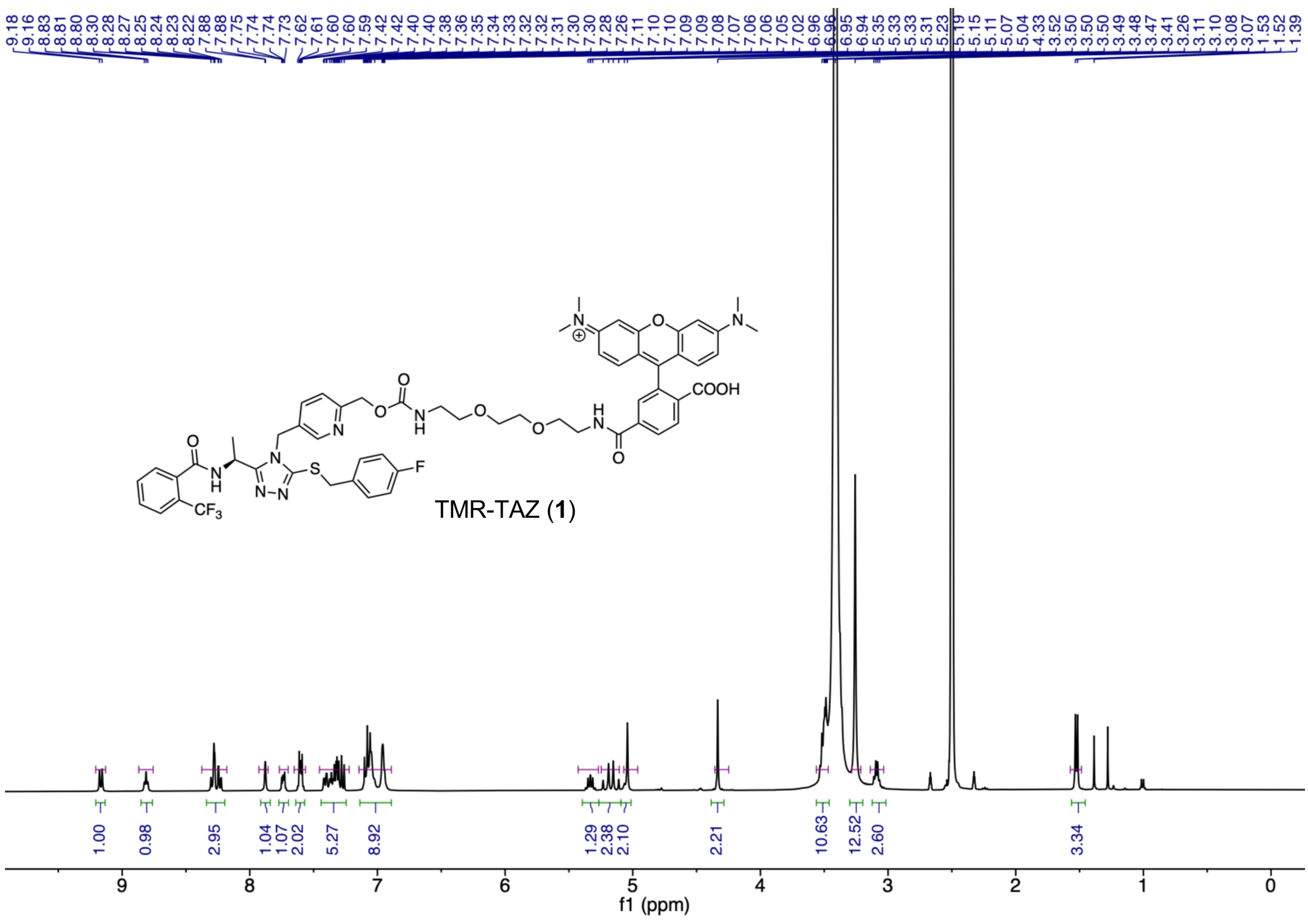

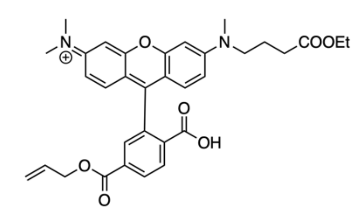

compound 17

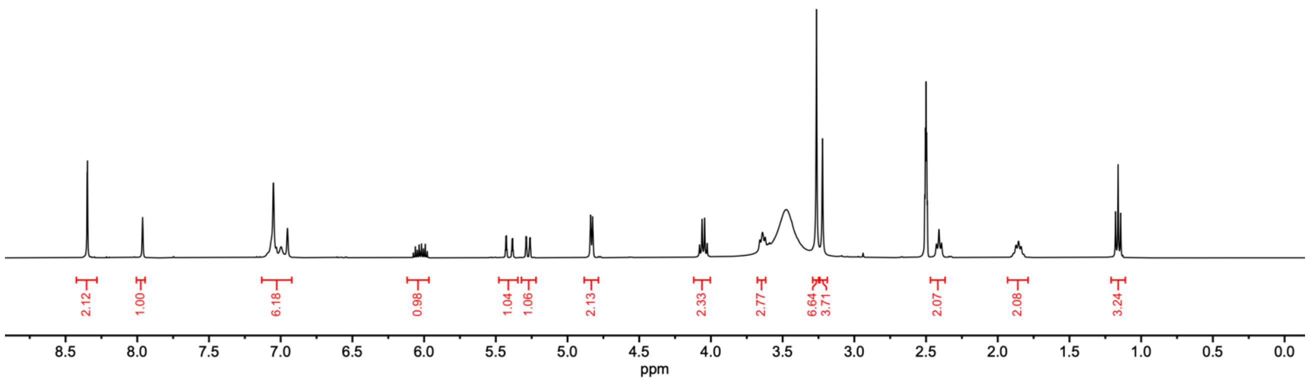

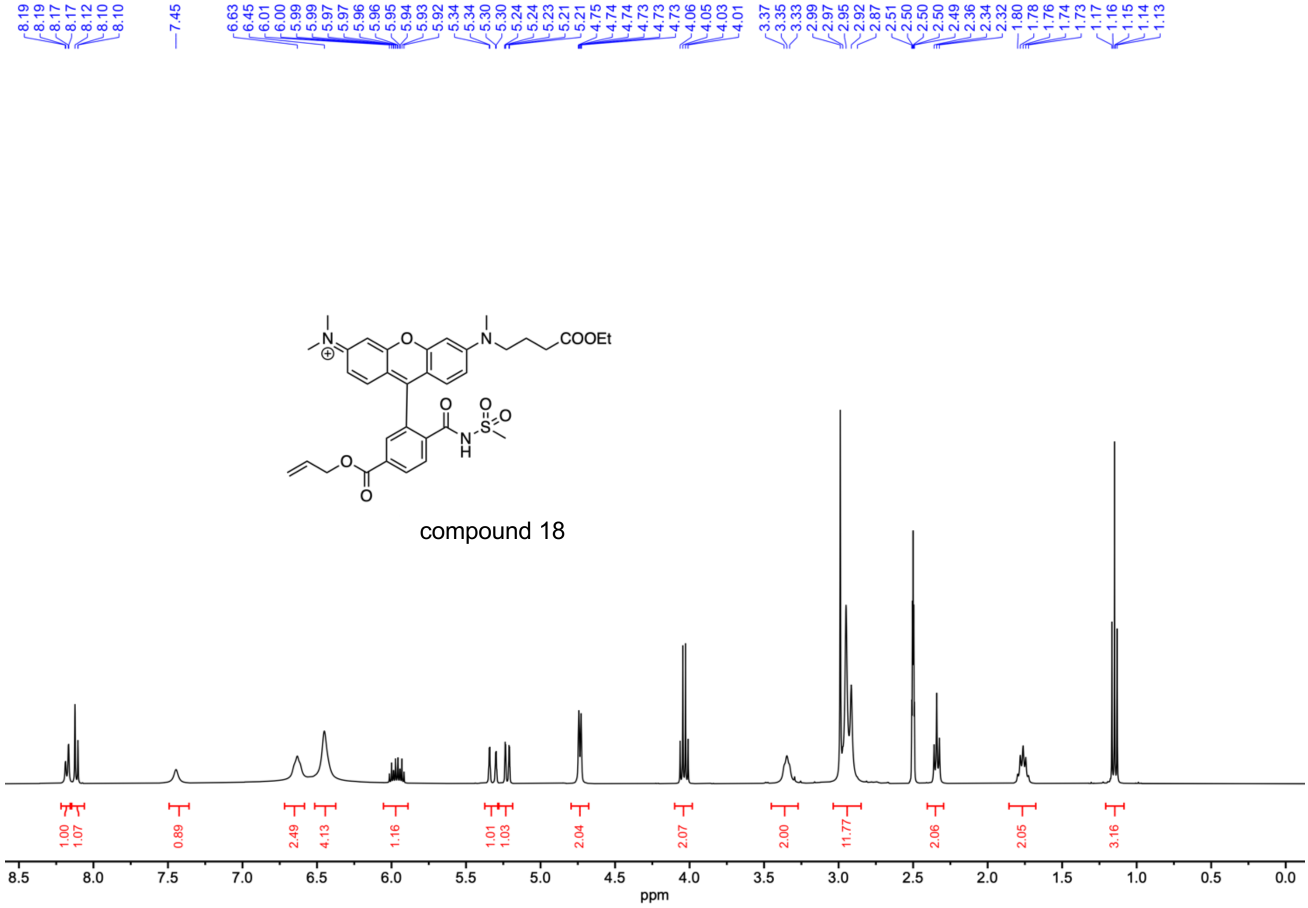

compound 18

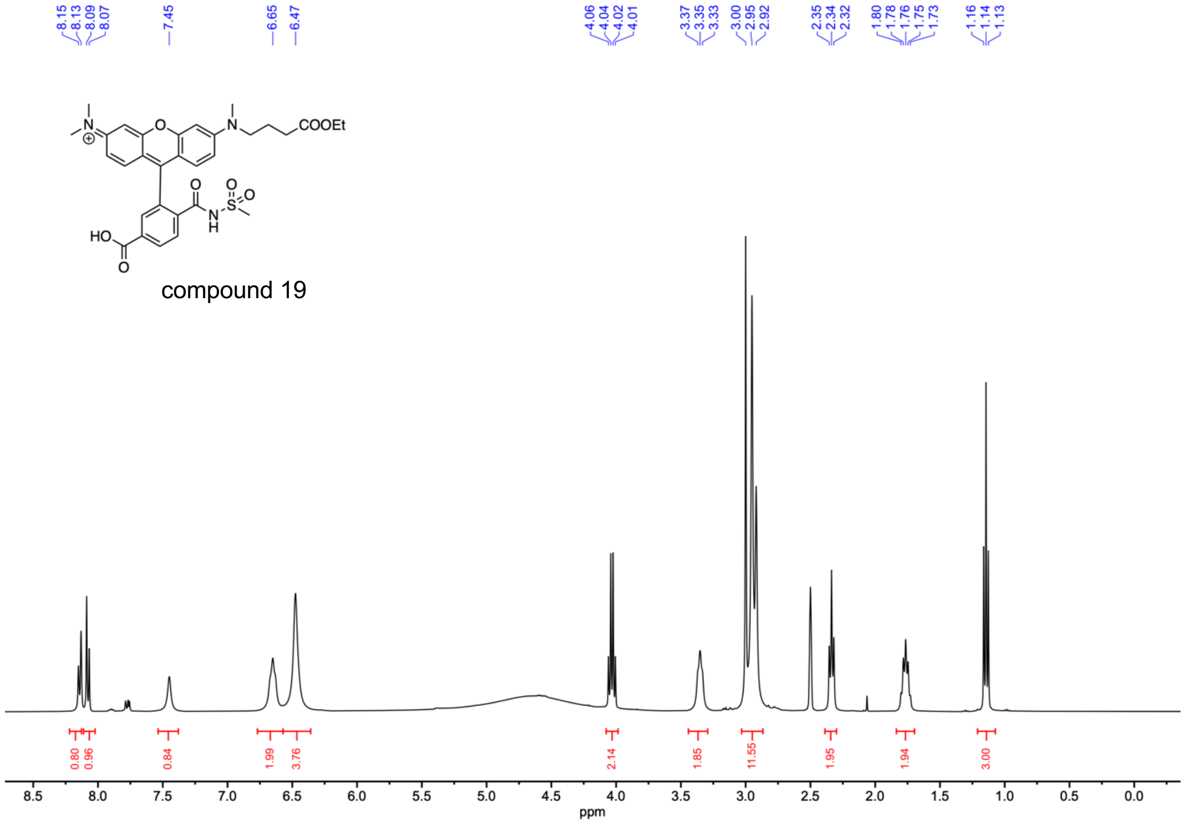

compound 19

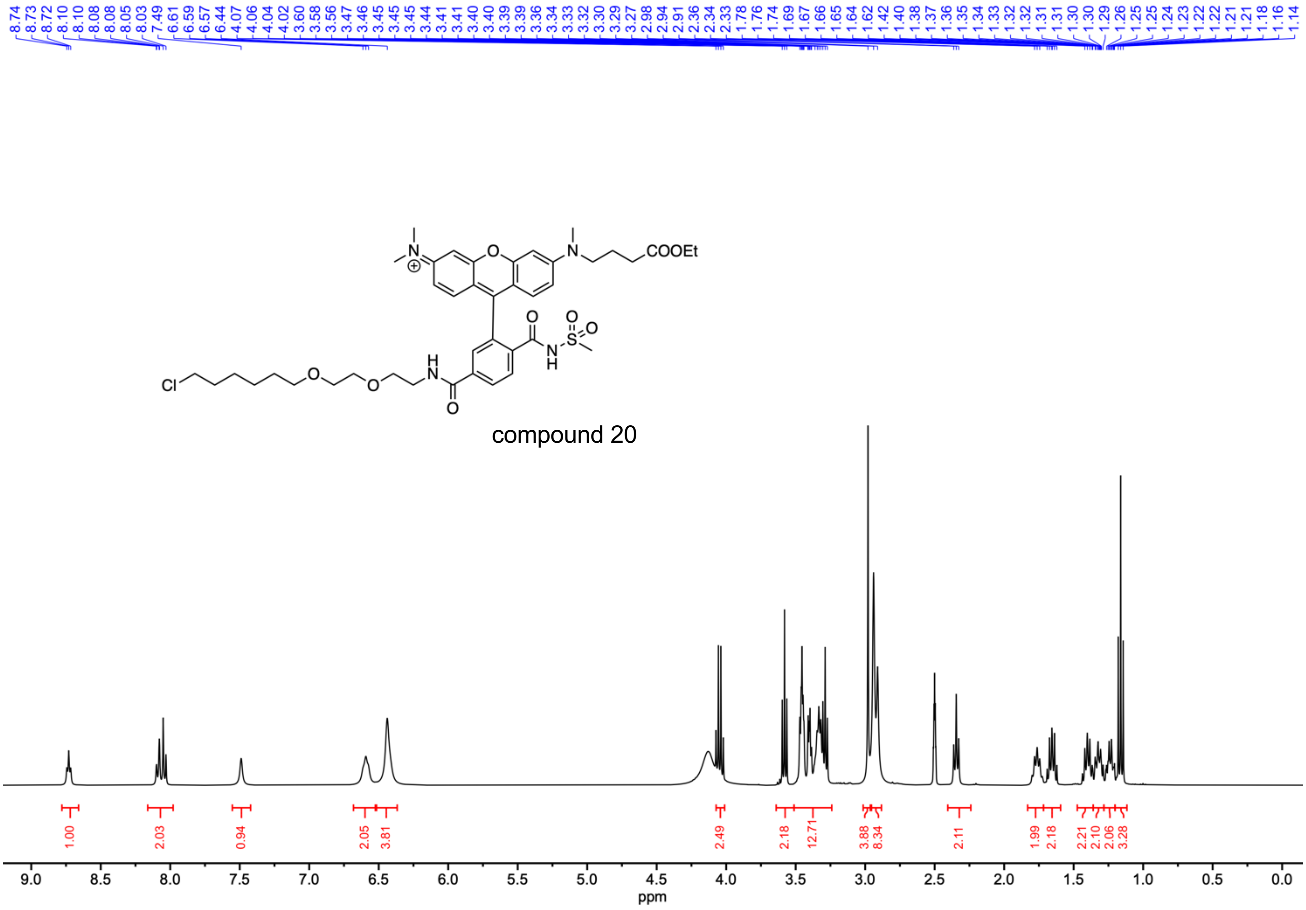
8.74 8.73 8.72 8.10 8.10 8.08 8.05 8.03 7.49 6.61 6.59 6.57 6.44 4.07 4.06 4.04 4.02 3.60 3.58 3.56 3.47 3.46 3.45 3.45 3.45 3.44 3.41 3.41 3.40 3.40 3.39 3.39 3.36 3.34 3.33 3.32 3.30 3.29 3.27 2.98 2.94 2.91 2.36 2.34 2.33 1.78 1.76 1.74 1.69 1.67 1.66 1.65 1.64 1.62 1.42 1.40 1.38 1.37 1.36 1.35 1.34 1.33 1.32 1.32 1.31 1.30 1.30 1.29 1.26 1.25 1.24 1.23 1.22 1.22 1.21 1.18 1.16 1.14
COOEt
Cl
compound 20
1.00 2.03 0.94 2.05 3.81 2.49 2.18 12.71 3.88 8.34 2.11 1.99 2.18 2.21 2.10 2.06 3.28
9.0 8.5 8.0 7.5 7.0 6.5 6.0 5.5 5.0 4.5 4.0 3.5 3.0 2.5 2.0 1.5 1.0 0.5 0.0
ppm

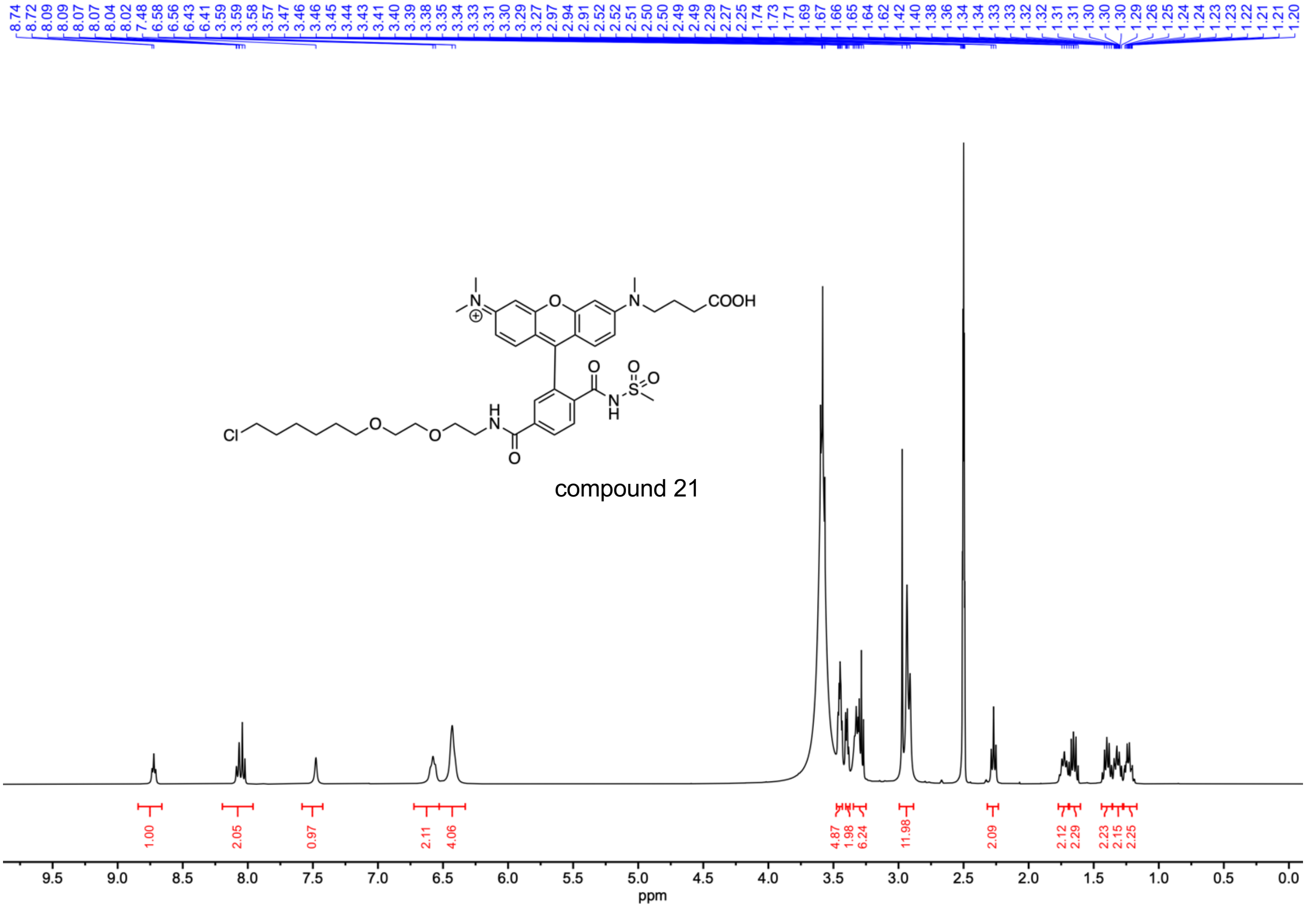
8.74 8.72 8.09 8.07 8.04 8.02 7.48 6.58 6.56 6.43 6.41 3.59 3.58 3.57 3.47 3.46 3.45 3.44 3.43 3.41 3.40 3.39 3.38 3.35 3.34 3.33 3.31 3.30 3.29 3.27 2.97 2.94 2.91 2.52 2.52 2.51 2.50 2.50 2.49 2.49 2.29 2.27 2.25 1.74 1.73 1.71 1.69 1.67 1.66 1.65 1.64 1.62 1.42 1.40 1.38 1.36 1.34 1.34 1.33 1.33 1.32 1.32 1.31 1.31 1.30 1.30 1.29 1.26 1.25 1.24 1.24 1.23 1.23 1.22 1.21 1.21 1.20
COOH
Cl
compound 21
1.00 2.05 0.97 2.11 4.06 4.87 1.98 6.24 11.98 2.09 2.12 2.29 2.23 2.15 2.25
9.5 9.0 8.5 8.0 7.5 7.0 6.5 6.0 5.5 5.0 4.5 4.0 3.5 3.0 2.5 2.0 1.5 1.0 0.5 0.0
ppm

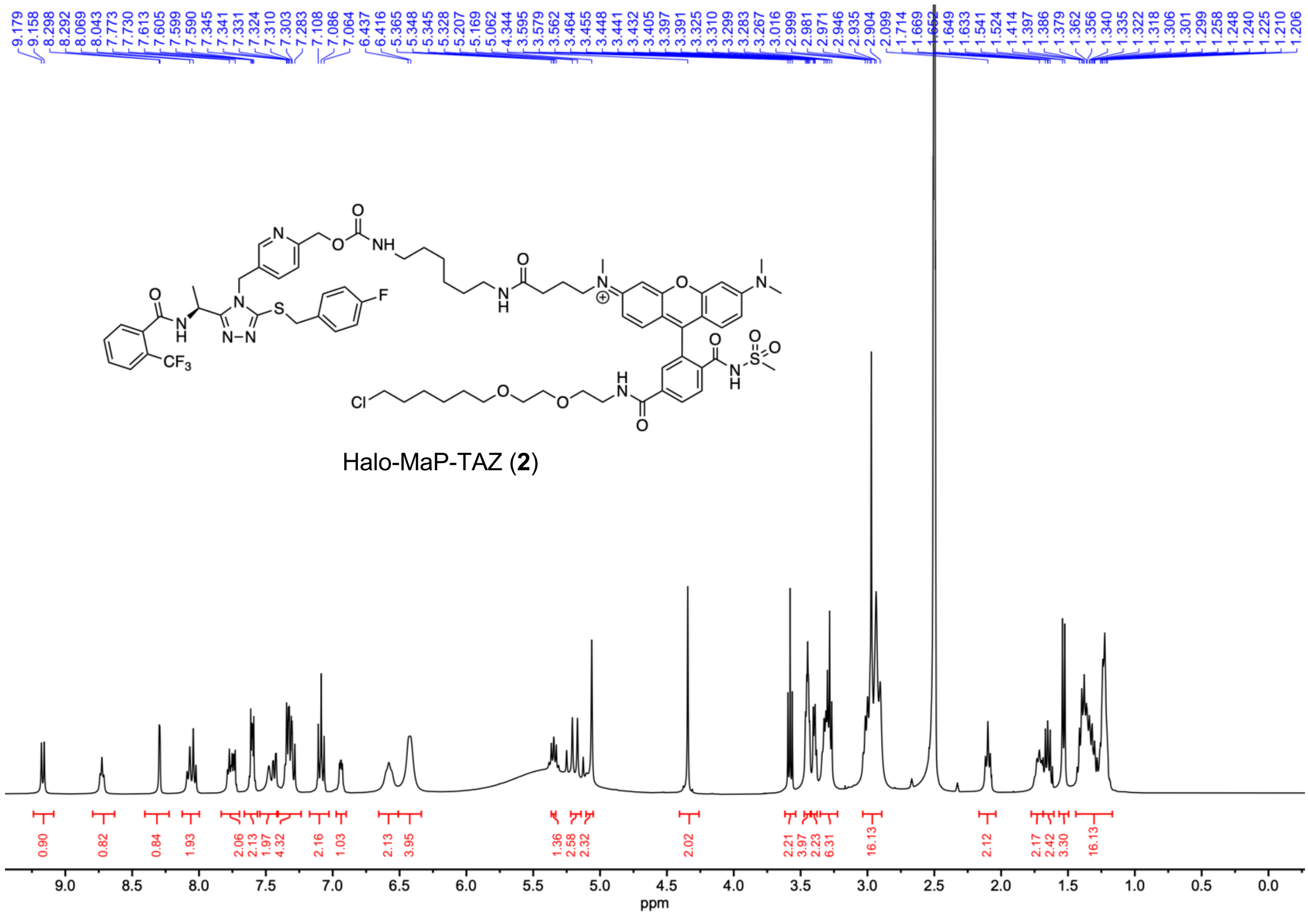
Halo-MaP-TAZ (2)
ppm
