# Supplementary material for: Probing coenzyme A homeostasis with semisynthetic biosensors
Source: Nat Chem Biol. 2022 Oct 31;19(3):346–55. doi: 10.1038/s41589-022-01172-7 (PMC9974488; doi:10.1038/s41589-022-01172-7)
Supplement: Supplementary file 2 — Reporting Summary [file 41589_2022_1172_MOESM2_ESM.pdf]

nature research

Corresponding author(s): Lin Xue, Kai Johnsson

Last updated by author(s): Sep 3, 2022

Reporting Summary

Nature Research wishes to improve the reproducibility of the work that we publish. This form provides structure for consistency and transparency in reporting. For further information on Nature Research policies, see our Editorial Policies and the Editorial Policy Checklist.

Statistics

For all statistical analyses, confirm that the following items are present in the figure legend, table legend, main text, or Methods section.

n/a	Confirmed	
☐	☒	The exact sample size (n) for each experimental group/condition, given as a discrete number and unit of measurement
☐	☒	A statement on whether measurements were taken from distinct samples or whether the same sample was measured repeatedly
☐	☒	The statistical test(s) used AND whether they are one- or two-sided<br>*Only common tests should be described solely by name; describe more complex techniques in the Methods section.*
☒	☐	A description of all covariates tested
☐	☒	A description of any assumptions or corrections, such as tests of normality and adjustment for multiple comparisons
☐	☒	A full description of the statistical parameters including central tendency (e.g. means) or other basic estimates (e.g. regression coefficient) AND variation (e.g. standard deviation) or associated estimates of uncertainty (e.g. confidence intervals)
☐	☒	For null hypothesis testing, the test statistic (e.g. F, t, r) with confidence intervals, effect sizes, degrees of freedom and P value noted<br>*Give P values as exact values whenever suitable.*
☒	☐	For Bayesian analysis, information on the choice of priors and Markov chain Monte Carlo settings
☒	☐	For hierarchical and complex designs, identification of the appropriate level for tests and full reporting of outcomes
☒	☐	Estimates of effect sizes (e.g. Cohen's d, Pearson's r), indicating how they were calculated

Our web collection on statistics for biologists contains articles on many of the points above.

Software and code

Policy information about availability of computer code

Data collection	Plate reader: Tecan Sparkcontrol Method Editor Version 2.2<br>NMR: Bruker TopSpin Version 3.5<br>HRMS: Bruker otofControl Version 4.1, Bruker Hystar Version 4.1 SR2 software<br>Microscopy: Leica LAS X Version 3.5.7.23225 (Confocal) and LASX FLIM/FCS Version 3.5.6<br>Flow cytometry: BD FACSMelody Cell Sorter, BD LSRFortessa X-20 Flow cytometer<br>Gel Imaging: Amersham Typhoon Scanner Control Software Version 2.0<br>LC-MS/MS measurements: Sciex Analyst Version 1.7<br>WesternBlot analysis: Compass for SW Version 4.0.0
Data analysis	General data analysis: OriginPro 2021b (64-bit) SR1 9.8.5.204 (Academic), Microsoft Excel 2016 (Version 16.0.5122.1000)<br>Image analysis: ImageJ2 Version 1.53f with plugin PixFRET 1.5.0, Leica LAS X Version3.5.7.23225 (Confocal) and LASX FLIM/FCS 3.5.6<br>X-ray crystallography: PyMOL Version 2.5.0<br>Chemical synthesis: MestReNova Version 14.2.0<br>LC-MS/MS measurements: Sciex MultiQuant Version 3.0.2<br>Flow cytometry: FlowJo Version 10.4.0, BD FACSDiva Version 9.0<br>WesternBlot analysis: Compass for SW Version 4.0.0

For manuscripts utilizing custom algorithms or software that are central to the research but not yet described in published literature, software must be made available to editors and reviewers. We strongly encourage code deposition in a community repository (e.g. GitHub). See the Nature Research guidelines for submitting code & software for further information.

Data

Policy information about availability of data

All manuscripts must include a data availability statement. This statement should provide the following information, where applicable:

- Accession codes, unique identifiers, or web links for publicly available datasets
- A list of figures that have associated raw data
- A description of any restrictions on data availability

The crystal structures for mtPanK and ecPanK were previously reported with PDB ID of 4BFU and 1ESM, respectively, in the Protein Data Bank (PDB). Plasmids encoding the sensor proteins and genes for proteins in the CoA synthesis pathway, the fluorescent probe Halo-MaP-TAZ, and the intermediate compounds should be addressed to K.J.. The data supporting the findings of this study are available within the paper and its Supplementary Information and are available from the corresponding authors upon reasonable request.

Field-specific reporting

Please select the one below that is the best fit for your research. If you are not sure, read the appropriate sections before making your selection.

☒ Life sciences ☐ Behavioural & social sciences ☐ Ecological, evolutionary & environmental sciences

For a reference copy of the document with all sections, see nature.com/documents/nr-reporting-summary-flat.pdf

Life sciences study design

All studies must disclose on these points even when the disclosure is negative.

Sample size	Sample size was based on experience in prior studies for other fluorescent sensors [PLoS ONE, 2017,12(11): e0187481; PNAS,2008, 105 (49) 19264-19269.]. Data for in vitro titrations were from three independent replicates and shown as the mean ± SD. Cell imaging experiments were performed in four imaging dishes (independently treated), from which n = 4, 5 or 6 field of views (FOVs) were obtained with > 50 cells per FOV, which were used to calculate normalized FRET ratios. The detailed n and p values were provided in the Supplementary Table 8.
Data exclusions	No data was excluded.
Replication	Unless stated in figure legends or method sections, all experiments were done at least twice and the reproduction were successful. The number of samples and independent biological experiments are specified in the manuscript. All replicates were successful.
Randomization	The is not relevant to the study, as all experiments were done using human cell lines. No experiments involved allocation of different samples, organisms, or participants into experimental groups.
Blinding	Blinding was not relevant to the study because no experiments involved allocation of different samples, organisms, or participants into experimental groups.

Reporting for specific materials, systems and methods

We require information from authors about some types of materials, experimental systems and methods used in many studies. Here, indicate whether each material, system or method listed is relevant to your study. If you are not sure if a list item applies to your research, read the appropriate section before selecting a response.

Materials & experimental systems

n/a	Involved in the study	
☐	☒	Antibodies
☐	☒	Eukaryotic cell lines
☒	☐	Palaeontology and archaeology
☒	☐	Animals and other organisms
☒	☐	Human research participants
☒	☐	Clinical data
☒	☐	Dual use research of concern

Methods

n/a	Involved in the study	
☒	☐	ChIP-seq
☐	☒	Flow cytometry
☒	☐	MRI-based neuroimaging

Antibodies

Antibodies used	Anti-Rabbit Detection Module, DM-001 and Anti-Mouse Detection Module, DM-002, ProteinSimple.<br>Rabbit polyclonal anti-GAPDH (NB300-322, 1:1000, Novus Biologicals, Littleton, CO),<br>Rabbit polyclonal anti-Nudt8 (PA5-59493, 1:50, Thermo Fisher Scientific)<br>Rabbit polyclonal anti-PanK2 (PA5-52563, 1:20, Thermo Fisher Scientific)<br>Mouse monoclonal anti-COASY (WH0080347M1-100UG, 1:500, Sigma-Aldrich)

	Rabbit polyclonal anti-ACLY (PA5-29497, 1:100, Thermo Fisher Scientific).<br>Mouse IgG (H+L) Highly Cross-Adsorbed Secondary Antibody (A32728, 1:1000, Thermo Fisher Scientific).
Validation	All antibodies were commercially available and were validated by manufactures, in previous publications and in this study.<br>Rabbit polyclonal anti-GAPDH antibody was validated using NIC/c-Src+/+ cells. (https://www.nature.com/articles/s41467-019-10681-4)<br>Rabbit polyclonal anti-Nudt8 antibody was validated using RT-4 cells by immunofluorescent staining and on U251 cells by western blot.<br>Rabbit polyclonal anti-PanK2 antibody was validated using U-2 OS cells by immunofluorescent staining and on HEK293 cell line by western blot.(https://www.thermofisher.com/antibody/product/NUDT8-Antibody-Polyclonal/PA5-59493)<br>Rabbit polyclonal anti-ACLY antibody was validated using Hela cells by immunofluorescent staining and on Hela, A549, PC-3, SK-OV-3, A-431 Jurkat cells by western blot. (https://www.thermofisher.com/antibody/product/ATP-Citrate-Lyase-Antibody-Polyclonal/PA5-29497)<br>Mouse monoclonal anti-COASY antibody was validated using HEK293T and A-431, cells by by western blot. (https://www.sigmaaldrich.com/US/en/product/sigma/wh0080347m1)<br>Mouse IgG (H+L) Highly Cross-Adsorbed Secondary Antibody was validated using A549, HeLa, U-2 OS cells by immunofluorescent staining. (https://www.thermofisher.com/antibody/product/Goat-anti-Mouse-IgG-H-L-Highly-Cross-Adsorbed-Secondary-Antibody-Polyclonal/A32728)<br>The scecondary antibodies from ProteinSimple were validated by other publications. (Gee P et al. (2020) Extracellular nanovesicles for packaging of CRISPR-Cas9 protein and sgRNA to induce therapeutic exon skipping Nat Commun, 11 (1) :1334. Lee CAA et al. (2020) Targeting the ABC transporter ABCB5 sensitizes glioblastoma to temozolomide-induced apoptosis through a cell-cycle checkpoint regulation mechanism J Biol Chem, 295 (22) :7774-7788. Mackey E et al. (2020) Perinatal androgens organize sex differences in mast cells and attenuate anaphylaxis severity into adulthood Proc Natl Acad Sci U S A, 117 (38) :23751-23761.)

Eukaryotic cell lines

Policy information about cell lines

Cell line source(s)	U2-OS, HeLa, HepG2 cell lines were ordered from Leibniz Institute DSMZ-German Collection of Microorganisms and Cell Cultures GmbH.<br>Flp-In-T-REx-293 cell line was ordered from Thermo Fisher Scientific.<br>The 293 cells stably expressing sensor proteins were generated according to the standard protocol from Thermo Fisher Scientific.
Authentication	Cell lines were not further authenticated.
Mycoplasma contamination	All the cell lines have been tested and are negative.
Commonly misidentified lines<br>(See ICLAC register)	Not applicable as no commonly misidentified cell lines were used.

Flow Cytometry

Plots

Confirm that:

☒ The axis labels state the marker and fluorochrome used (e.g. CD4-FITC).

☒ The axis scales are clearly visible. Include numbers along axes only for bottom left plot of group (a 'group' is an analysis of identical markers).

☒ All plots are contour plots with outliers or pseudocolor plots.

☒ A numerical value for number of cells or percentage (with statistics) is provided.

Methodology

Sample preparation	The detailed information was provided in Methods section of the manuscript.<br>To measure intracellular [ATP] changes, the ATeam sensors were used.57 ATeam sensors were transiently expressed in HEK293 cells either localized to the cytosol or the inner membrane of mitochondria. The treatment with 1.0 µM PZ-2891 was performed for 12 h during the transfection of the sensors. Subsequently the medium was exchanged and the cells were treated with 1.0 µM PZ-2891 for additional 12 h. Then the cells were washed and resuspended in PBS containing 2% FBS (FACS buffer). The 10 mM 2-DG treatment was performed 24 h post transfection whereby the cells were washed with growth medium without glucose for 30 min prior the treatment. The cells were resuspended in 10 mM 2-DG prepared in FACS buffer and incubated for 30min prior analysis. The experiments were measured at the BD LSRFortessa X-20 Flow Cytometer (Becton, Dickinson and Company, Franklin Lakes, NJ, USA) using the software BD FACSDiva. For each replicate 8000 events were analyzed. The following settings were used to record the donor, FRET and acceptor fluorescence: BV421 (ex 405 nm; em 450/50 nm) for CFP channel, BV510 (ex405nm; em 525/50) for FRET channel and FITC (ex 488 nm; em 530/30 nm) for YFP. Gating strategy involved the removal of dead cells and debris (SSC-A vs FCS-A) and selection of the cell population expressing the sensors (CFP vs YFP). The gated populations in the different conditions were analyzed by determining the mean of their FRET/CFP ratio. The final results are presented as violin plots from two independent biological experiments (Supplementary Fig. 12).<br>To measure intracellular [CoA] changes, the cytosolic CoA-SnifitV97T and mitochondrial CoA-SnifitG41S were used. HEK293 cells stably expressing sensor protein were plated in 12-well plates and cultured in full growth medium at 37 oC, 5% CO2. For

PanK3-overexpression, the cells were transfected with a plasmid encoding PANK3 gene and the empty plasmid was used as a negative control. For the PZ-2891, HoPan, and PPanSH treatments, the cells were directly incubated with the 1.0 µM PZ-2891, 400 µM HoPan or 100 µM PPanSH, respectively. Then, the cells were washed once with FACS buffer and were resuspended in this buffer. 10,000 cells were analyzed on a FACSMelody Cell Sorter (BD Biosciences). The following settings were used to record the donor, FRET and acceptor fluorescence: FITC (ex 488 nm; em 527/32 nm) for GFP channel, PerCP (ex 488 nm; em 700/54 nm) for FRET channel and PE-Cy5 (ex 561 nm; em 697/58 nm) for MaP channel. HEK293 cells were used as blank control. The data was analyzed in FlowJo software. Gating strategy involved the removal of dead cells and debris (SSC-A vs FCS-A) and selection of the labeled cell population (GFP vs MaP). The gated populations in the different conditions were analyzed by determining the mean of their GFP/MaP ratio. The final results are presented as violin plots from three independent biological experiments (Supplementary Fig. 14).

Instrument	BD FACSMelody Cell Sorter, BD LSRFortessa™ X-20 Flow Cytometer
Software	BD FACSChorus Software Version 1.3.3, BD FACSDiva and FLowJo Version 10.4.0
Cell population abundance	Flow cytometry in our study was not used to determine relative abundances among populations.
Gating strategy	The events with low FSC and SSC and those with low FCS and high SSC were eliminated. The boundaries between labeled and non-labeled populations were defined by blank samples (cells without any sensor protein expressed, negative control), donor-only and acceptor-only samples (positive control).

☒ Tick this box to confirm that a figure exemplifying the gating strategy is provided in the Supplementary Information.
