# Supplementary figures and images for: Probing coenzyme A homeostasis with semisynthetic biosensors
Source: Nat Chem Biol. 2022 Oct 31;19(3):346–55. doi: 10.1038/s41589-022-01172-7 (PMC9974488; doi:10.1038/s41589-022-01172-7)

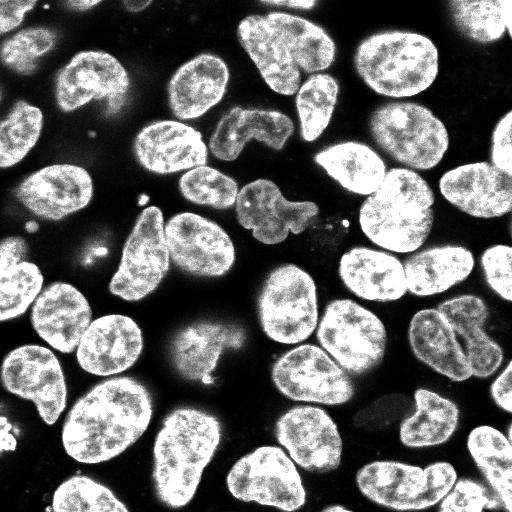

Supplement: Source Data Extended Data Fig. 6 — Source imaging data. [file 41589_2022_1172_MOESM12_ESM.zip › extended data fig 6/extend fig6f-j.tif]

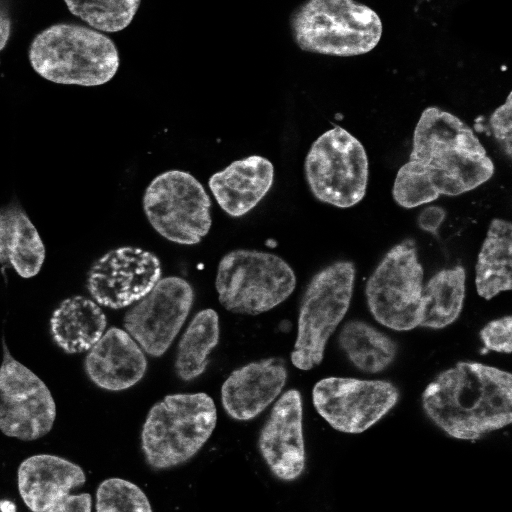

Supplement: Source Data Extended Data Fig. 6 — Source imaging data. [file 41589_2022_1172_MOESM12_ESM.zip › extended data fig 6/extend fig6b-e.tif]

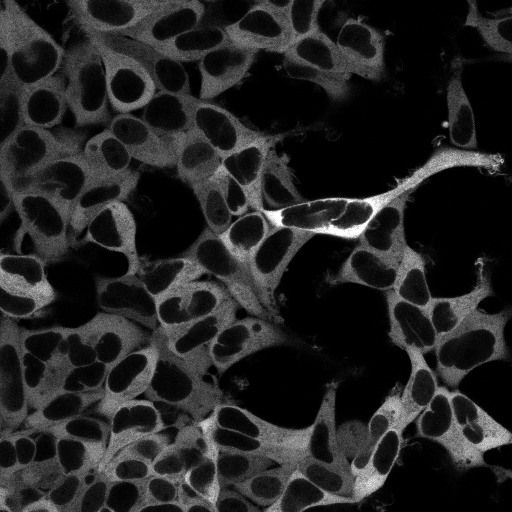

Supplement: Source Data Extended Data Fig. 7 — Source imaging data and calculations. [file 41589_2022_1172_MOESM13_ESM.zip › extended data fig 7/extend fig7c.tif]

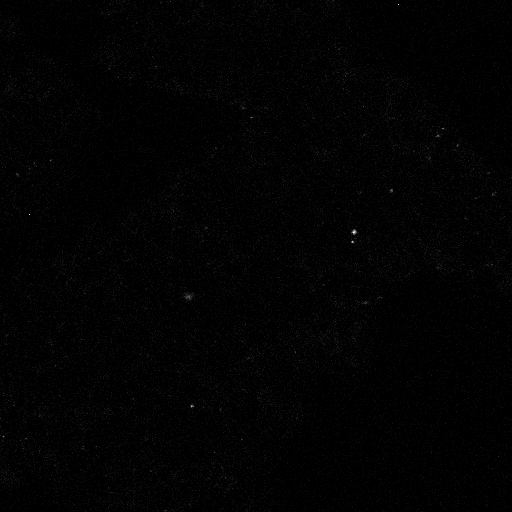

Supplement: Source Data Extended Data Fig. 7 — Source imaging data and calculations. [file 41589_2022_1172_MOESM13_ESM.zip › extended data fig 7/extend fig7b.tif]

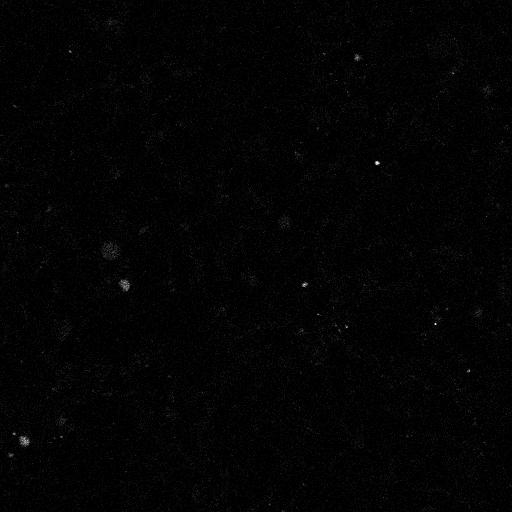

Supplement: Source Data Extended Data Fig. 7 — Source imaging data and calculations. [file 41589_2022_1172_MOESM13_ESM.zip › extended data fig 7/extend fig7a.tif]

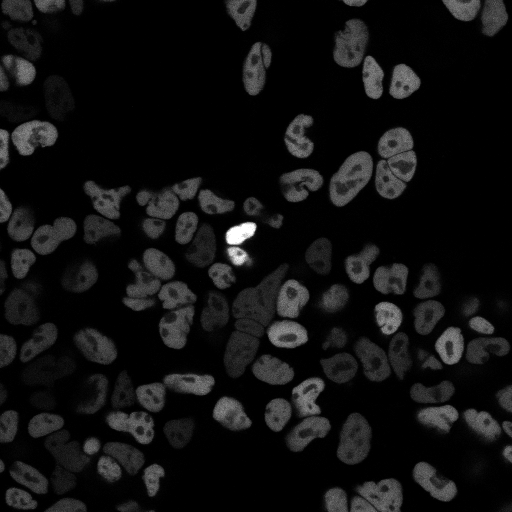

Supplement: Source Data Extended Data Fig. 7 — Source imaging data and calculations. [file 41589_2022_1172_MOESM13_ESM.zip › extended data fig 7/extend fig7e.tif]

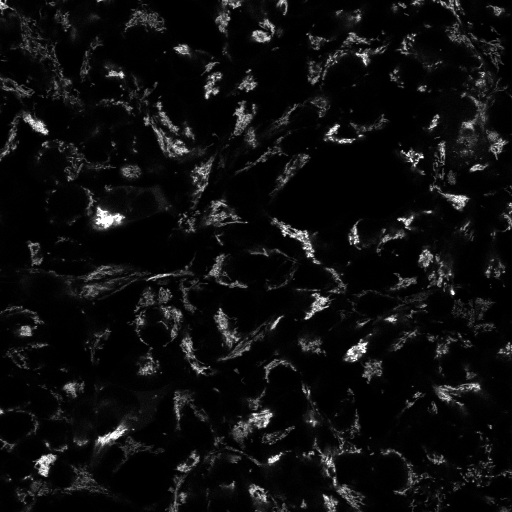

Supplement: Source Data Extended Data Fig. 7 — Source imaging data and calculations. [file 41589_2022_1172_MOESM13_ESM.zip › extended data fig 7/extend fig7d.tif]

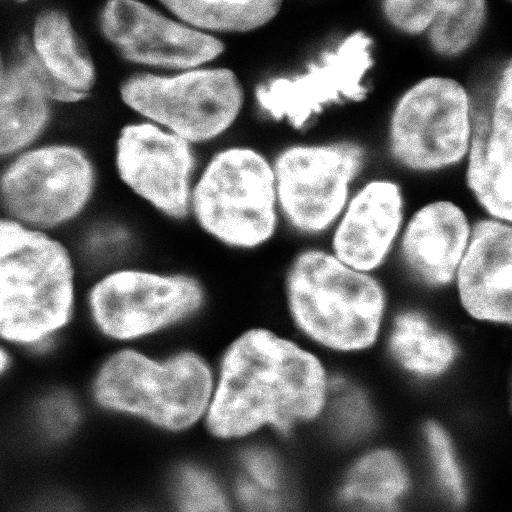

Supplement: Source Data Extended Data Fig. 8 — Source imaging data. [file 41589_2022_1172_MOESM14_ESM.zip › Source_Data_Extended_Data_Fig_8/extend fig8-nudt8.tif]

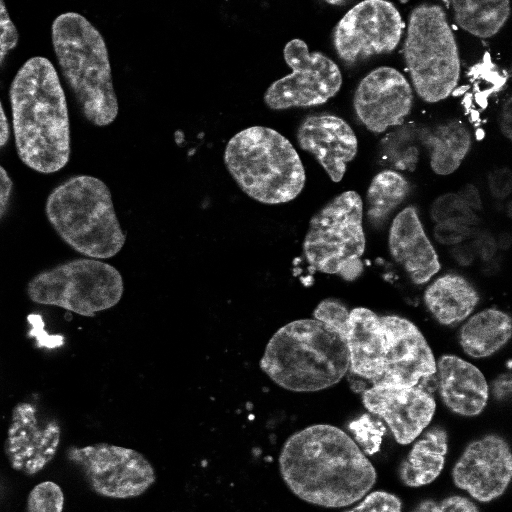

Supplement: Source Data Extended Data Fig. 8 — Source imaging data. [file 41589_2022_1172_MOESM14_ESM.zip › Source_Data_Extended_Data_Fig_8/extend fig8-pank2.tif]

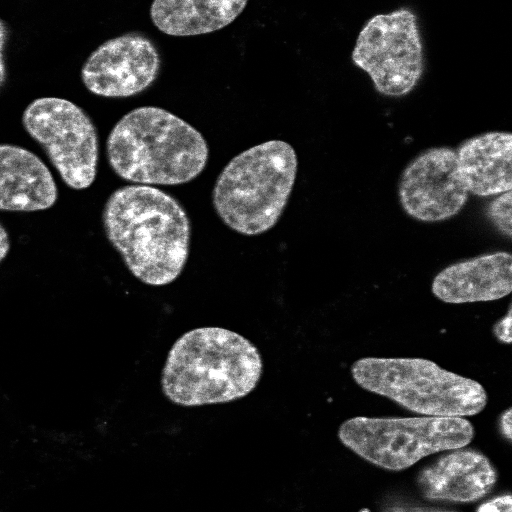

Supplement: Source Data Extended Data Fig. 8 — Source imaging data. [file 41589_2022_1172_MOESM14_ESM.zip › Source_Data_Extended_Data_Fig_8/extend fig8-CG4241.tif]

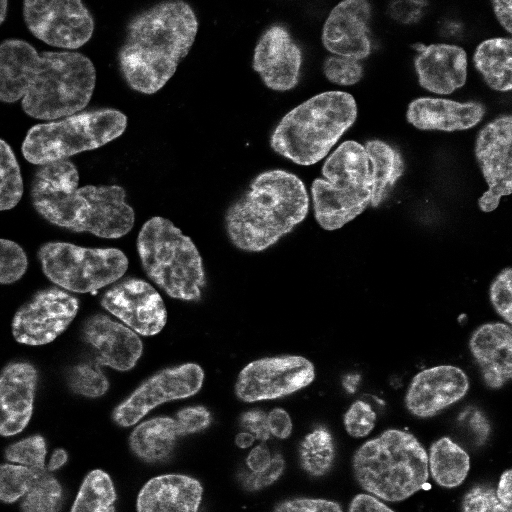

Supplement: Source Data Extended Data Fig. 8 — Source imaging data. [file 41589_2022_1172_MOESM14_ESM.zip › Source_Data_Extended_Data_Fig_8/extend fig8-pank3.tif]

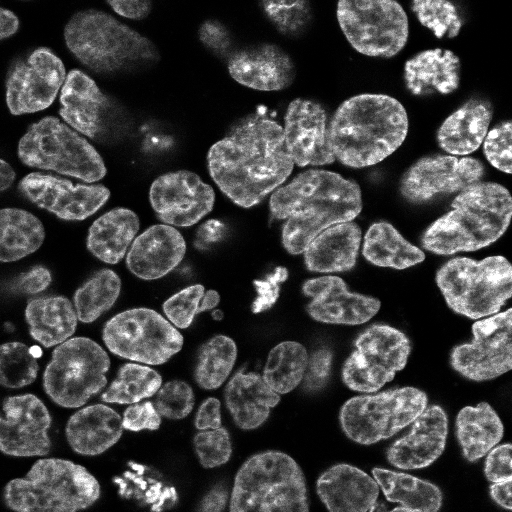

Supplement: Source Data Extended Data Fig. 8 — Source imaging data. [file 41589_2022_1172_MOESM14_ESM.zip › Source_Data_Extended_Data_Fig_8/extend fig8-pank1.tif]
